# Three New and Eleven Known Unusual C25 Steroids: Activated Production of Silent Metabolites in a Marine-Derived Fungus by Chemical Mutagenesis Strategy using Diethyl Sulphate

**DOI:** 10.3390/md12031545

**Published:** 2014-03-13

**Authors:** Ming-Wen Xia, Cheng-Bin Cui, Chang-Wei Li, Chang-Jing Wu

**Affiliations:** 1 Key Laboratory of Structure-Based Drug Design & Discovery of Ministry of Education, School of Traditional Chinese Materia Medica, Shenyang Pharmaceutical University, Shenyang 110016, China; E-Mails: xiamingwen@126.com (M.-W.X.); wucj2009@163.com (C.-J.W.); 2 Beijing Institute of Pharmacology and Toxicology, Beijing 100850, China; E-Mail: sdrlcw@126.com

**Keywords:** C25 steroids, antineocyclocitrinol, 23-*O*-methylantineocyclocitrinol, structure determination, Mo_2_-induced CD, dimolybdenum tetracetate, *Penicillium purpurogenum*, marine-derived fungus, DES mutagenesis

## Abstract

Three new (**1**–**3**) and 11 known (**4**–**14**) C25 steroids with an unusual bicyclo[4.4.1]A/B ring system were isolated by tracing newly produced metabolites in the EtOAc extract of an antitumor mutant AD-1-2 obtained by the diethyl sulphate (DES) mutagenesis of a marine-derived *Penicillium purpurogenum* G59. HPLC-PDAD-UV and HPLC-ESI-MS analyses indicated that the G59 strain did not produce these metabolites and the production of **1**–**14** in the mutant AD-1-2 extract was caused by the activation of silent metabolites in the original G59 strain by DES mutagenesis. The structures of the new compounds, named antineocyclocitrinols A (**1**) and B (**2**) and 23-*O*-methylantineocyclocitrinol (**3**), including their absolute configurations were determined by various spectroscopic methods, especially the NMR and Mo_2_-induced CD analyses. Compounds **1**–**3** provide the first examples of the C25 bicyclo[4.4.1]A/B ring steroids with the *Z*-configuration of 20,22-double bond. All of **1**–**14** weakly inhibited several human cancer cell lines to varying extents. These results provided additional examples for the successful application of the chemical mutagenesis strategy using DES to discover new compounds by activating silent metabolites in fungal isolates and supported also the effectiveness and usefulness of this new strategy.

## 1. Introduction

The C25 steroids with bicyclo[4.4.1]A/B rings are a family of unusual steroids with 16 known family members [1]. Their structures differed mainly in the side chains at C-17 and could be classified into four subtypes: the cyclocitrinol, isocyclocitrinol, neocyclocitrinol, and precyclocitrinol subtypes. The first member of the family cyclocitrinol was isolated from a terrestrial *Penicillium citrinum* and reported as a new sesterpene [2]. However, the structure of cyclocitrinol was later revised to a bicyclo[4.4.1]A/B ring steroid with a *trans*-22,23-double bond, when it was re-isolated from a sponge-derived *Penicillium citrinum* [3]. The cyclocitrinol subtype steroids include cyclocitrinol, 12-hydroxycyclocitrinol, 20-*O*-methylcyclocitrinol, 24-*epi*-cyclocitrinol, 20-*O*-methyl-24-*epi*-cyclo citrinol, and 24-oxiocyclocitrinol. The isocyclocitrinol subtype steroids carry a *trans*-23,24-double bond and consist of isocyclocitrinols A and B, and 22-acetylisocyclocitrinol. Neocyclocitrinol was initially obtained as a mixture of 23,24-epimers with a 20,22-double bond, and the configuration of the 20,22-double bond was not determined [4]. All of four 23,24-diastereomers were isolated later in pure forms, named neocyclocitrinols A–D, respectively, and their absolute configurations were determined, including the *E*-configurations of the 20,22-double bonds [1]. The neocyclocitrinol subtype steroids involve also two methyl derivatives, *erythro*- and *threo*-23-*O*-methylneocyclocitrinols, in addition to neocyclocitrinols A–D. The precyclocitrinol subtype steroid has been known only precyclocitrinol B, which possesses a 20,22-epoxy unit differing from the structures of other subtypes in this class.

Fungal metabolites, especially those derived from marine fungi, are particularly important as rich sources of new drug candidates [5,6] and have continuously attracted considerable attention [5,6,7,8]. As many fungal biosynthetic pathways are silenced in standard culture conditions, activation of the silent pathways may enable access to new metabolites. Indeed, various strategies have been developed to activate silent pathways and elicit metabolite production from fungal isolates [9]. The one strain-many compounds (OSMAC) strategy [10] and chemical epigenetics methodology [11] have been widely applied by microbial chemists to access cryptic secondary metabolites [12], because the culture-based, simple procedures outlined by the strategies are suitable for use by microbial chemists. The ribosome engineering strategy [13] provided also additional simple way to activate silent pathways by introducing drug-resistance mutations in bacteria to discover new antibiotics [14]. We have previously reported a new approach that extended the ribosome engineering strategy to fungi to activate silent metabolites by introducing drug-resistance in *Penicillium purpurogenum* G59 using dimethyl sulfoxide (DMSO) as accessorial agent [15,16]. Recently, we developed another new, practical strategy for activating silent fungal metabolites using *P. purpurogenum* G59 and an old, modified method of diethyl sulphate (DES) mutagenesis [17,18,19]. *P. purpurogenum* G59 is a marine-derived wild type fugal strain initially isolated by our group [20] and originally did not produce any metabolites with antitumor activities in repeated MTT assays using K562 cells [15,16,17,18,19,20]. Using the chemical mutagenesis strategy, we have previously discovered several new antitumor metabolites including some with novel structures by activating silent secondary metabolites in *P. purpurogenum* G59 [17,18,19].

In a continuation of the above mentioned research work, we have undertaken investigations on the bioactive metabolites from an antitumor mutant AD-1-2 that was obtained by the DES mutagenesis of *P. purpurogenum* G59 [17]. Both bioassays and HPLC-photodiode array detector (PDAD)-UV and HPLC-electron spray ionization (ESI)-MS analyses indicated that some kinds of secondary metabolites exist in the EtOAc extract of the mutant AD-1-2 but not in the EtOAc extract of the original G59 strain. This has encouraged us to investigate these secondary metabolites. Indeed, we have obtained different types of compounds by tracing the newly produced metabolites in the mutant AD-1-2 extract. In this paper, we report our research results on the C25 steroids with unusual bicyclo[4.4.1]A/B ring system, including the discovery of three new compounds **1**–**3** in this class and the identification of 11 known steroids **4**–**14** in the same class.

## 2. Results and Discussions

### 2.1. Fermentation, Isolation of **1**–**14**, and Identification of Known Steroids **4**–**14**

Large-scale fermentation and extraction of the mutant AD-1-2 produced an EtOAc extract that inhibited K562 cells with an inhibition rate (IR%) of 58.6% at 100 µg/mL. However, the EtOAc extract of the control G59 strain, obtained by fermentation at the same time and same conditions, did not inhibit the K562 cells (an IR% of 5.6% at 100 µg*/*mL). HPLC-PDAD-UV and HPLC-ESI-MS analyses of the mutant AD-1-2 and the control G59 extracts showed that newly produced C25 steroids existed in the mutant extract and were detected within 41–52 min retention times (see Figures S1 and S2 in the Supplementary Information). The separation of a small amount of the mutant AD-1-2 extract by thin layer chromatography (TLC) followed by the HPLC-PDAD-UV analysis of the obtained TLC fractions indicated that the C25 steroids were enriched in two fractions, B3 and B4 (Figures S3 and S4 in the Supplementary Information). Thus, the separation of the mutant EtOAc extract was performed tracing the newly produced C25 steroids under guidance of bioassays and TLC analyses.

**Figure 1:**
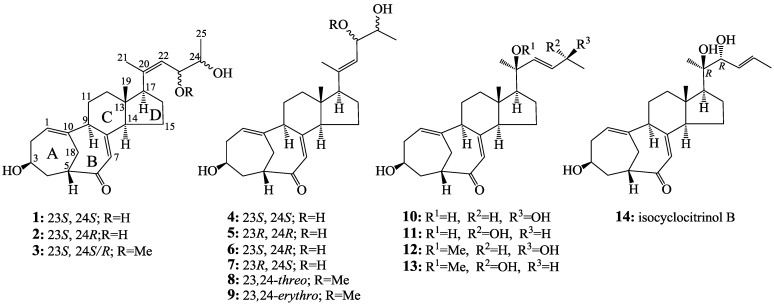
Structures of **1**–**14** from the EtOAc extract of the mutant AD-1-2.

A vacuum liquid chromatography (VLC) separation of the mutant extract on a silica gel column by monitoring the C25 steroids gave a fraction containing these C25 steroids. Further separation of the fraction by repeated column chromatography on Sephadex LH-20 and ODS gave two crude C25 steroid fractions (Figures S5 and S6 in the Supplementary Information). The two fractions were separated by preparative HPLC and semi-preparative HPLC to obtain **1**–**14** (Figure 1).

Among the compounds obtained, structures of three new compounds **1**–**3**, including their absolute configurations, were determined by spectroscopic methods and named antineocyclocitrinols A (**1**) and B (**2**) and 23-*O*-methylantineocyclocitrinol (**3**). On the other hand, 11 known C25 steroids were identified as neocyclocitrinols A (**5**), B (**4**), C (**7**), and D (**6**), *threo*-23-*O*-methylneocyclocitrinol (**8**), *erythro*-23-*O*-methylneocyclocitrinol (**9**), 24-*epi*-cyclocitrinol (**10**), cyclocitrinol (**11**), 20-*O*-methyl-24-*epi*-cyclocitrinol (**12**), 20-*O*-methylcyclocitrinol (**13**), and isocyclocitrinol B (**14**), respectively, by comparison of their physicochemical and spectroscopic data with those reported in the literature [1].

### 2.2. Structure Determination of New Compounds **1**–**3**

**Figure 2:**
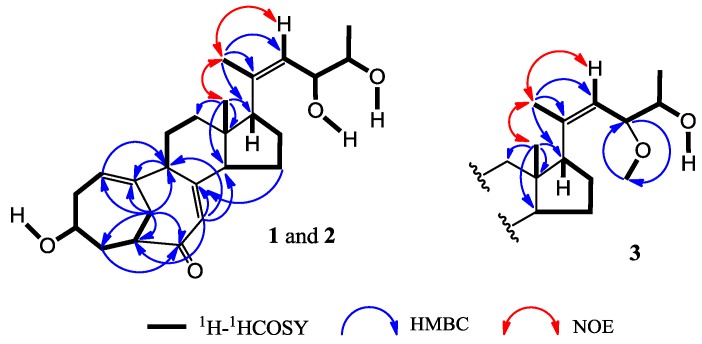
Planar structures and selected NMR data for **1**, **2** and **3**.

Antineocyclocitrinols A (**1**) and B (**2**), both colorless needles (from MeOH), m.p. 178–179 °C, 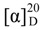 +71.1 (*c* 0.23, MeOH) for **1** and m.p. 201–202 °C, 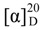 +71.6 (*c* 0.30, MeOH) for **2**, were assigned the molecular formula C_2__5_H_3__6_O_4_ by HRESIMS (measured 401.2689 [M + H]^+^ for **1** and 401.2684 [M + H]^+^ for **2**; calculated for C_2__5_H_3__7_O_4_ [M + H]^+^ 401.2692). Their maximal UV absorptions around 243 nm and the IR absorptions around 1645 cm^−1^ revealed the presence of conjugated carbonyl groups in **1** and **2** [1]. Their IR spectra showed also the absorptions due to the OH and CH_3_/CH_2_ groups (see the UV and IR spectra in the Supplementary Information). Further, the positive ESIMS ion fragments detected at *m*/*z* 383 [M − H_2_O + H]^+^, 365 [M − 2H_2_O + H]^+^, and 347 [M − 3H_2_O + H]^+^ for both **1** and **2** suggested the presence of three hydroxyl groups in their structures [4]. In the ^1^H and ^13^C NMR spectra (the NMR spectra in the Supplementary Information), **1** and **2** showed ^1^H and ^13^C NMR signals that closely resembled the signals from the known steroids **4**–**7**, except several proton and carbon signals from the structural parts around the side chains at C-17 in **1** and **2** differed slightly (Table 1 and Table 2). These NMR data indicated that the planar structures of **1** and **2** are the same as **4**–**7**, but the stereo structures of the side chain moieties are slightly different. This was confirmed by detailed analyses of their DEPT, ^1^H-^1^H COSY, HMQC, and HMBC spectra (see Table S1 for **1** and Table S2 for **2** in the Supplementary Information) to establish their planar structures (Figure 2). The stereochemistry of **1** and **2** including their absolute configurations were determined as follows.

**Table 1:** 400 MHz ^1^H NMR Data of **1**, **2**, **4**–**7**, **10** and **11** in DMSO-*d*_6_ (δ_H_, *J* in Hz) ^a^.

Position	1 ^b^	2 ^b^	4	5	6	7	10	11
1	5.55 dd (8.0, 6.4)	5.55 dd (8.2, 6.4)	5.56 dd (8.2, 6.3)	5.55 dd (8.1, 6.4)	5.55 dd (8.1, 6.2)	5.55 dd (8.1, 6.3)	5.53 dd (8.4, 6.4)	5.53 dd (8.3, 6.4)
2α	2.07 m	2.07 m	2.07 m	2.07 m	2.07 m	2.08 m	2.07 m	2.07 m
β	2.34 ddd (13.0, 11.4, 6.4)	2.34 ddd (13.2, 11.2, 6.4)	2.34 ddd (13.0, 11.5, 6.3)	2.34 ddd (13.2, 11.2, 6.4)	2.34 m	2.34 ddd (12.7, 11.3, 6.3)	2.33 ddd (13.1, 11.3, 6.3)	2.33 ddd (13.0, 11.2, 6.3)
3	3.12 m	3.12 m	3.12 m	3.12 m	3.12 m	3.13 m	3.11 m	3.11 m
4α	2.63 br d (13.1)	2.63 br d (12.8)	2.63 br d (13.2)	2.63 br d (13.1)	2.63 br d (13.0)	2.64 br d (13.1)	2.62 br d (13.0)	2.62 br d (12.9)
β	1.53 m	1.52 m	1.53 m	1.52 m	1.52 m	1.53 m	1.51 m	1.51 m
5	2.69 m	2.69 m	2.68 m	2.68 m	2.68 m	2.68 m	2.67 m	2.67 m
7	5.43 s	5.43 s	5.42 s	5.42 s	5.42 s	5.42 s	5.37 s	5.38 s
9	2.86 dd (12.0, 5.5)	2.86 dd (11.3, 5.6)	2.85 dd (11.7, 5.4)	2.84 dd (11.8, 5.6)	2.85 dd (12.0, 5.4)	2.85 dd (11.4, 5.7)	2.79 dd (12.2, 5.4)	2.79 dd (12.1, 5.5)
11α	1.54 m	1.52 m	1.54 m	1.55 m	1.54 m	1.56 m	1.50 m	1.49 m
β	1.75 m	1.74 m	1.80 m	1.76 m	1.80 m	1.79 m	1.75 m	1.75 m
12α	1.41 td (12.7, 4.1)	1.40 td (12.8, 4.3)	1.50 m	1.43 td (13.7, 4.6)	1.47 m	1.43 m	1.42 m	1.42 m
β	1.63 m	1.69 m	1.74 m	1.73 m	1.74 m	1.74 m	2.12 m	2.13 m
14	2.26 br t (8.9)	2.25 br t (8.6)	2.26 br t (9.0)	2.22 br t (8.8)	2.25 br t (8.6)	2.22 br t (9.0)	2.10 m	2.10 m
15α	1.62 m	1.61 m	1.56 m	1.57 m	1.56 m	1.57 m	1.38 m	1.38 m
β	1.62 m	1.61 m	1.52 m	1.52 m	1.49 m	1.53 m	1.46 m	1.47 m
16α	1.66 m	1.69 m	1.83 m	1.82 m	1.83 m	1.82 m	1.56 m	1.58 m
β	1.84 m	1.85 m	1.65 m	1.68 m	1.67 m	1.68 m	1.66 m	1.66 m
17	2.89 br t (9.8)	2.86 t (9.5)	2.24 br t (9.5)	2.27 br t (9.7)	2.23 br t (9.1)	2.27 br t (9.6)	1.66 m	1.66 m
18α	2.48 br s	2.48 br s	2.48 br s	2.48 br s	2.48 br s	2.48 br s	2.47 br s	2.47 br s
β	2.48 br s	2.48 br s	2.48 br s	2.48 br s	2.48 br s	2.48 br s	2.47 br s	2.47 br s
19	0.64 s	0.63 s	0.50 s	0.53 s	0.49 s	0.53 s	0.70 s	0.70 s
21	1.70 s	1.70 d (1.1)	1.68 s	1.65 s	1.65 s	1.64 s	1.20 s	1.20 s
22	5.33 d (9.2)	5.33 dd (9.3, 1.1)	5.17 d (8.7)	5.15 d (8.8)	5.22 d (8.2)	5.22 d (8.4)	5.62 dd (15.6, 0.9)	5.64 dd (15.6, 1.1)
23	3.99 ddd (9.2, 6.0, 4.6)	4.11 ddd (9.3, 4.9, 4.1)	3.96 ddd (8.7, 6.6, 4.3)	3.95 ddd (8.8, 6.6, 4.3)	4.09 ddd (8.2, 4.7, 4.0)	4.06 ddd (8.4, 4.7, 3.9)	5.49 dd (15.6, 5.9)	5.49 dd (15.6, 5.3)
24	3.41 m	3.47 m	3.41 m	3.40 m	3.49 m	3.50 m	4.09 m	4.09 m
25	1.00 d (6.3)	1.01 d (6.3)	0.95 d (6.3)	0.96 d (6.3)	0.96 d (6.3)	0.98 d (6.3)	1.08 d (6.4)	1.08 d (6.4)
3-OH	4.62 d (4.3)	4.62 d (4.3)	4.59 d (4.4)	4.63 d (4.3)	4.62 d (4.3)	4.60 d (4.2)	4.61 d (4.3)	4.61 d (4.3)
20-OH	-	-	-	-	-	-	4.23 s	4.22 s
23-OH	4.30 d (4.6)	4.28 d (4.9)	4.46 d (4.3)	4.48 d (4.3)	4.40 d (4.7)	4.35 d (4.7)	-	-
24-OH	4.31 d (4.7)	4.31 d (4.9)	4.33 d (4.1)	4.39 d (4.1)	4.29 d (5.0)	4.27 d (4.6)	4.57 d (4.4)	4.57 d (4.6)

^a^ Chemical shifts were recorded in δ_H_ values using the solvent DMSO-*d*_6_ signal δ_H_ 2.50 as reference; ^b^ Signals of two new compounds **1** and **2** were assigned on the basis of DEPT, ^1^H-^1^H COSY, HMQC, HMBC, NOESY, and 1D difference NOE experiments, respectively.

**Table 2:** 100 MHz ^1^^3^C NMR Data of **1**–**11** in DMSO-*d*_6_ and **12**–**14** in CDCl_3_^a^.

Position	1 ^b^	2 ^b^	3 ^b,c^	4	5	6	7	8 *^c^*	9	10	11	12	13	14
1	122.1	122.1	122.1	122.1	122.1	122.1	122.1	122.1	122.1	122.0	121.9	122.0	122.0	122.1
2	36.0	36.0	36.0	35.9	36.0	36.0	36.0	35.9	36.0	36.0	35.9	35.8	35.7	35.8
3	63.1	63.1	63.1	63.1	63.1	63.1	63.1	63.1	63.1	63.1	63.1	64.6	64.6	64.7
4	41.4	41.4	41.4	41.4	41.4	41.4	41.4	41.4	41.4	41.4	41.3	41.8	41.7	41.8
5	48.1	48.1	48.1	48.1	48.1	48.1	48.1	48.1	48.1	48.1	48.1	48.7	48.7	48.7
6	204.1	204.1	204.1	204.1	204.1	204.1	204.1	204.1	204.1	204.1	204.1	205.5	205.5	205.4
7	124.5	124.5	124.5	124.3	124.3	124.2	124.3	124.3	124.3	124.6	124.56	125.1	125.1	125.3
8	157.0	157.1	157.0	156.9	157.0	157.0	157.0	156.9	156.93/156.90	157.1	157.1	158.0	158.0	157.4
9	53.2	53.3	53.3	53.1	53.3	53.2	53.3	53.2	53.2	53.2	53.2	54.2	54.2	54.1
10	145.5	145.5	145.5	145.5	145.5	145.6	145.6	145.5	145.5	145.7	145.7	146.1	146.0	146.0
11	27.2	27.3	27.3	27.5	27.4	27.5	27.4	27.4	27.5	27.5	27.5	27.8	27.8	27.8
12	36.7	36.6	36.4	37.3	36.9	37.2	36.9	37.07/37.13	37.08/37.13	38.9	38.9	39.5	39.5	39.4
13	47.8	47.8	47.73/47.66	46.6	46.9	46.6	46.9	46.76/46.84	46.74/46.76	45.9	45.9	46.5	46.4	46.5
14	54.2	54.3	54.2	54.4	54.3	54.5	54.3	54.3	54.4	55.3	55.2	56.1	56.0	55.9
15	22.6	22.6	22.7	22.2	22.4	22.3	22.4	22.3	22.34/22.38	22.1	22.1	22.7	22.6	22.9
16	24.1	24.0	24.1/24.0	23.7	23.7	23.8	23.7	23.8/23.7	23.82/23.80	22.3	22.3	22.6	22.6	21.4
17	50.9	51.0	51.2	58.6	58.9	58.6	58.9	58.9	58.87/58.92	60.1	60.1	60.3	60.4	55.4
18	27.2	27.2	27.3	27.2	27.2	27.2	27.2	27.2	27.2	27.2	27.1	27.7	27.7	27.7
19	14.8	14.7	14.7	13.3	13.5	13.3	13.4	13.53/13.49	13.48/13.44	14.3	14.3	14.9	15.0	14.3
20	136.7	136.6	140.1/140.0	135.8	135.8	134.9	135.0	139.5	138.78/138.67	73.3	73.3	79.5	79.7	76.9
21	22.2	22.3	22.2/22.0	18.9	17.3	18.8	17.3	18.2/18.5	18.04/18.27	29.0	28.9	21.8	21.8	20.8
22	130.7	130.8	126.9	127.1	128.2	127.3	128.2	124.3/124.1	124.4/124.3	136.2	136.0	134.5	134.5	77.6
23	70.4	70.3	79.9/79.7	72.2	72.0	71.6	71.5	81.86/81.94	81.3/81.4	130.8	130.8	134.2	134.4	129.4
24	70.1	69.8	68.6	70.3	70.3	69.8	69.8	68.7	68.32/68.38	66.7	66.3	68.8	68.8	130.4
25	19.8	18.5	18.8/19.9	19.1	18.9	18.3	18.5	18.9	18.9/18.8	24.1	24.0	23.8	23.7	18.2
23-OCH_3_	-	-	55.0/54.9	-	-	-	-	55.44/55.39	55.5	-	-	49.8	49.9	-

^a^ Chemical shifts were recorded in δ_C_ values using the solvent signals (DMSO-*d*_6_: δ_C_ 39.52 for **1**–**11**; CDCl_3_: δ_C_ 77.16 for **12**–**14**) as references; ^b^ Signal assignments for three new compounds **1**–**3** were based on the results of DEPT, ^1^H-^1^H COSY, HMQC, HMBC, NOESY, and 1D difference NOE experiments; ^c^ The 23-*O*-methylantineocyclocitrinol (**3**) was obtained as a mixture of the 23-*O*-methyl derivatives of **1** (24*R*) and **2** (24*S*) in an approximate 4:1 ratio of the 24*R* and 24*S* forms. Signals of the C-20, 21, 23 and 25 carbons appeared as a pair of separated signals in the ^13^C NMR spectrum and their data are given as the former/latter signals for the 24*R* and 24*S* forms, respectively.

**Figure 3:**
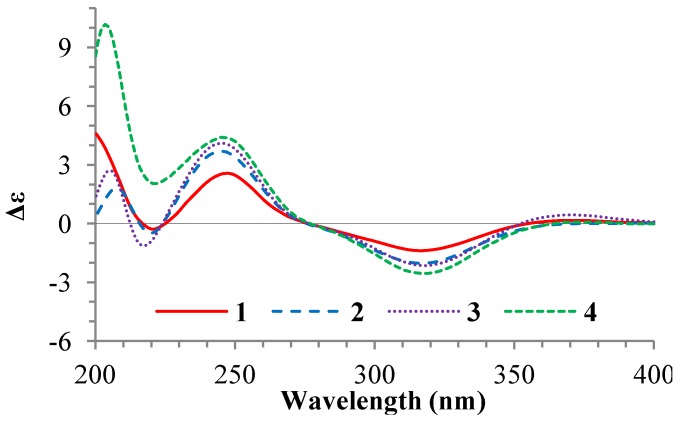
CD spectra of **1**–**4** in MeOH solution.

The NOEs between pairs of the protons, H_3_-21/H_3_-19, H_3_-19/Hα-18 and H-9/H-14, indicated the orientations of C-20, C-19 and C-18 on the same side of the A–D ring system and H-9, H-14 and H-17 on the other side of the ring system in **1** and **2**. Inspection of the HGS molecular modeling indicated that H_3_-19 and Hα-18 are in an interatomic distance to show NOE interactions, while Hβ-18 points away from H_3_-19. So, the NOE interaction from one of the H_2_-18 protons with the H_3_-19 protons could be ascribed to Hα-18, though the signals from Hα-18 and Hβ-18 are overlapped. Further, the ^1^H and ^13^C NMR signals of the A/B ring moieties in **1** and **2** are almost identical with the signals from the same moieties in **4**–**7** (Table 1 and Table 2), indicating the 3β-OH in **1** and **2** are the same as **4**–**7**. Absolute configurations of the A–D ring moieties in **1** and **2** were determined to be the same as **4** by their circular dichroism (CD) spectra that closely resembled the CD spectrum of **4** as shown in Figure 3. The NOEs between H_3_-21 and H-22 both for **1** and **2** (Figure 2) established the *Z*-configurations of the 20,22-double bonds in **1** and **2**. This just differed from the *E*-configurations of the 20,22-double bonds in **4**–**7**, which were determined by the NOEs between H_3_-21 and H-23 [1].

**Figure 4:**
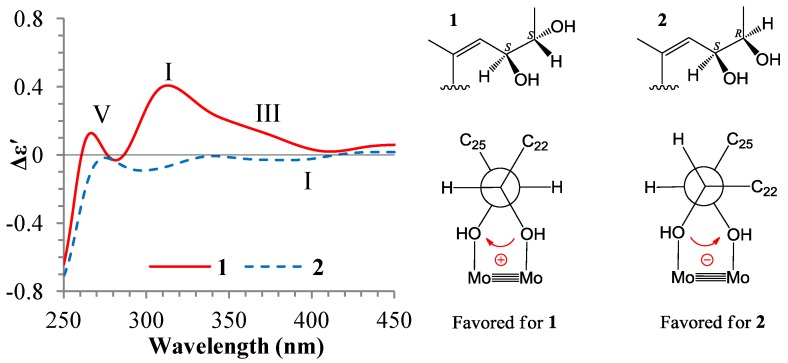
Induced circular dichroism (ICD) spectra from the Mo_2_-complexes of **1** and **2** in DMSO.

The ^1^H and ^13^C NMR data of **1** and **2** in DMSO-*d*_6_ (Table 1 and Table 2) are almost identical except for the signals from CH-23 and CH-24. These NMR data indicated that **1** and **2** should be stereoisomers at C-23 and/or C-24. Further, the couplings of H-23/H-24 (6.0 Hz for **1** and 4.1 Hz for **2**) indicated that **1** was a 23,24-*threo*-isomer and **2** was a 23,24-*erythro*-isomer. The H-23/H-24 couplings were larger than 6 Hz in *threo*-isomers but smaller than 5 Hz in *erythro*-isomers [1,21]. Further support was also from the resonances of H-23 and H-24 in **1** which was upfield than **2**. The same protons in the *threo*-isomers had chemical shifts upfield than in the *erythro*-isomers [1]. The same configurations at C-23 in **1** and **2** were evident because the chemical shifts of C-21 and C-23 in **1** and **2** were consistent [1]. On the other hand, the 23,24-*threo*-diol steroids **4** and **5** gave their C-25 signals at around δ_C_ 19.0, but the 23,24-*erythro*-diol steroids **6** and **7** at around δ_C_ 18.4 (Table 2). By comparison of the ^13^C signals of **4**–**7** (Table 2) between *threo*/*erythro* pairs of C-24 epimers, it appears that the difference of C-25 δ values (∆δ_C25_) between **4**/**6** (∆δ_C25_ = 19.1 − 18.3 = 0.8) and **5**/**7** (Δδ_C25_ = 18.8 − 18.5 = 0.4) are significant and diagnostic to discriminate the two pairs of the C-24 epimers. Similar calculations for the *threo*/*erythro* pairs of C-23 epimers gave the same Δδ_C25_ values for **4**/**7** (Δδ_C25_ = 19.1 − 18.5 = 0.6) and **5**/**6** (Δδ_C25_ = 18.9 − 18.3 = 0.6). The Δδ_C25_ value from **1**/**2** (Δδ_C25_ = 19.8 − 18.5 = 1.3), similar to **4**/**6**, indicated the absolute configurations 23*S*,24*S*/23*S*,24*R* for **1**/**2**, the same as **4**/**6**. Direct comparison of the C-25 chemical shift of the 23,24-*threo*-diol **1** (23*S*,24*S*; δ_C25_ 19.8) with those of the 23,24-*threo*-diols **4** (23*S*,24*S*; δ_C25_ 19.1) and **5** (23*R*,24*R*; δ_C25_ 18.9) or of the 23,24-*erythro*-diol **2** (23*S*,24*R*; δ_C25_ 18.5) with 23,24-*erythro*-diols **6** (23*S*,24*R*; δ_C25_ 18.3) and **7** (23*R*,24*S*; δ_C25_ 18.5) also supported the same conclusion, the 23*S*,24*S* for **1** and the 23*S*,24*R* for **2**. The absolute configurations 23*S*,24*S* for **1** and 23*S*,24*R* for **2** could be also assigned by the dimolybdenum induced CD (ICD) analysis. In the ICD measurements by the Snatzke’s method using dimolybdenum tetraacetate [Mo_2_(OAc)_4_] [22,23], the Mo_2_-complex of **1** in DMSO gave positive Cotton effects around 310 nm (band IV) and near 400 nm (band II), while the Mo_2_-complex of **2** gave negative bands II and IV (Figure 4). By the helicity rule of the Snatzke’s method, the sign of the torsional angle determines the signs of particular Cotton effects [22,23], that is, the negative signs of the bands II and IV were determined by the counterclockwise O–C–C–O torsional angle (negative sign of torsional angle) in the favored conformation of chiral Mo_2_-complex, and contrarily, the positive signs of the bands II and IV by the clockwise O–C–C–O torsional angle (the positive sign of the torsional angle). Since the favored conformations in the Mo_2_-complexes of **1** and **2** were preferred as shown in Figure 4 by the ICD investigations on **4**–**7** (see Section 2.3), the absolute configurations of **1** and **2** at C-23 and C-24 could be assigned to be 23*S*,24*S* for **1** and 23*S*,24*R* for **2** on the basis of their positive and negative signs of the bands II and IV (Figure 4), respectively.

23-*O*-Methylantineocyclocitrinol (**3**), a white amorphous powder (from MeOH), 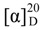 +77.4 (*c* 0.38, MeOH), was assigned the molecular formula C_2__6_H_3__8_O_4_ by HRESIMS (measured 415.2850 [M + H]^+^, calculated for C_2__6_H_3__9_O_4_ [M + H]^+^ 415.2848). The maximal UV absorption at 243.8 nm (log ε 4.10) in MeOH and the strong IR absorption at 1651 cm^−1^ indicated the presence of a conjugated carbonyl group in **3** [1]. The IR spectrum also showed the absorptions due to OH (3374 cm^−1^) and CH_3_/CH_2_ (2946, 2910, 2878 and 1378 cm^−1^) groups. The ^1^H and ^13^C NMR spectra of **3** in DMSO-*d*_6_ gave ^1^H and ^13^C signals (Table 2 and Table 3) resembled those of **1** and **2** (Table 1 and Table 2), except additional ^1^H and ^13^C signals from a methoxy group were detected along with disappearance of a hydroxyl signal in the ^1^H NMR spectrum. This indicated that **3** was a methoxylated derivative of **1** and **2**. The MS ion fragments detected at *m*/*z* 383 [M − CH_3_OH + H]^+^ in the positive ESIMS and at *m*/*z* 381 [M − CH_3_OH − H]^−^ in the negative ESIMS also supported the presence of a methoxy group in **3**. In addition, several ^1^H and ^13^C NMR signals of **3** appeared as pairs in an approximate 4:1 ratio, and significant changes were mainly observed on the signals from side chain moieties in **1**–**3** (Table 1, Table 2 and Table 3). These observations revealed that the methoxy group in **3** should be in the side chain and **3** was a mixture. Detailed analysis of the ^1^H and ^13^C NMR spectra with the aide of DEPT, 1D difference NOE, ^1^H-^1^H COSY, HMQC, HMBC and NOESY techniques (Table S3 in the Supplementary Information) established the structure of **3** as shown in Figure 1. The methoxy group in **3** was located at C-23 by the HMBCs shown in Figure 2. The NOEs between H_3_-21 and H-22 demonstrated the *Z*-configuration of the 20,22-double bond in **3**. The relative stereochemistry of the ring system in **3** was determined to be the same as **1** and **2** on the basis of the NOEs between pairs of the protons, H_3_-21/H_3_-19, H_3_-19/Hα-18 and H-9/H-14, and the almost identical ^1^H and ^13^C signals of the A/B ring moieties in **1**–**3** (Table 1, Table 2 and Table 3). The absolute configuration of the A–D ring system in **3** was established to be the same as **1**–**2** and **4** by the closely resembled CD spectra of **1**–**4** as shown in Figure 3. The couplings of H-23 and H-24 in **3**, 5.9 Hz for major isomer and 4.0 Hz for minor isomer, indicated that the major isomer in **3** was the 23,24-*erythro* and the minor isomer was the 23,24-*thero* [1,21]. This was supported also by the downfield resonances of H-23 and H-24 in the major *erythro* isomer than in the minor *thero* isomer (see Table 3) [1]. Further, the chemical shifts of C-21 and C-23 in the major and minor isomers were consistent (Table 2) and indicated the same configuration at C-23 [1]. Thus, **3** was the 4:1 mixture of 23-methoxylated **2** and **1**.

### 2.3. Absolute Configuration Assignment of vic-Diols in **4**–**7** and **14** by ICD Analysis

Absolute configuration assignments of the acyclic and thus flexible 1,2-diols have now been able to use the dimolybdenum tetracetate-induced CD method for various kinds of 1,2-diol groups [23,24]. The restriction of conformational flexibility of the acyclic 1,2-diols by ligation to the Mo_2_-core in the Mo_2_-diol complex has enabled use of the method to these flexible 1,2-diols. The existence of a single favored conformer with antiperiplanar orientations of both O^1^–C–C–R and O^2^–C–C–R' units in the Mo_2_-diol complex of *threo*-1,2-diols enabled straightforward use of the empirical “helicity rule” to assign their absolute configurations: The sign of the torsional O^1^–C–C–O^2^ angle determines the signs of Cotton effects (bands II and IV) in the 300–400 nm region [22,24]. However, in the case of the Mo_2_-diol complex of *erythro*-1,2-diols, the O^1^–C–C–R and O^2^–C–C–R' units cannot simultaneously adopt an antiperiplanar orientation in a conformation, and two possible arrangements of the O^1^–C–C–R and O^2^–C–C–R' units in an antiperiplanar orientation result in two possible *gauche* conformations that lead to opposite signs of the decisive CD bands for the same absolute configuration. Recent studies on the Mo_2_-induced CD of the *erythro*-1,2-diols have shown that the preferred conformation of the *erythro*-1,2-diols after ligation to the Mo_2_-core was a *gauche* with largest one of the R or R' group in an antiperipalanr orientation of O^1(or 2)^–C–C–R(or R') [23]. This thus enabled extending the empirical “helicity rule” to the *erythro*-1,2-diols. In other words, the largest R or R' group determines the preferred conformation in the formed Mo_2_-complex and does itself points away from the rest of the complex to avoid the steric interaction with the remaining acetate ligands in the stock complex [23]. As a matter of fact, the steric interaction of bulky group plays a primary role and dominates the formation of preferred conformation. Therefore, discrimination of the stronger steric interaction from R and R' in *erythro*-1,2-diols is most important for the conformational analysis prior to the use of the empirical “helicity rule”. However, the stronger steric interaction from two groups was not always easily discriminated only according to the bulkiness of two groups. Sometimes, the difference in the bulkiness of the two groups is too small and additional lager group also affected the steric interaction of the two groups. The same is true of the case of *erythro*-23,24-diols **2**, **5** and **6**. In this case, the C-25 methyl group is spatially bulkier to the 23,24-diols than the C-22 *sp*^2^ methine, however, a larger A–D ring moiety linked to C-22 has made it difficult to estimate which is stronger in steric interaction. This has brought about doubts in the application of the “helicity rule” to these *erythro*-23,24-diols. This problem was solved by following ICD investigations on **4**–**7** and **14** using the Mo_2_(OAc)_4_-induced CD method.

**Figure 5:**
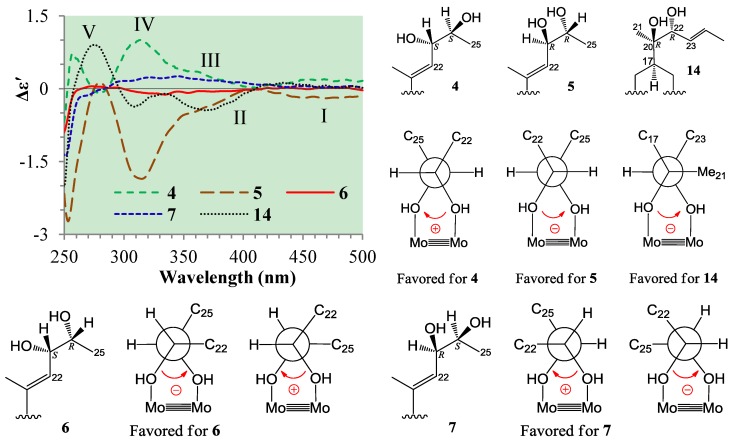
ICD spectra and favored conformations for **4**–**7** and **14**.

In the ICD spectra, the *threo*-23,24-diol **4** (23*S*,24*S*) gave positive Cotton effects around 310 (band IV) and 375 nm (band III) and near 400 nm (band II), while the *threo*-23,24-diol **5** (23*R*,24*R*) and the *threo*-20,22-diol **14** (20*R*,22*R*) both gave negative Cotton effects of the bands IV, III, and II (Figure 5). Since these *threo*-*vic*-diols form a single preferred conformation with both antiperiplanar orientations of the O^1^–C–C–R and O^2^–C–C–R' units in Mo_2_-complexes (Figure 5), their absolute configurations could be assigned straightforwardly by the signs of their Cotton effects (bands II/IV) according to the signs of torsional O^1^–C–C–O^2^ angles in their preferred conformations [22,23]. The lower intensity of the ICD from the Mo_2_-complex of **14** may probably be caused by strong steric effects of three bulky groups around the 20,22-diols, which decreased the Mo_2_-complex formation.

On the other hand, the *erythro*-23,24-diol **6** (23*S*,24*R*) showed very weak but significant negative Cotton effects in the band II–IV region, while the *erythro*-23,24-diol **7** (23*R*,24*S*) gave positive Cotton effects in the same region (Figure 5). As mentioned, there are two possible conformations with an antiperiplanar orientation of O^1(or 2)^–C–C–R(or R') in the Mo_2_-complex of the *erythro*-*vic*-diols [23]. In the Mo_2_-complexes of **6** and **7**, both two conformations existed: One with antiperiplanar orientation of O–C^23^–C^24^–Me^25^ and another one with antiperiplanar orientation of O–C^24^–C^23^–CH^22^ (Figure 5). The sign of the torsional O–C^23^–C^24^–O angle in the former conformation was negative for **6** and positive for **7**, while the sign of the same torsional angle in the latter conformation was positive for **6** and negative for **7** (Figure 5). The steric interaction of the C-25 methyl group with the 23,24-diols seems likely to be slightly stronger than the C-22 *sp*^2^ methine group, though the C-22 carbon linked with a bulky A–D ring system, the free rotation of C^22^–C^23^ bond may enable the ring system points away from the Mo_2_-core. This thus caused the formation of a little more amounts of the antiperiplanar O–C^23^–C^24^–Me^25^ conformations in Mo_2_-complexes of **6** and **7**, which gave negative and positive signs of the bands II–IV, respectively. However, the opposite contributions of the antiperiplanar O–C^24^–C^23^–CH^22^ conformations have somewhat counteracted the effects from the antiperiplanar O–C^23^–C^24^–Me^25^ conformations, resulting in the lower intensities of the ICDs of **6** and **7**. Nevertheless, the absolute configurations of *erythro*-23,24-diols in **6** (23*S*,24*R*) and **7** (23*R*,24*S*) could be assigned by the signs of their band II and IV Cotton effects according to the signs of the torsional O–C^23^–C^24^–O angles in their antiperiplanar O–C^23^–C^24^–Me^25^ conformations (Figure 5). Similar to the ICD of *erythro*-23,24-diol **6** (23*S*,24*R*) (Figure 5), the ICD of **2** (23*S*,24*R*) also gave weak but significant negative Cotton effects in the band II–IV region (Figure 4). The signs and lower intensities of these Cotton effects from **2** could be explained in terms of two possible conformations as shown for **6**. These results indicated that the steric effect of the C-25 methyl group dominates the formation of preferred conformation in the Mo_2_-complex of *erythro*-23,24-diol C25 steroids with bicyclo[4.4.1]A/B rings, and the preferred conformation is the *gauche* with the antiperiplanar O–C^23^–C^24^–Me^25^ orientation.

**Table 3:** 400 MHz ^1^H NMR Data of **3**, **8** and **9** in DMSO-*d*_6_ and **12**–**14** in CDCl_3_ (δ_H_, *J* in Hz) ^a^.

Position	3 ^b^	8	9	12	13	14
1	5.55 dd (8.1, 6.4)	5.56 dd (8.3, 6.3)	5.55 dd (8.1, 6.5)	5.55 dd (8.2, 6.2)	5.54 m	5.56 dd (8.4, 6.3)
2α	2.07 m	2.07 m	2.07 m	2.23 m	2.22 m	2.24 ddt (13.4, 8.4, 2.2)
β	2.34 ddd (13.3, 11.4, 6.4)	2.34 m	2.34 m	2.47 ddd (13.4, 11.3, 6.2)	2.46 ddd (13.1, 11.4, 6.0)	2.48 ddd (13.4, 11.3, 6.3)
3	3.11 m	3.12 m	3.12 m	3.47 m	3.46 m	3.50 m
4α	2.63 br d (13.0)	2.63 br d (13.2)	2.63 br d (13.1)	2.87 br d (12.8)	2.87 br d (12.8)	2.89 br d (13.0)
β	1.52 m	1.53 m	1.52 m	1.68 m	1.67 m	1.66 m
5	2.69 m	2.68 m	2.68 m	2.72 m	2.71 m	2.74 m
7	5.43 s	5.43 s	5.43 s	5.55 br s	5.54 br s	5.58 br s
9	2.84 dd (11.2, 6.0)	2.85 dd (11.5, 5.5)	2.85 dd (11.5, 5.4)	2.74 dd (12.5, 5.8)	2.73 dd (12.6, 5.8)	2.76 dd (12.0, 5.8)
11α	1.50 m	1.54 m	1.54 m	1.56 m	1.55 m	1.59 m
β	1.77 m	1.79 m	1.78 m	1.65 m	1.64 m	1.86 m
12α	1.41 td (12.4, 4.0)	1.49 m	1.49 m	1.43 td (13.0, 4.8)	1.42 m	1.47 td (13.1, 4.4)
β	1.68 m	1.75 m	1.75 m	2.18 m	2.17 m	2.16 m
14	2.29 br t (7.9)	2.26 br t (9.2)	2.25 br t (9.2)	2.06 ddd (12.0, 6.4, 1.5)	2.05 ddd (12.4, 6.6, 1.4)	2.08 ddd (12.0, 6.1, 1.6)
15α	1.62 m	1.57 m	1.57 m	1.46 m	1.46 m	1.62 m
β	1.62 m	1.53 m	1.52 m	1.58 m	1.57 m	1.54 m
16α	1.71 m	1.84 m	1.83 m	1.74 m	1.71 m	1.91 m
β	1.90 m	1.70 m	1.70 m	1.78 m	1.77 m	1.67 m
17	2.84 m (covered by H-9 signals)	2.33 m	2.33 m	1.80 m	1.79 m	1.68 m
18α	2.48 br s	2.48 br s	2.48 br s	2.54 br s	2.53 m	2.54 d (13.6)
β	2.48 br s	2.48 br s	2.48 br s	2.55 m	2.54 m	2.59 dd (13.6, 6.0)
19	0.65 s/0.67 s	0.55 s/0.53 s	0.54 s/0.53 s	0.73 s	0.71 s	0.86 s
21	1.77 s	1.71 s	1.68 s	1.28 s	1.27 s	1.25 s
22	5.17 dd (10.2, 1.0)/5.14 dd (10.2, 1.0)	5.02 d (9.4)	5.12 d (8.9)	5.66 d (16.0)	5.67 d (16.0)	3.90 dd (8.3, 0.6)
23	3.84 dd (10.0, 4.0)/3.74 dd (10.0, 5.9)	3.71 dd (9.4, 6.7)	3.77 dd (8.9, 4.0)/3.79 dd (8.9, 3.8)	5.55 dd (16.0, 6.0)	5.54 dd (16.0, 6.1)	5.41 ddq (15.3, 8.2, 1.6)
24	3.56 m/3.50 m	3.53 m	3.60 m	4.34 m	4.33 m	5.77 dqd (15.3, 6.5, 0.6)
25	1.03 d (6.3)/1.01 d (6.3)	0.95 d (6.4)/0.94 d (6.3)	0.99 d (6.3)/0.97d (6.1)	1.28 d (6.3)	1.27 d (6.3)	1.73 dd (6.5, 1.6)
3-OH	4.63 d (4.3)	4.62 d (4.2)	4.63 d (3.8)	Not detected	Not detected	Not detected
20-OH	-	-	-	-	-	Not detected
24-OH	4.47 d (4.9) /4.44 d (4.7)	4.45 d (3.9)/4.44 d (3.8)	4.44 d (3.6)	Not detected	Not detected	Not detected
20-OCH_3_	-	-	-	3.11 s	3.12 d (0.7)	-
23-OCH_3_	3.11 s	3.147 s/3.152 s	3.15 s/3.16 s	-	-	-

^a^ Chemical shifts were recorded in δ_H_ values using the solvent signals (DMSO-*d*_6_: δ_H_ 2.50 for **3**, **8** and **9**; CDCl_3_: δ_H_ 7.26 for **12**–**14**) as references; ^b^ The 23-*O*-methylantineocyclocitrinol (**3**) was obtained as a mixture of the 23-*O*-methyl derivatives of **1** (24*R*) and **2** (24*S*) in an approximate 4:1 ratio of the 24*R* and 24*S* forms. Signals of the H-19, H-22 to H-25 and HO-24 protons appeared as a pair of separated signals in the ^1^H NMR spectrum and their data are given as the former/latter signals for the 24*R* and 24*S* forms, respectively. The signal assignments were based on the results of DEPT, ^1^H-^1^H COSY, HMQC, HMBC, NOESY, and 1D difference NOE experiments.

### 2.4. Inhibitory Effects of **1**–**14** on Several Human Cancer Cell Lines

Antitumor activities of **1***–***14** were preliminarily tested by the MTT method using the human cancer K562, HL-60, HeLa, and BGC-823 cell lines. All of **1***–***14** were assayed for their effects on each of the four human cancer cell lines. Compounds **1***–***14** inhibited the cell lines to varying extents and their IR% values on the cells at 100 µg/mL are given below. **1**: 17.1% (K562); 34.7% (HL-60); **2**: 27.5% (K562), 27.7% (HL-60), 13.3% (HeLa); **3**: 23.9% (K562), 33.8% (HL-60), 13.9% (HeLa); **4**: 23.4% (K562), 26.9% (HL-60), 16.8% (HeLa); **5**: 19.8% (K562), 34.7% (HL-60), 16.1% (HeLa), 21.9% (BGC-823); **6**: 17.2% (K562), 21.0% (HL-60), 15.5% (HeLa); **7**: 15.2% (K562), 22.2% (HL-60), 25.7% (HeLa); **8**: 19.3% (K562), 25.4% (HL-60); **9**: 10.1% (HL-60), 11.7% (HeLa); **10**: 31.4% (K562), 19.8% (HL-60); **11**: 27.9% (K562); 21.0% (HL-60), 19.6% (HeLa); **12**: 37.3% (K562); 40.4% (HL-60), 63.5% (HeLa), 28.7% (BGC-823); **13**: 11.9% (K562), 24.1% (HL-60); **14**: 20.6% (K562); 24.0% (HL-60), 14.7% (HeLa). The positive control docetaxol inhibited these cell lines with the IR% values of 42.90% (K562), 46.90% (HL-60), 41.70% (HeLa), and 44.10% (BGC-823) at 100 µg/mL.

### 2.5. Discussions

By tracing newly produced metabolites in the mutant AD-1-2 extract, we have isolated 14 C25 steroids with unusual bicyclo[4.4.1]A/B rings, including three new (**1**–**3**) and 11 known ones (**4**–**14**). HPLC-PDAD-UV and HPLC-ESI-MS analyses indicated that the original G59 strain did not produce these metabolites and the production of **1**–**14** in the mutant AD-1-2 extract was caused by the activation of silent metabolites in original G59 strain by DES mutagenesis. Compounds **1**–**3** provide the first examples of the C25 bicyclo[4.4.1]A/B ring steroids with the *Z*-configuration of 20,22-double bond. These results provided additional examples for the successful application of the chemical mutagenesis strategy using DES to discover new compounds by activating silent metabolites in fungal isolates [17,19], and supported also the effectiveness and usefulness of the new strategy [17].

Relating to the absolute configuration assignments of new *erythro*-23,24-diol **2** and *threo*-23,24-diol **1**, we investigated the ICDs of the known *erythro*-23,24-diols **5** and **6** and *threo*-*vic*-diols **4**, **7** and **14** by the Mo_2_-induced CD method [23,24]. The present results indicated that the empirical “helicity rule” could be straightforwardly applied to the *threo*-*vic*-diols **1**, **4**, **7** and **14** to unambiguously assign their absolute configurations as reported [23]. Although intensities of the band II and IV Cotton effects were rather lower in the ICD of **14**, it could be explained by the strong steric effects of three bulky groups around the *vic*-diol group, leading to the decrease of the Mo_2_-complex formation. On the other hand, the ICD investigations on the *erythro*-*vic*-diols **2**, **5** and **6** showed that two possible conformations with an antiperiplanar O–C^23^–C^24^–Me^25^ or O–C^24^–C^23^–CH^22^ orientation both exist in their Mo_2_-complexes, and counteract each other the Cotton effects from the same absolute configuration *vic*-diol molecules. Fortunately, a little larger amount of the preferred O–C^23^–C–Me^25^ conformations formed in their Mo_2_-complexes and gave weak but significant Cotton effects in the decisive band II and IV regions. Thus, the absolute configurations of these *vic*-diols could be assigned by the “helicity rule” according to the signs of torsional O–C^23^–C^24^–O angles in their antiperiplanar O–C^23^–C^24^–Me^25^ conformations (Figure 5). The formation of the extra amounts of the antiperiplanar O–C^23^–C^24^–Me^25^ conformations could be explained by the difference in the steric effects of the C-25 methyl and the *sp*^2^ C-22 methine groups. The *sp*^3^ C-25 methyl group is spatially bulkier to the 23,24-diols than the *sp*^2^ C-22 methine group, and thus showed slightly stronger steric interaction with the diols than the C-22 methine group. Although the *sp*^2^ C-22 methine carbon linked with a bulky A–D ring system, the free rotation of the C^22^–C^23^ bond enabled the ring system points away from the Mo_2_-core. Thus, the slightly stronger steric effect of the C-25 methyl group dominated the formation of the decisive, extra amounts of the preferred antiperiplanar O–C^23^–C^24^–Me^25^ conformation in the Mo_2_-complex. These results suggested that the antiperiplanar O–C^23^–C^24^–Me^25^ *gauche* conformation is also the preferred conformation in the free *erythro*-23,24-diols in the bicyclo[4.4.1]A/B ring C25 steroids.

In present MTT assay, **1**, **3**, **5**, **10** and **12** inhibited the K562, HL-60, and/or HeLa cells with the IR% values over 31.4% at 100 µg/mL. However, as a whole, **1**–**14** showed very weak effects on the tested four human cancer cell lines. This coincides with the previous report on the isocyclocitrinol A and 22-acetylisocyclocitrinol A, which recorded that both compounds were inactive against murine and human tumor cell lines but showed weak antibacterial activity on *Staphylococcus epidermidis* and *Enterococcus durans* [3]. Similar antibacterial activity was also recorded for cyclocitrinol (**11**) in the literature [2] (the structure reported for the cyclocitrinol in this paper [2] was later revised to **11** [3]). On the other hand, **4**, **7**, **8**, **10**, **11** have been shown to induce the receptor-dependent production of cAMP in GPR12-transfected CHO cells at 10 µM [1]. However, the 16 known C25 steroids in this class of compounds have not been identified for their more promising bioactivities so far. Nevertheless, the very unusual skeletal structures of these steroids warrant further evaluation of biological activity.

## 3. Experimental Section

### 3.1. General Experimental

Melting point was measured on a Beijing Tiandiyu X-4 exact micro melting point apparatus (Tiandiyu Science and Technology Co., Ltd., Beijing, China) and temperatures were not corrected. Optical rotations were measured on an Optical Activity Limited polAAr 3005 spectropolarimeter (Optical Activity Limited, Ramsey, UK). ESIMS was recorded on an Applied Biosystems API 3000 LC-MS spectrometer (AB SCIEX, Framingham, MA, USA) and HRESIMS was measured on an Agilent 6520 Q-TOF LC-MS spectrometer (Agilent Technologies, Santa Clara, CA, USA). UV data were recorded on a GBC Cintra 20 spectrophotometer (GBC, Melbourne, Australia). IR spectra were taken on a Bruker Tensor-27 infrared spectrophotometer (Bruker, Karlsruhe, Germany). CD and ICD data were recorded on a Biologic Science MOS450 CD (Bio-Logic, Pont-de-Claix, France) or a JASCO-815 CD spectropolarimeter (JASCO electric Co., Ltd., Tokyo, Japan). 1D and 2D NMR spectra were all obtained on a JEOL JNM-GX 400 (400 MHz ^1^H and 100 MHz ^13^C NMR) NMR spectrometer (JEOL Ltd., Tokyo, Japan). The chemical shifts of ^1^H and ^13^C NMR signals were recorded in δ values using solvent signals (DMSO-*d*_6_: δ_H_ 2.50/δ_C_ 39.52; CDCl_3_: δ_H_ 7.26/δ_C_ 77.16) as references, respectively.

Precoated silica gel GF_254_ plates (10 cm × 20 cm, 0.25 mm thickness for analytical TLC and 20 cm × 20 cm, 0.5 mm thickness for preparative TLC, Yantai Chemical Industrial Institute, Yantai, China) were used in TLC, and spots were detected under sunlight or UV lights (254 and 365 nm) or by using the 10% sulfuric acid reagent or Vaughan’s reagent (24 g of ammonium molybdate tetrahydrate (NH_4_)_6_Mo_7_O_24_·4H_2_O) and 1 g of ceric sulfate Ce(SO_4_)_2_ dissolved in 500 mL of 10% H_2_SO_4_). Silica gel H (100–200 mesh, Yantai Chemical Industrial Institute, Yantai, China), YMC*GEL^®^ ODS-A-HG (12 nm S-50 μm, YMC Co. Ltd., Kyoto, Japan), and Sephadex™ LH-20 (GE Healthcare, Uppsala, Sweden) were used for column chromatography. HPLC was performed on a Waters HPLC systems equipped with Waters 600 controller, Waters 600 pump, Waters 2414 refractive index detector, Waters 2996 (for analytical HPLC) or 2998 (for semi-preparative or preparative HPLC) photodiode array detector, and Waters Empower™ software (Waters, Milford, MA, USA). The Venusil MP C18 (5 μm, 100 Å, 4.6 × 250 mm; Agela Technologies, Tianjin, China), Capcell Pak C18 (UG 120 Å, 4.6 × 250 mm; Shiseido Co., Ltd., Tokyo, Japan) and reversed-phase chiral Click β-CD (4.6 × 150 mm; Dalian Institute of Chemical Physics, Dalian, China) columns were used in analytical HPLC. The reversed-phase chiral Click β-CD (8 × 150 mm; Dalian Institute of Chemical Physics, Dalian, China) and Capcell Pak C18 (UG120Å, 20 × 250 mm; Shiseido Co., Ltd., Tokyo, Japan) columns were used in semi-preparative and preparative HPLC, respectively.

Human chronic myelogenous leukemia K562 cell line was provided by Prof. Dr. Lili Wang (Beijing Institute of Pharmacology and Toxicology, Beijing, China). Human acute promyelocytic leukemia HL-60, human cervical cancer HeLa, and Human gastric adenocarcinoma BGC-823 cell lines were provide by Prof. Dr. Wenxia Zhou (Beijing Institute of Pharmacology and Toxicology). Fetal bovine serum was purchased from Tianjin Hao Yang Biological Manufacture Co., Ltd. (Tianjin, China). The RPMI-1640 medium was purchased from Gibco (lot No. 1403238) and MTT from Amresco (lot No. 0793). Streptomycin (lot No. 071104) and penicillin (lot No. X1103302) were both purchased from North China Pharmaceutical Group Corporation, China. Docetaxol (DOC, lot No. 20110326) was purchased from Beijing Chimivo Technology Co., Ltd. (Beijing, China).

### 3.2. MTT Assay

The EtOAc extracts, fractions, pure compounds and DOC in MeOH at 10 mg/mL were used in MTT assays. DOC was used as positive control and MeOH was used as blank control.

Exponentially growing K562, HL-60, HeLa and BGC-823 cells were suspended in fresh RPMI-1640 medium containing 10% fetal bovine serum and 100 μg/mL penicillin and streptomycin at the cell density of 5 × 10^4^ cells/mL and seeded into 96-well plates each 200 μL/well. The suspension cells, K562 and HL-60, were incubated at 37 °C for 2 h, whereas the adherent cells, HeLa and BGC-823, were incubated at 37 °C for 12 h. Then, 2 μL of MeOH or DMSO for control and the test sample solutions was added to each well, and the cells were cultured at 37 °C for 24 h. After morphological examination of the cells under an inverted microscope, MTT (20 μL; 5 mg/mL in PBS) was added into each well, incubated at 37 °C for 4 h, and centrifuged at 2000 rpm, 4 °C for 20 min. After removal of the supernatant by aspirating, 150 μL DMSO was added into each well, and shaken for 5 min to dissolve formazan crystals. The optical density (OD) value at 570 nm was read for each well using the VERSAmax-BN03152 plate reader (MD, San Francisco, CA, USA). Each three wells were set for control and test groups, respectively, and the inhibition rate (IR%) was calculated using OD mean values according to the formula, IR% = (OD_control_ − OD_sample_)/OD_control_ × 100%.

### 3.3. Fermentation and EtOAc Extract Preparation

#### 3.3.1. Initial Fungal Strain and its Mutant the **1**–**14** Producing Strain

The initial fungal strain *Penicillium purpurogenum* G59 that was used as control strain in the present study was isolated from a soil sample collected at the tideland of Bohai Bay around Lüjühe in Tanggu district of Tianjin, China, in September 2004 [20]. *P. purpurogenum* G59 was identified by Professor Dr. Liangdong Guo, Institute of Microbiology, Chinese Academy of Sciences, China. This strain has been deposited at the China General Microbiological Culture Collection Center under the accession number CGMCC No.3560. The strain *P. purpurogenum* G59 did not produce any secondary metabolites with antitumor activities in the repeated MTT assays using K562 cells at the high concentrations of 100 and 1000 µg/mL [15,16,17,18,19,20].

The producing strain used for **1**–**14** production in the present study is a bioactive mutant AD-1-2 that was obtained by DES mutagenesis of the strain G59 [18]. Fresh G59 spores in 50% (*v*/*v*) DMSO were treated with 0.5% (*v*/*v*) DES at 4 °C for 1 day, and then single colony isolation on the treated spores, followed by bioassays, afforded the mutant AD-1-2. The EtOAc extract of the mutant AD-1-2 cultures inhibited K562 cells (an IR% of 74.5% at 100 µg/mL), whereas the EtOAc extract of the G59 strain by the fermentation at the same time and same conditions did not inhibit the K562 cells (an IR% of 4.8% at 100 µg/mL) [18]. The mutant AD-1-2 has been deposited at the China General Microbiological Culture Collection Center under the accession number CGMCC No.8634.

#### 3.3.2. Preparation of Spore Suspensions

The mutant AD-1-2 was inoculated onto potato dextrose agar (PDA) plates from a PDA slant stock stored at 4 °C and incubated at 28 °C for 4 days. Fresh spores formed on the PDA plates were harvested and suspended in 60 mL of sterilized, distilled water with several glass beads in a 100 mL cone-shaped flask and scattered well by shaken enough to prepare a crude spore suspension. A 100 μL portion of this crude spore suspension was added into a well of 96-well plates, diluted with water with its OD at 600 nm measured using a VERSAmax-BN03152 plate reader, and the dilution ratio was recorded when the OD value reached 0.35. Then, the remaining whole crude spore suspension was diluted with sterilized, distilled water in the same proportion to obtain a mutant AD-1-2 spore suspension. This mutant spore suspension was used for the producing fermentation in the following experiments.

Similarly, the G59 spore suspension was also prepared in the same manner as mentioned above using fresh spores formed by cultivation of the G59 strain at 28 °C for 3 days on PDA plates. This G59 spore suspension was used as control strain in the following experiments.

#### 3.3.3. Fermentation and Extraction

Aliquot (200 µL) of the mutant AD-1-2 spore suspension was inoculated into 240 cone-shaped 500 mL flasks containing 300 mL of the liquid medium (glucose 2%, maltose 1%, mannitol 2%, glutamic acid 1%, peptone 0.5% and yeast extract 0.3% in distilled water, adjusted to pH 6.0 prior to sterilization) and fermented at 28 °C for 12 days on rotary shakers at 200 rpm to obtain approximate 72 L of the fermentation broth. The whole broth (72 L) was filtered to separate into a filtrate (70 L) and a mycelial cake. The filtrate was extracted three times with same volumes of EtOAc to give an EtOAc extract (13.3 g) that inhibited K562 cells with an IR% value of 47.9% at the 100 µg*/*mL. The mycelial cake was extracted three times each time with 10 L acetone-water (2:1) by ultrasonication for 2 h. The aqueous acetone solution obtained by filtration was evaporated under reduced pressure to remove acetone. Then, the remaining water layer was extracted three times with same volumes of EtOAc to give another EtOAc extract (24.0 g) that inhibited the K562 cells with an IR% value of 59.0% at 100 µg*/*mL. The EtOAc extracts both from the filtrate and mycelia gave same TLC spot profiles and thus were combined to afford total 37.3 g EtOAc extract. This EtOAc extract showed an inhibitory effect on the K562 cells with the IR% value of 58.6% at 100 µg*/*mL, which was used for the isolation of **1**–**14** in the following experiments.

On the other hand, each 200 µL of the G59 spore suspension was inoculated into three cone-shaped 500 mL flasks containing 300 mL of the same liquid medium and fermented at the same time and same conditions to obtain 900 mL of the fermentation broth. The whole broth was extracted as described for mutant AD-1-2 to afford an EtOAc extract (610 mg), which did not show inhibitory effect on the K562 cells (an IR% value of 5.6% at 100 µg*/*mL). This G59 extract was used in the MTT assays and TLC analyses as negative control and also in the HPLC-PDAD-UV and HPLC-ESI-MS analyses for detecting **1**–**14** in the following experiments.

### 3.4. Isolation of Compounds **1**–**14**

As HPLC-PDAD-UV and HPLC-ESI-MS analyses indicated that newly produced C25 steroids exist in the mutant extract (Figures S1 and S2 in the Supplementary Information) and preparative TLC separation of the mutant EtOAc extract gave two C25 steroid fractions B3 and B4 (see Figures S3 and S4 in the Supplementary Information), the following separation of the mutant extract was performed tracing the newly produced C25 steroids using the B3 and B4 fractions as reference standards.

The EtOAc extract (37.2 g) of the mutant AD-1-2 was subjected to vacuum liquid chromatography (VLC) on a silica gel column (silica gel 300 g, bed 7.5 × 20 cm) using the b.p. 60–90 °C petroleum ether → dichloromethane (D)–methanol (M) 100:0 → 0:100 as eluting solvents to obtain six fractions: **Fr-1** (3.0 g, eluted by petroleum ether → DM 100:0), **Fr-2** (4.0 g, eluted by DM 99:1→98:2), **Fr-3** (7.0 g, eluted by DM 98:2 → 96:4), **Fr-4** (6.0 g, eluted by DM 96:4 → 92:8), **Fr-5** (10.0 g, eluted by DM 92:8 → 80:20), and **Fr-6** (5.0 g, eluted by methanol). Among them, **Fr-4** contained the C25 steroids and inhibited the K562 cells with an IR% value of 45.2% at 100 µg*/*mL.

**Fr-4** (6.0 g) was subjected to a Sephadex LH-20 column (bed 5 × 43 cm, in 95% alcohol) and eluted with 95% alcohol to afford five fractions, **Fr-4-1** (0.6 g), **Fr-4-2** (3.0 g), **Fr-4-3** (113.0 mg), **Fr-4-4** (230.0 mg), and **Fr-4-5** (2.0 g). **Fr-4-2** contained the C25 steroids and inhibited the K562 cells with an IR% value of 37.6% at 100 µg*/*mL. **Fr-4-2** (3.0 g) was further separated by Sephadex LH-20 column chromatography (bed 1.8 × 130 cm in DM 1:1, eluting with DM 1:1) to give fractions **Fr-4-2-1** (51.0 mg), **Fr-4-2-2** (0.4 g), and **Fr-4-2-3** (2.0 g). These fractions inhibited the K562 cells with the IR% values of 36.3% (**Fr-4-2-1**), 81.2% (**Fr-4-2-2**), and 23.4% (**Fr-4-2-3**) at 100 µg*/*mL, respectively, and **Fr-4-2-3** contained the targeted C25 steroids. **Fr-4-2-3** (2.0 g) was subjected again to a Sephadex LH-20 column (bed 1.8 × 80 cm, in DM 1:1), and the elution of the column using DM 1:1 afforded fractions **Fr-4-2-3-1** (33.0 mg), **Fr-4-2-3-2** (130.0 mg), and **Fr-4-2-3-3** (1.5 g). The IR% values of the fractions on the K562 cells were 34.3% (**Fr-4-2-3-1**), 65.2% (**Fr-4-2-3-2**), and 23.0% (**Fr-4-2-3-3**) at 100 μg/mL, respectively, and the fraction **Fr-4-2-3-3** contained the C25 steroids. **Fr-4-2-3-3** (1.5 g) was subjected to VLC separation on an ODS column (bed 3 × 15 cm) eluting with MeOH (M)–H_2_O (W) 50:50 → 100:0 to afford two crude C25 steroid fractions, **Fr-4-2-3-3-2** (0.7 g, eluted by MW 82:18) and **Fr-4-2-3-3-3** (0.5 g, eluted by MW 82:18).

**Fr-4-2-3-3-2** (0.7 g) was separated by preparative HPLC (column: Capcell Pak C18, UG120Å, 20 mm × 250 mm; column temperature: room temperature; mobile phase: MeOH–H_2_O in linear gradient 50% MeOH at initial time 0 min → 66% MeOH at 35 min → 72% MeOH at 55 min; flow rate: 5.0 mL/min; detecting wave lengths: 210 and 246 nm) to give **1** (8 mg, *t*_R_ = 55.6 min), **3** (7 mg, *t*_R_ = 66.3 min), **4** (6 mg, *t*_R_ = 46.3 min), **5** (13 mg, *t*_R_ = 48.1 min), **6** (15 mg, *t*_R_ = 45.7 min), **7** (12 mg, *t*_R_ = 47.1 min), **8** (5 mg, *t*_R_ = 60.9 min), **9** (22 mg, *t*_R_ = 62.9 min), **10** (20 mg, *t*_R_ = 42.9 min), **11** (22 mg, *t*_R_ = 43.5 min), **12** (16 mg *t*_R_ = 67.6 min), and **13** (24 mg, *t*_R_ = 65.1 min), together with a mixture of **2** and **14** (18 mg, *t*_R_ = 52.1 min). The mixture (18 mg) of **2** and **14** was then separated by semi-preparative HPLC (column: reversed-phase Click β-CD, 8 mm × 150 mm; column temperature: room temperature; mobile phase: 46% MeOH; flow rate: 2.0 mL/min; detecting wave lengths 210 and 246 nm) to obtain **2** (7 mg, *t*_R_ = 28.9 min) and **14** (7 mg, *t*_R_ = 37.7 min). **Fr-4-2-3-3-3** (0.5 g) was also separated by preparative HPLC at the same conditions as mentioned for **Fr-4-2-3-3-2** except for the different linear gradient (66% MeOH at initial time 0 min → 66% MeOH at 35 min → 70% MeOH at 55 min) to obtain additional amounts of **1** (2 mg, *t*_R_ = 51.4 min), **3** (4 mg, *t*_R_ = 67.3 min), **12** (8 mg, *t*_R_ = 70.4 min), and **13** (5 mg, *t*_R_ = 64.6 min). HPLC profiles for the separation of the fractions **Fr-4-2-3-3-2** and **Fr-4-2-3-3-3**, and the mixture of **2** and **14** were given in Figure S6 in the Supplementary Information.

### 3.5. Physicochemical and Spectroscopic Data for **1**–**14**

Antineocyclocitrinol A (**1**): colorless needles (MeOH), mp 178–179 °C, 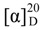 +71.1 (*c* 0.23, MeOH). Positive ESIMS *m*/*z*: 347 [M − 3H_2_O + H]^+^, 365 [M − 2H_2_O + H]^+^, 383 [M − H_2_O + H]^+^, 401 [M + H]^+^, 423 [M + Na]^+^, 439 [M + K]^+^, 823 [2M + Na]+; negative ESI-MS *m*/*z*: 363 [M − 2H_2_O − H]^−^, 381 [M − H_2_O − H]^−^, 399 [M − H]^−^, 435 [M + Cl]^−^, 445 [M + HCOO]^−^, 799 [2M − H]−, 835 [2M + Cl]^−^. Positive HRESIMS *m*/*z*: measured 401.2689 [M + H]^+^, calculated for C_2__5_H_3__7_O_4_ [M + H]^+^ 401.2692; measured 423.2508 [M + Na]^+^, calculated for C_2__5_H_3__6_O_4_Na [M + Na]^+^ 423.2511. UV λ_max_ nm (log ε) in MeOH: 203.7 (3.97), 243.8 (3.95). IR ν_max_ cm^−1^ (Diamond ATR crystal): 3517, 3295, 2966, 2938, 1645, 1460, 1433, 1368, 1294, 1128, 1078, 1012, 915, 899, 869, 827, 764. CD Δε (nm) in MeOH: +4.61 (200.0), 0 (217.0), −0.28 (221), 0 (225.0), +2.57 (247.0), 0 (276.5), −1.38 (316.5), 0 (355.0), +0.16 (372.0), 0 (398.0). ^1^H and ^13^C NMR data: see Table 1 and Table 2, respectively.

Antineocyclocitrinol B (**2**): colorless needles (MeOH), mp 201–202 °C, 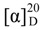 +71.6 (*c* 0.30, MeOH). Positive ESIMS *m*/*z*: 347 [M − 3H_2_O + H]^+^, 365 [M − 2H_2_O + H]^+^, 383 [M − H_2_O + H]^+^, 401 [M + H]^+^, 423 [M + Na]^+^, 439 [M + K]^+^; negative ESI-MS *m*/*z*: 363 [M − 2H_2_O − H]^−^, 381 [M − H_2_O − H]^−^, 399 [M − H]^−^, 435 [M + Cl]^−^. Positive HRESIMS *m*/*z*: measured 401.2684 [M + H]^+^, calculated for C_2__5_H_3__7_O_4_ [M + H]^+^ 401.2692; measured 423.2500 [M + Na]^+^, calculated for C_2__5_H_3__6_O_4_Na [M + Na]^+^ 423.2511. UV λ_max_ nm (log ε) in MeOH: 205.4 (4.11), 243.0 (4.10). IR ν_max_ cm^−1^ (Diamond ATR crystal): 3353, 2956, 2874, 1641, 1460, 1380, 1295, 1176, 1153, 1125, 1078, 1025, 1008, 915, 897, 867, 822, 763. CD Δε (nm) in MeOH: +0.31 (200.0), 0 (216.0), −0.49 (220.0), 0 (224.0), +3.69 (245.0), 0 (275.5), −2.03 (317.0), 0 (374.0). ^1^H and ^13^C NMR data: see Table 1 and Table 2, respectively.

23-*O*-Methylantineocyclocitrinol (**3**): white amorphous powder (MeOH), 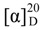 +77.4 (*c* 0.38, MeOH). Positive ESIMS *m*/*z*: 347 [M − CH_3_OH − 2H_2_O + H]^+^, 365 [M − CH_3_OH − H_2_O + H]^+^, 383 [M − CH_3_OH + H]^+^, 415 [M + H]^+^, 437 [M + Na]^+^, 453 [M + K]^+^, 851 [2M + Na]+, 867 [2M + K]+; negative ESI-MS *m*/*z*: 363 [M − CH_3_OH − H_2_O − H]^−^, 381 [M − CH_3_OH − H]^−^, 413 [M − H]^−^, 449 [M + Cl]^−^. Positive HRESIMS *m*/*z*: measured 415.2850 [M + H]^+^, calculated for C_2__6_H_3__9_O_4_ [M + H]^+^ 415.2848; measured 437.2671 [M + Na]^+^, calculated for C_2__6_H_3__8_O_4_Na [M + Na]^+^ 437.2668. UV λ_max_ nm (log ε) in MeOH: 205.4 (4.14), 243.8 (4.10). IR ν_max_ cm^−1^ (Diamond ATR crystal): 3374, 2946, 2910, 2878, 1651, 1587, 1459, 1378, 1297, 1153, 1026, 1007, 916, 869, 820, 761. CD Δε (nm) in MeOH: +1.28 (200.0), +2.69 (205.0), 0 (212.5), −1.13 (217.5), 0 (225.0), +4.10 (246.0), 0 (276.0), −2.15 (318.0), 0 (352.5), +0.45 (371.5), +0.08 (400.0). ^1^H and ^13^C NMR data: see Table 2 and Table 3, respectively.

Neocyclocitrinol B (**4**): colorless needles (MeOH), mp 206–208 °C, 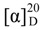 +126.1 (*c* 0.38, MeOH). Positive ESIMS *m*/*z*: 365 [M − 2H_2_O + H]^+^, 401 [M + H]^+^, 423 [M + Na]^+^, 823 [2M + Na]+; negative ESI-MS m/z: 381 [M − H2O − H]−, 399 [M − H]−, 445 [M + HCOO]−, 799 [2M − H]−. CD Δε (nm) in MeOH: +8.45 (200.0), +10.17 (203.5), +2.05 (221.5), +4.40 (245.0), 0 (277), −2.55 (318.0), 0 (364.5), 0.08 (372.0), 0 (390.0), −0.02 (400.0). ^1^H and ^13^C NMR data: see Table 1 and Table 2, respectively.

The data for **5**–**14** except for the ^1^H and ^13^C NMR data (Table 1, Table 2 and Table 3) are in the Supplementary Information.

### 3.6. ICD Measurements for **1**, **2**, **4**–**7** and **14** Using Dimolybdenum Tetracetate

The CD data were acquired at room temperature with a 1-mm cell, and step scans were collected at 0.5 nm per step with an integration time of 0.5 seconds over the range 200–500 nm. The ICD spectra were measured for **1**, **2**, **4**–**7** and **14** (the molecular weight were all 400 daltons) as follows. First, the 175 µg compound was dissolved in 350 µL DMSO (0.5 µg/µL, 0.00125 M/L) and the CD data were recorded immediately. Second, 350 µg dimolybdenum tetracetate (Mo_2_(OAc)_4_, molecular weight 428 daltons) was dissolved in 350 µL DMSO containing 0.00125 M/L compound (the molar concentration of Mo_2_(OAc)_4_ was 0.00234 M/L and the molar ratio of the compound to the Mo_2_(OAc)_4_ was 1:1.87). The first ICD data was recorded immediately after dissolving the Mo_2_(OAc)_4_, its time evolution was monitored with a rate of one spectrum every 3 min, until a stationary ICD was reached about 10 min after dissolving the Mo_2_(OAc)_4_, and then the stationary ICD data were taken. Third, the inherent CD data of the compound were subtracted from the stationary ICD data to obtain the ICD spectrum of Mo-compound complex. The ICD spectrum was normalized to the molar concentration of the compound and is presented as the Δε′ values [22,23]. These Δε′ values are calculated by Δε′ = Δ*A*/*c* × *d*, where *c* is the molar concentration of the compound, assuming 100% complexation (*A* = the difference in absorption of left and right circularly polarized light; *d* = path length of the cell). Δε′ is expressed in [M^−1^ × cm^−^^1^] units.

### 3.7. HPLC-PDAD-UV and HPLC-ESI-MS Analyses

The HPLC-PDAD-UV analysis was carried out on a Venusil MP C18 column (5 μm, 100 Å, 4.6 mm × 250 mm; Agela Technologies, Tianjin, China) using the Waters HPLC equipment mentioned above. Each 5 μL of the sample MeOH solutions at 10 mg/mL was injected into the column and the elution was performed using MeOH–H_2_O in linear gradient (20% MeOH at initial time 0 min → 100% MeOH at 60 min → 100% MeOH at 90 min; flow rate, 1 mL/min). The acquired photodiode array (PDA) data were processed using the Empower PDA software to obtain targeted HPLC-PDAD-UV data. By analyzing both HPLC profiles at different wave lengths and UV absorption curves of related peaks, the peaks of C25 steroids in the range of 40–52 min retention times were authenticated to be recently appeared in the mutant AD-1-2 extract compared to the control G59 extract (Figure S1 in the Supplementary Information).

The HPLC-ESI-MS analysis was performed on an LC-MS equipment equipped with Agilent 1100 HPLC system, AB Sciex API 3000 LC-MS/MS system and AB Sciex Analyst 1.4 software. HPLC was carried out on the Venusil MP C18 column (5 μm, 100 Å, 4.6 mm × 250 mm; Agela Technologies, Tianjin, China) at the same conditions mentioned for the HPLC-PDAD-UV analysis. The mass detector was set to scan a range from *m*/*z* 150–1500 both in the positive and negative modes. The acquired data were processed by Analyst 1.4 software to obtain targeted HPLC-ESI-MS data. The C25 steroids were examined by selective ion monitoring ([M + H]^+^, [M + NH_4_]^+^, [M + Na]^+^, and [M + K]^+^ for the positive MS; [M − H]^−^, [M + Cl]^−^, [M + HCO_2_]^−^, and [M + H_3_C_2_O_2_]^−^ for the negative MS) with both extracted ion chromatograms and related MS spectra. In the HPLC-ESI-MS analysis, both positive and negative ion peaks of the C25 steroids appeared with the retention times shortened approximately 3 min than in HPLC-PDAD-UV analysis because of the shortened flow length from the outlet of the HPLC column to the inlet of MS, and further supported the results of the HPLC-PDAD-UV analysis. Several data from the HPLC-ESI-MS analysis is given in Figure S2 in the Supplementary Information.

## 4. Conclusions

Three new (**1**–**3**) and 11 known (**4**–**14**) unusual C25 steroids with bicyclo[4.4.1]A/B rings were isolated by tracing newly produced metabolites in the EtOAc extract of an antitumor mutant AD-1-2 that was obtained by the DES mutagenesis of a marine-derived fungus *Penicillium purpurogenum* G59. HPLC-PDAD-UV and HPLC-ESI-MS analyses showed that the original G59 strain did not produce these metabolites and demonstrated that the production of **1**–**14** in the mutant AD-1-2 extract was caused by the activation of silent metabolites in original G59 strain by DES mutagenesis. Structures of the new compounds including their absolute configurations were determined by spectroscopic methods, especially the NMR and Mo_2_-induced CD spectra analyses. Compounds **1**–**3** are the first examples of unusual C25 bicyclo[4.4.1]A/B ring steroids with the *Z*-configuration of 20,22-double bond. All **1**–**14** weakly inhibited several human cancer lines to varying extent. The present results provided additional examples for the successful application of the chemical mutagenesis strategy using DES to discover new compounds by activating silent metabolites in fungal isolates [17,19] and supported also the effectiveness and usefulness of this new strategy [17].

## Supplementary Files

Supplementary File 1 Supplementary Information (PDF, 4441 KB)
