# Supplementary material for: Three New and Eleven Known Unusual C25 Steroids: Activated Production of Silent Metabolites in a Marine-Derived Fungus by Chemical Mutagenesis Strategy using Diethyl Sulphate
Source: Mar Drugs. 2014 Mar 13;12(3):1545–68. doi: 10.3390/md12031545 (PMC3967226; doi:10.3390/md12031545)
Supplement: Supplementary File 1 — Supplementary Information (PDF, 4441 KB) [file marinedrugs-12-01545-s001.pdf]

Supplementary Information

Table of Contents

Table S1. 400 MHz ^{1}H and 100 MHz ^{13}C NMR data of **1** in DMSO-d_6 2
Table S2. 400 MHz ^{1}H and 100 MHz ^{13}C NMR data of **2** in DMSO-d_6 3
Table S3. 400 MHz ^{1}H and 100 MHz ^{13}C NMR data of **3** in DMSO-d_6 5
Figure S1. HPLC-PDAD-UV analysis of the EtOAc extracts of the mutant AD-1-2 and the G59 strain 7
Figure S2. HPLC-ESI-MS analysis of the EtOAc extracts of the mutant AD-1-2 and the G59 strain 9
S1. Preparative TLC Separation of the Mutant AD-1-2 Extract for Catching Newly Produced C25 Steroids 13
Figure S3. Preparative TLC chromatograms of the EtOAc extract of the mutant AD-1-2 13
Figure S4. HPLC-PDAD-UV analysis for the TLC fractions (B1–B6) of the mutant EtOAc extract 14
Figure S5. TLC analysis of the 42 eluent portions from silica gel VLC of the mutant EtOAc extract 17
Figure S6. TLC analysis for eluent portions from the separation of **Fr-4** 18
Figure S7. HPLC profiles for the isolation of **1**–**14** by preparative and semi-preparative HPLC 19
S2. Data of Known Steroids. Physicochemical and Spectroscopic Data for **5**–**14** 20
S3. Appendix of Spectra 22
Figure S8. Spectra of **1** 22
Figure S9. Spectra of **2** 34
Figure S10. Spectra of **3** 46

Table S1. 400 MHz ^{1}H and 100 MHz ^{13}C NMR data of **1** in DMSO-d_6 [a].

Position	δ_C [b,c]		δ_H (*J* in Hz) [b]	COSY [d]	NOE [e]	HMBC [f]
1	122.1 d		5.55 dd (8.0, 6.4)	Hα-2, Hβ-2	Hα-2, H-3, H-9	C-2, C-3, C-9, C-18
2	36.0 t	Hα	2.07 m	H-1, Hβ-2	H-1, Hβ-2, H-3, HO-3	C-1, C-3, C-4, C-10
		Hβ	2.34 ddd (13.0, 11.4, 6.4)	H-1, Hα-2, H-3	Hα-2, Hβ-4, Hβ-18, HO-3	C-1, C-3, C-4, C-10
3	63.1 d		3.12 m	Hβ-2, Hβ-4, HO-3	H-1, Hα-2, Hα-4, HO-3	
4	41.4 t	Hα	2.63 br d (13.1)	Hβ-4, H-5	H-3, Hβ-4, H-5, HO-3	
		Hβ	1.53 m	H-3, Hα-4, H-5	Hβ-2, Hα-4, H-5, Hβ-18, HO-3	C-2, C-3, C-6
5	48.1 d		2.69 m	Hα-4, Hβ-4, Hα-18, Hβ-18	Hα-4, Hβ-4, Hα-18, Hβ-18	
6	204.1 s		–	–	–	–
7	124.5 d		5.43 s	H-14	Hβ-15, H_3-19	C-5, C-8, C-9, C-14
8	157.0 s		–	–	–	–
9	53.2 d		2.86 dd (12.0, 5.5)	Hα-11, Hβ-11	H-1, Hα-11, Hα-12, H-14	C-1, C-7, C-8, C-10, C-11, C-18
10	145.5 s		–	–	–	–
11	27.2 t	Hα	1.54 m	H-9, Hβ-11, Hα-12, Hβ-12	H-9, Hβ-11	C-8
		Hβ	1.75 m	H-9, Hα-11, Hα-12, Hβ-12	Hα-11, Hα-18, H_3-19	
12	36.7 t	Hα	1.41 td (12.7, 4.1)	Hα-11, Hβ-11, Hβ-12	H-9, Hβ-12, H-14, H-17	C-13, C-19
		Hβ	1.63 m	Hα-11, Hβ-11, Hα-12	Hα-12, H_3-19	C-14
13	47.8 d		–	–	–	–
14	54.2 d		2.26 br t (8.9)	H-7, Hα-15, Hβ-15	H-9, Hα-12, Hα-15, Hα-16	C-8, C-13, C-15, C-19
15	22.6 t	Hα	1.62 m	H-14, Hα-16, Hβ-16	H-14, Hβ-15, Hα-16	
		Hβ	1.62 m	H-14, Hα-16, Hβ-16	H-7, Hα-15, Hβ-16, H_3-19	
16	24.1 t	Hα	1.66 m	Hβ-16, H-17	Hα-15, Hβ-16, H-17	C-15
		Hβ	1.84 m	Hα-15, Hβ-15, Hα-16, H-17	Hβ-15, Hα-16, H_3-19, H_3-21	C-15
17	50.9 d		2.89 br t (9.8)	Hα-16, Hβ-16	Hα-12, Hα-16, H-23	C-13, C-15, C-16, C-19, C-20, C-21
18	27.2 t	Hα	2.48 br s	H-5	H-5, Hβ-11, H_3-19	C-1, C-4, C-5, C-6, C-9, C-10
		Hβ	2.48 br s	H-5	Hβ-2, Hβ-4, H-5	C-1, C-4, C-5, C-6, C-9, C-10
19	14.8 q		0.64 s	–	H-7, Hβ-11, Hβ-12, Hβ-15, Hβ-16, Hα-18, H_3-21	C-12, C-13, C-14, C-17
20	136.7 s		–	–	–	–
21	22.2 q		1.70 s	H-22	Hβ-16, H_3-19, H-22	C-17, C-20, C-22

Table S1. *Cont.*

22	130.7 d	5.33 d (9.2)	H_3-21, H-23	H_3-21, H-23, H-24, H_3-25, HO-23, HO-24	C-17, C-21, C-23, C-24
23	70.4 d	3.99 ddd (9.2, 6.0, 4.6)	H-22, H-24, HO-23	H-17, H-22, H-24, H_3-25, HO-23, HO-24	C-24
24	70.1 d	3.41 m	H-23, H_3-25, HO-24	H-22, H-23, H_3-25, HO-23, HO-24	C-22
25	19.80 q	1.00 d (6.3)	H-24	H-22, H-23, H-24, HO-23, HO-24	C-23, C-24
3-OH	–	4.62 d (4.3)	H-3	Hα-2, Hβ-2, H-3, Hα-4, Hβ-4	C-2, C-3, C-4
23-OH	–	4.30 d (4.6)	H-23	H-22, H-23, H-24, H_3-25	C-22, C-23, C-24
24-OH	–	4.31 d (4.7)	H-24	H-22, H-23, H-24, H_3-25	C-23, C-24, C-25

[a] Signal assignments were based on the results of DEPT, ^{1}H–^{1}H COSY, HMQC, HMBC, NOESY, and 1D difference NOE experiments. [b] Chemical shifts were recorded in *δ* values using the solvent DMSO-d_6 signals (δ_H 2.50 and δ_C 39.52) as references, respectively. [c] Multiplicities of the carbon signals were determined by DEPT experiments and are shown as s (singlet), d (doublet), t (triplet) and q (quartet), respectively. [d] Numbers in each line of this column indicate the protons that correlated with the proton in the corresponding line in ^{1}H–^{1}H COSY. [e] Numbers in each line of this column indicate the protons that showed NOE correlations with the proton in the corresponding line in 2D NOESY or 1D difference NOE experiments. The NOEs between two protons in a spin coupling relationship were detected by the 1D difference NOE experiments. [f] Numbers in each line of this column indicate the carbons that showed HMBC correlations with the proton in the corresponding line in the HMBC experiments optimized for the 8.3 Hz of long-range J_{CH} value.

Table S2. 400 MHz ^{1}H and 100 MHz ^{13}C NMR data of **2** in DMSO-d_6 [a].

Position	δ_C [b,c]		δ_H (*J* in Hz) [b]	COSY [d]	NOE [e]	HMBC [f]
1	122.1 d		5.55 dd (8.2, 6.4)	Hα-2, Hβ-2	Hα-2, H-3, H-9	C-2, C-3, C-9, C-18
2	36.0 t	Hα	2.07 m	H-1, Hβ-2	H-1, Hβ-2, H-3, HO-3	C-1, C-3, C-4, C-10
		Hβ	2.34 ddd (13.2, 11.2, 6.4)	H-1, Hα-2, H-3	Hα-2, Hβ-4, Hβ-18, HO-3	C-1, C-3, C-4, C-10
3	63.1 d		3.12 m	Hβ-2, Hβ-4, HO-3	H-1, Hα-2, Hα-4, HO-3	
4	41.4 t	Hα	2.63 br d (12.8)	Hβ-4, H-5	H-3, Hβ-4, H-5, HO-3	
		Hβ	1.52 m	H-3, Hα-4, H-5	Hβ-2, Hα-4, H-5, Hβ-18, HO-3	C-3, C-6
5	48.1 d		2.69 m	Hα-4, Hβ-4, Hα-18, Hβ-18	Hα-4, Hβ-4, Hα-18, Hβ-18	
6	204.1 s		–	–	–	–
7	124.5 d		5.43 s	H-14	Hβ-15, H_3-19	C-5, C-8, C-9, C-14
8	157.1 s		–	–	–	–
9	53.3 d		2.86 dd (11.3, 5.6)	Hα-11, Hβ-11	H-1, Hα-11, Hα-12, H-14	C-1, C-7, C-8, C-10, C-11, C-18
10	145.5 s		–	–	–	–

Table S2. *Cont.*

11	27.3 t	Hα	1.52 m	H-9, Hβ-11, Hα-12, Hβ-12	H-9, Hβ-11	C-8
		Hβ	1.74 m	H-9, Hα-11,Hα-12, Hβ-12	Hα-11, Hα-18, H_3-19	C-10
12	36.6 t	Hα	1.40 td (12.8, 4.3)	Hα-11, Hβ-11, Hβ-12	H-9, Hβ-12, H-14, H-17	C-19
		Hβ	1.69 m	Hα-11, Hβ-11, Hα-12	Hα-12, H_3-19	C-7, C-13, C-14, C-17
13	47.8 s		–	–	–	–
14	54.3 d		2.25 br t (8.6)	H-7, Hα-15, Hβ-15	H-9, Hα-12, Hα-15, Hα-16	C-7, C-8, C-13, C-16, C-19
15	22.6 t	Hα	1.61 m	H-14, Hα-16, Hβ-16	H-14, Hβ-15, Hα-16	C-16
		Hβ	1.61 m	H-14, Hα-16, Hβ-16	H-7, Hα-15, Hβ-16, H_3-19	C-16
16	24.0 t	Hα	1.69 m	Hβ-16, H-17	Hα-15, Hβ-16, H-17	C-15
		Hβ	1.85 m	Hα-15, Hβ-15, Hα-16, H-17	Hβ-15, Hα-16, H_3-19, H_3-21	C-15
17	51.0 d		2.86 t (9.5)	Hα-16, Hβ-16	Hα-12, Hα-16, H-23	C-13, C-15, C-16, C-19, C-20, C-21
18	27.2 t	Hα	2.48 br s	H-5	H-5, Hβ-11, H_3-19	C-1, C-4, C-5, C-6, C-9, C-10
		Hβ	2.48 br s	H-5	Hβ-2, Hβ-4, H-5	C-1, C-4, C-5, C-6, C-9, C-10
19	14.7 q		0.63 s	–	H-7, Hβ-11, Hβ-12, Hβ-15, Hβ-16, Hα-18, H_3-21	C-12, C-13, C-14, C-17
20	136.6 s		–	–	–	–
21	22.3 q		1.70 d (1.1)	H-22	Hβ-16, H_3-19, H-22	C-17, C-20, C-22
22	130.8 d		5.33 dd (9.3, 1.1)	H_3-21, H-23	H_3-21, H-23, H-24, H_3-25, <u>H</u>O-23, <u>H</u>O-24	C-17, C-21
23	70.3 d		4.11 ddd (9.3, 4.9, 4.1)	H-22, H-24, <u>H</u>O-23	H-17, H-22, H-24, H_3-25, <u>H</u>O-23, <u>H</u>O-24	C-20, C-24
24	69.8 d		3.47 m	H-23, H_3-25, <u>H</u>O-24	H-22, H-23, H_3-25, <u>H</u>O-23, <u>H</u>O-24	C-22
25	18.5 q		1.01 d (6.3)	H-24	H-22, H-23, H-24, <u>H</u>O-23, <u>H</u>O-24	C-23, C-24
3-OH	–		4.62 d (4.3)	H-3	Hα-2, Hβ-2, H-3, Hα-4, Hβ-4	C-2, C-3, C-4
23-OH	–		4.28 d (4.9)	H-23	H-22, H-23, H-24, H_3-25	C-22, C-23, C-24
24-OH	–		4.31 d (4.9)	H-24	H-22, H-23, H-24, H_3-25	C-23, C-24, C-25

[a] Signal assignments were based on the results of DEPT, ^1H–^1H COSY, HMQC, HMBC, NOESY, and 1D difference NOE experiments. [b] Chemical shifts were recorded in δ values using the solvent DMSO-d_6 signals (δ_H 2.50 and δ_C 39.52) as references, respectively. [c] Multiplicities of the carbon signals were determined by DEPT experiments and are shown as s (singlet), d (doublet), t (triplet) and q (quartet), respectively. [d] Numbers in each line of this column indicate the protons that correlated with the proton in the corresponding line in ^1H–^1H COSY. [e] Numbers in each line of this column indicate the protons that showed NOE correlations with the proton in the corresponding line in 2D NOESY or 1D difference NOE experiments. The NOEs between two protons in a spin coupling relationship were detected by the 1D difference NOE experiments. [f] Numbers in each line of this column indicate the carbons that showed HMBC correlations with the proton in the corresponding line in the HMBC experiments optimized for the 8.3 Hz of long-range J_{CH} value.

Table S3. 400 MHz ^{1}H and 100 MHz ^{13}C NMR data of **3** in DMSO-d_6 [a].

Position	δ_C [b,c]		δ_H (J in Hz) [b]	COSY [d]	NOE [e]	HMBC [f]
1	122.1 d		5.55 dd (8.1, 6.4)	Hα-2, Hβ-2	Hα-2, H-3, H-9	C-2, 3, 9, 18
2	36.0 t	Hα	2.07 m	H-1, Hβ-2	H-1, Hβ-2, H-3, <u>H</u>O-3	C-1, 3, 4, 10
		Hβ	2.34 ddd (13.3, 11.4, 6.4)	H-1, Hα-2, H-3	Hα-2, Hβ-4, Hβ-18, <u>H</u>O-3	C-1, 3, 4, 10
3	63.1 d		3.11 m	Hβ-2, Hβ-4, <u>H</u>O-3	H-1, Hα-2, Hα-4, <u>H</u>O-3	
4	41.4 t	Hα	2.63 br d (13.0)	Hβ-4, H-5	H-3, Hβ-4, H-5, <u>H</u>O-3	
		Hβ	1.52 m	H-3, Hα-4, H-5	Hβ-2, Hα-4, H-5, Hβ-18, <u>H</u>O-3	C-3, 6
5	48.1 d		2.69 m	Hα-4, Hβ-4, Hα-18, Hβ-18	Hα-4, Hβ-4, Hα-18, Hβ-18	
6	204.1 s		–	–	–	–
7	124.5 d		5.43 s	H-14	Hβ-15, H_3-19	C-8, 9
8	157.0 s		–	–	–	–
9	53.3 d		2.84 dd (11.2, 6.0)	Hα-11, Hβ-11	H-1, Hα-11, Hα-12, H-14	C-1, 7, 8, 10, 11, 18
10	145.5 s		–	–	–	–
11	27.3 t	Hα	1.50 m	H-9, Hβ-11, Hα-12, Hβ-12	H-9, Hβ-11	C-8, 13
		Hβ	1.77 m	H-9, Hα-11, Hα-12, Hβ-12	Hα-11, Hα-18, H_3-19	C-10
12	36.4 t	Hα	1.41 td (12.4, 4.0)	Hα-11, Hβ-11, Hβ-12	H-9, Hβ-12, H-14, H-17	C-19
		Hβ	1.68 m	Hα-11, Hβ-11, Hα-12	Hα-12, H_3-19	C-13
13	47.73/47.66 s		–	–	–	–
14	54.2 d		2.29 br t (7.9)	H-7, Hα-15, Hβ-15	H-9, Hα-12, Hα-15, Hα-16	C-13
15	22.7 t	Hα	1.62 m	H-14, Hα-16, Hβ-16	H-14, Hβ-15, Hα-16	
		Hβ	1.62 m	H-14, Hα-16, Hβ-16	H-7, Hα-15, Hβ-16, H_3-19	
16	24.1/24.0 t	Hα	1.71 m	Hβ-16, H-17	Hα-15, Hβ-16, H-17	C-15
		Hβ	1.90 m	Hα-15, Hβ-15, Hα-16, H-17	Hβ-15, Hα-16, H_3-19, H_3-21	C-15
17	51.2 d		2.84 m (covered by H-9 signals)	Hα-16, Hβ-16	Hα-12, Hα-16, H-23	C-13, 15, 16, 19, 20, 21
18	27.3 t	Hα	2.48 br s	H-5	H-5, Hβ-11, H_3-19	C-1, 4, 5, 6, 9, 10
		Hβ	2.48 br s	H-5	Hβ-2, Hβ-4, H-5	C-1, 4, 5, 6, 9, 10
19	14.7 q		0.65 s/0.67 s		H-7, Hβ-11, Hβ-12, Hβ-15, Hβ-16, Hα-18, H_3-21	C-12, 13, 14, 17
20	140.1/140.0 s		–	–	–	–
21	22.2/22.0 q		1.77 s	H-22	Hβ-16, H_3-19, H-22	C-17, 20, 22

Table S3. *Cont.*

22	126.9 d	5.17 dd (10.2, 1.0)/5.14 dd (10.2, 1.0)	H_3-21, H-23	H_3-21, H-23, H-24, H_3-25, C$\underline{H}_3$O-23, $\underline{H}$O-24	C-17, 21
23	79.9/79.7 d	3.84 dd (10.0, 4.0)/3.74 dd (10.0, 5.9)	H-22, H-24	H-17, H-22, H-24, H_3-25, C$\underline{H}_3$O-23, $\underline{H}$O-24	$\underline{C}H_3$O-23
24	68.6 d	3.56 m/3.50 m	H-23, H_3-25, $\underline{H}$O-24	H-22, H-23, H_3-25, C$\underline{H}_3$O-23, $\underline{H}$O-24	
25	18.8/19.9 q	1.03 d (6.3)/1.01 d (6.3)	H-24	H-22, H-23, H-24, C$\underline{H}_3$O-23, $\underline{H}$O-24	C-23, 24
3-OH	–	4.63 d (4.3)	H-3	Hα-2, Hβ-2, H-3, Hα-4, Hβ-4	C-2, 4
24-OH	–	4.47 d (4.9) /4.44 d (4.7)	H-24	H-22, H-23, H-24, H_3-25, C$\underline{H}_3$O-23	C-23, 24, 25
23-OCH_3	55.0/54.9 q	3.11 s		Hα-16, H_3-21, H-22, H-23, H-24, H_3-25, $\underline{H}$O-24	C-23

[a] Signal assignments were based on the results of DEPT, ^{1}H–^{1}H COSY, HMQC, HMBC, NOESY, and 1D difference NOE experiments. [b] Chemical shifts were recorded in δ values using the solvent DMSO-d_6 signals (δ_H 2.50 and δ_C 39.52) as references, respectively. [c] Multiplicities of the carbon signals were determined by DEPT experiments and are shown as s (singlet), d (doublet), t (triplet) and q (quartet), respectively. [d] Numbers in each line of this column indicate the protons that correlated with the proton in the corresponding line in ^{1}H–^{1}H COSY. [e] Numbers in each line of this column indicate the protons that showed NOE correlations with the proton in the corresponding line in 2D NOESY or 1D difference NOE experiments. The NOEs between two protons in a spin coupling relationship were detected by the 1D difference NOE experiments. [f] Numbers in each line of this column indicate the carbons that showed HMBC correlations with the proton in the corresponding line in the HMBC experiments optimized for the 8.3 Hz of long-range J_{CH} value.

Figure S1. HPLC-PDAD-UV analysis of the EtOAc extracts of the mutant AD-1-2 and the G59 strain. (**A**) HPLC profiles of the mutant AD-1-2 and the control G59 Extracts; (**B**) UV curves of the mutant AD-1-2 and the control G59 Extracts at the same retention times (t_R).

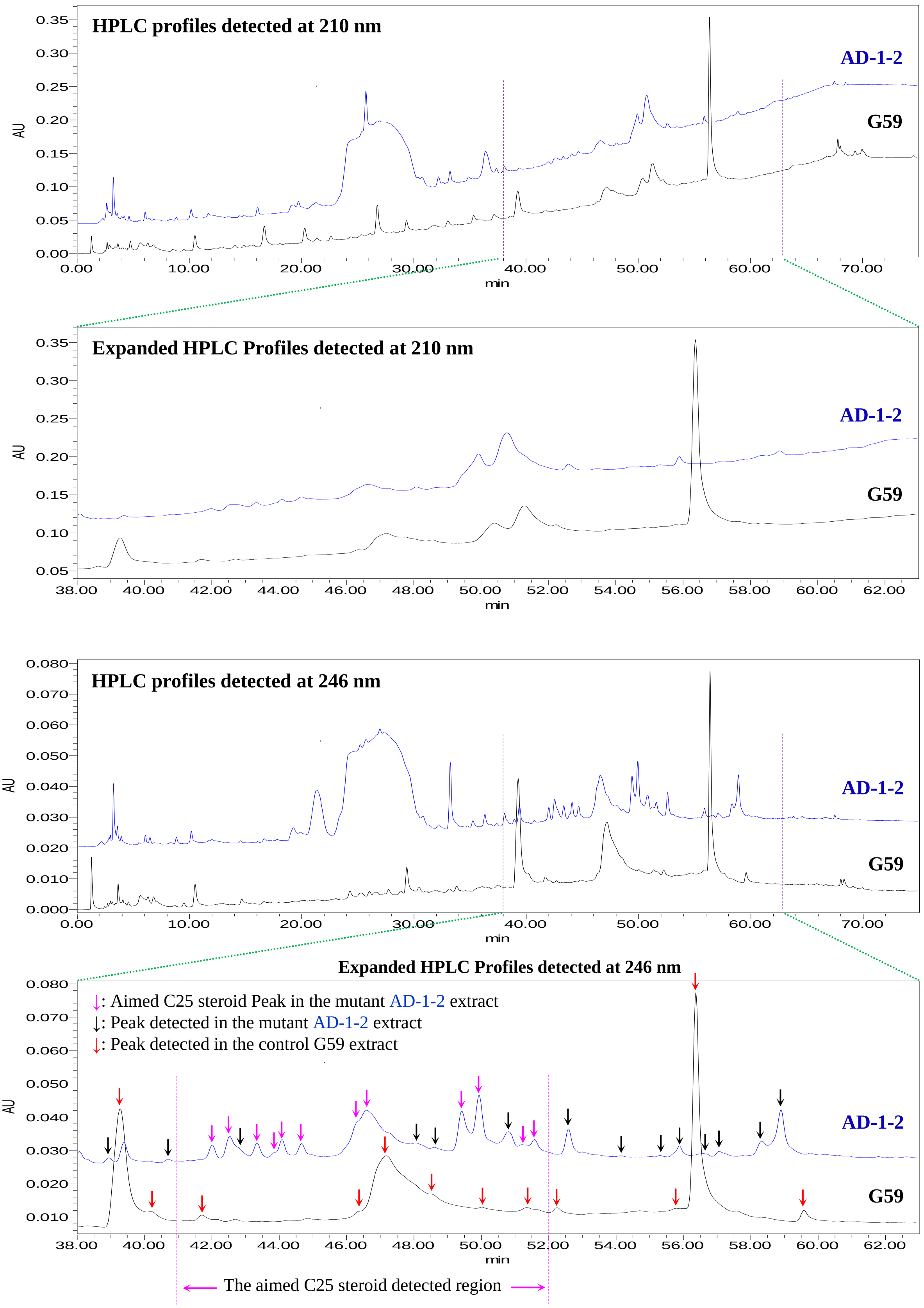

A

Figure S1. *Cont.*

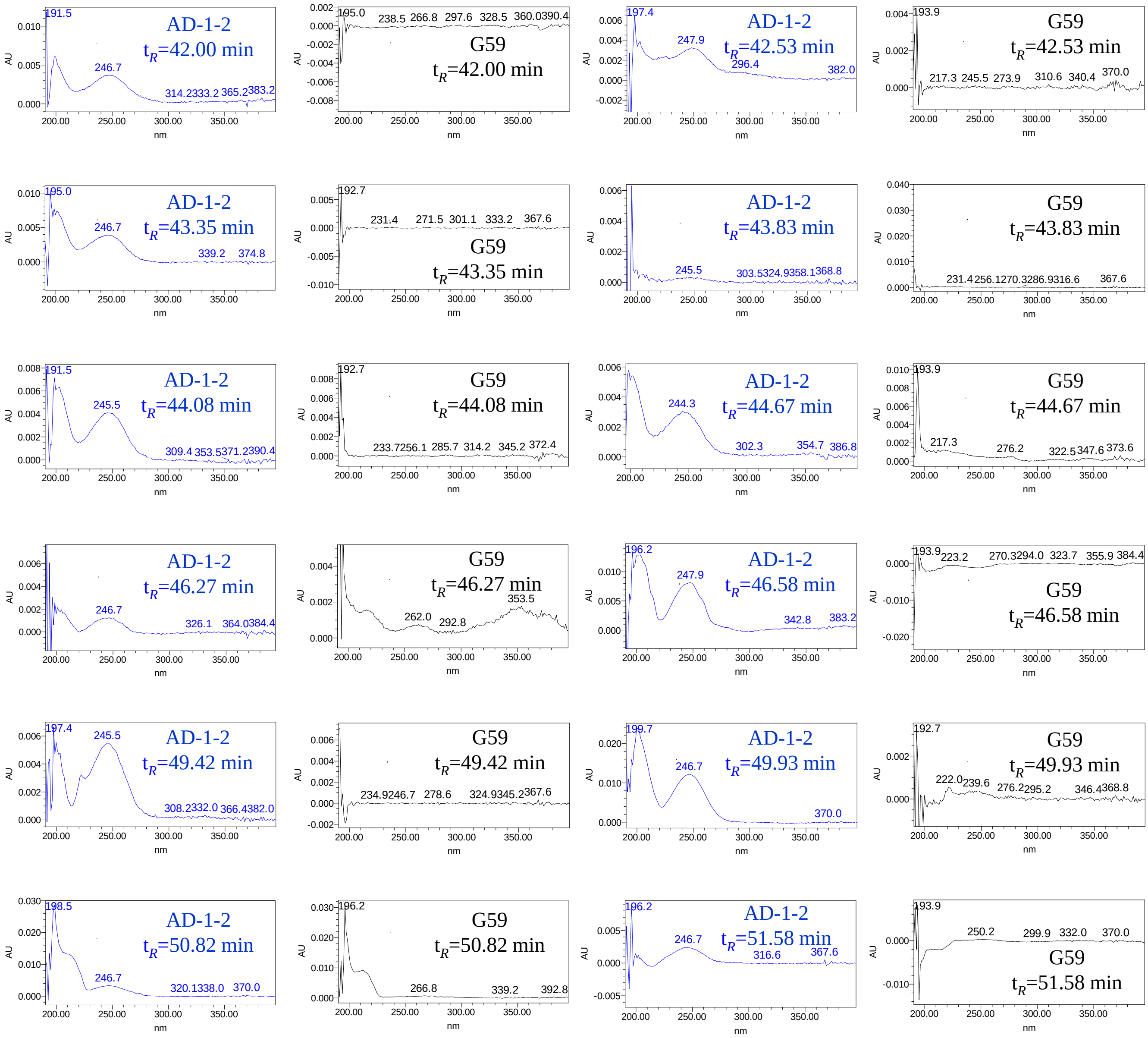

B

Figure S2. HPLC-ESI-MS analysis of the EtOAc extracts of the mutant AD-1-2 and the G59 strain. (**A**) 1. HPLC-Positive ion ESI-MS analysis (ESIMS *m/z*: 423 $[M + Na]^+$ for **4–7**, 2. HPLC-Positive ion ESI-MS analysis (ESIMS *m/z*: 423 $[M + Na]^+$ for **1**, **2** and **14**); (**B**) HPLC-Negative ion ESI-MS analysis (ESIMS *m/z*: 413 $[M - H]^-$ for **9**); (**C**) HPLC-Positive ion ESI-MS analysis (ESIMS *m/z*: 437 $[M + Na]^+$ for **3**, **8** and **12**); (**D**) HPLC-Negative ion ESI-MS analysis (ESIMS *m/z*: 399 $[M - H]^-$ for **10** and **11**); (**E**) HPLC-Positive ion ESI-MS analysis (ESIMS *m/z*: 415 $[M + H]^+$ for **13**).

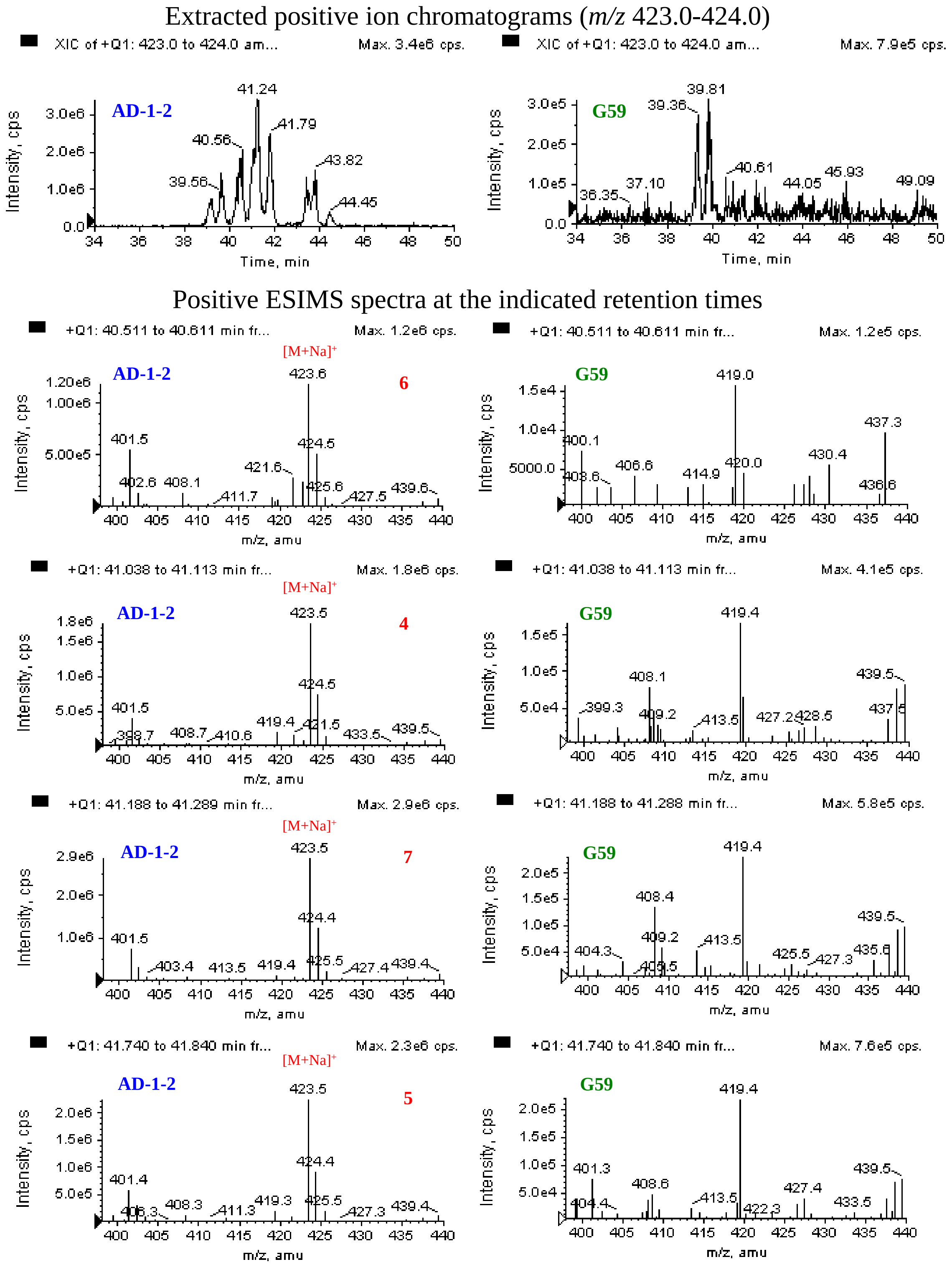

A1

Figure S2. *Cont.*

Positive ESIMS spectra at the indicated retention times

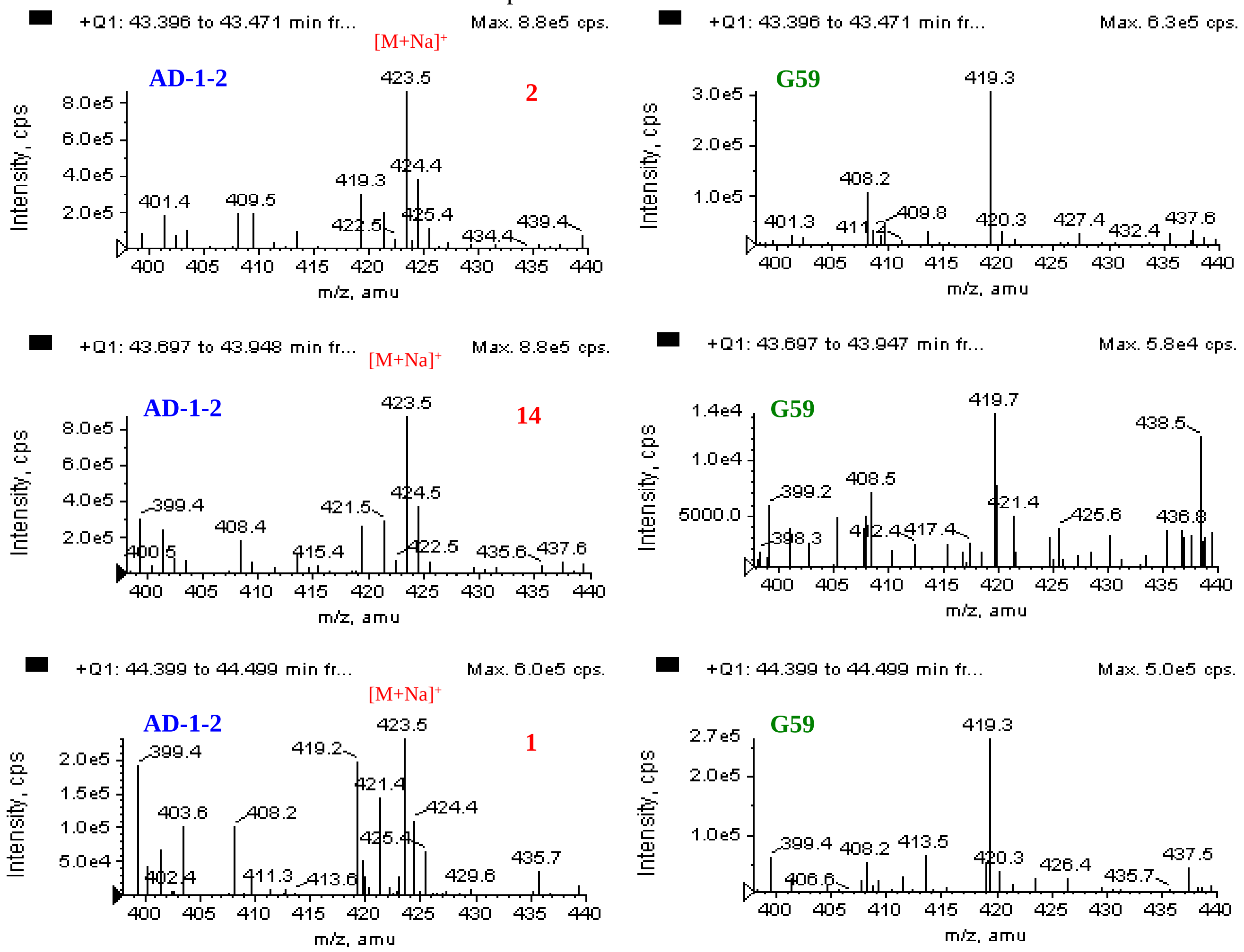

A2

Extracted Negative Ion Chromatograms (*m/z* 413.0–414.0)

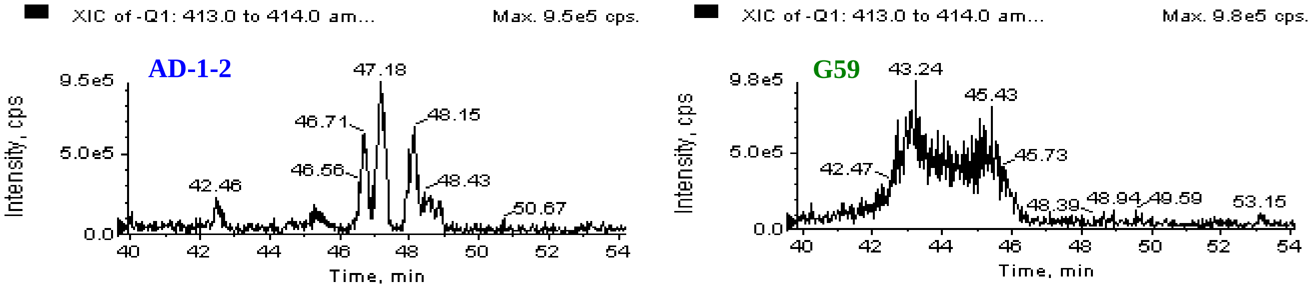

Negative ESIMS spectra at the indicated retention times

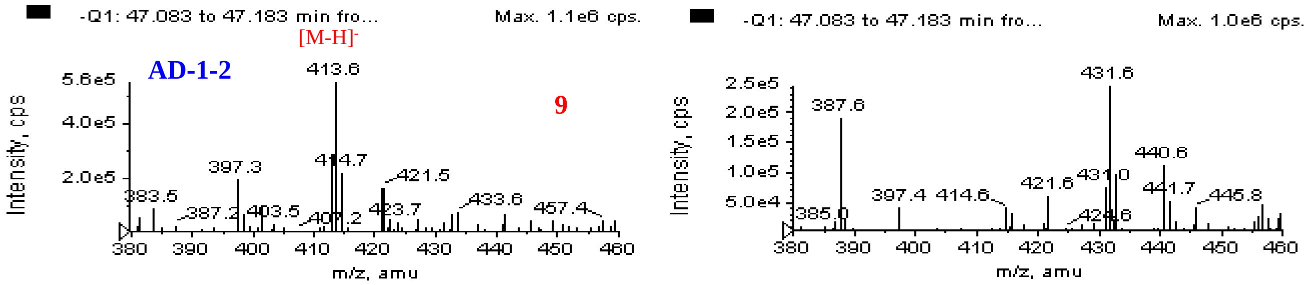

B

Figure S2. *Cont.*

Extracted Positive Ion Chromatograms (*m/z* 437.0–438.0)

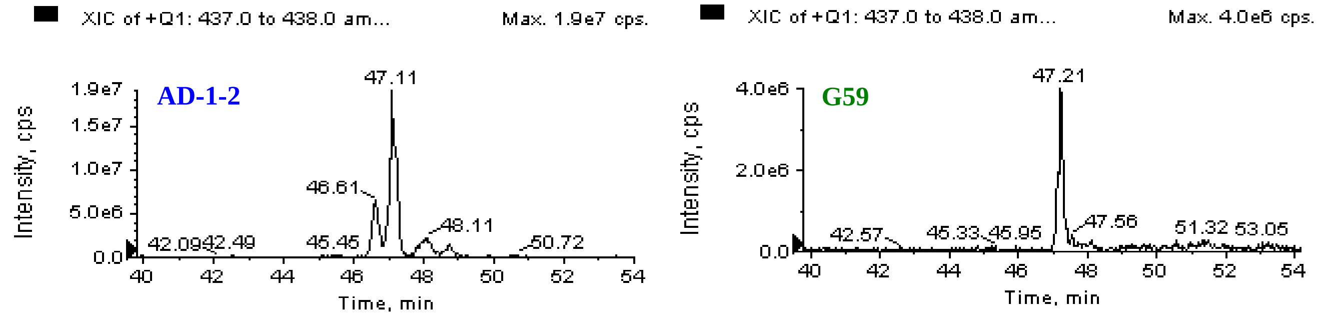

Positive ESIMS spectra at the indicated retention times

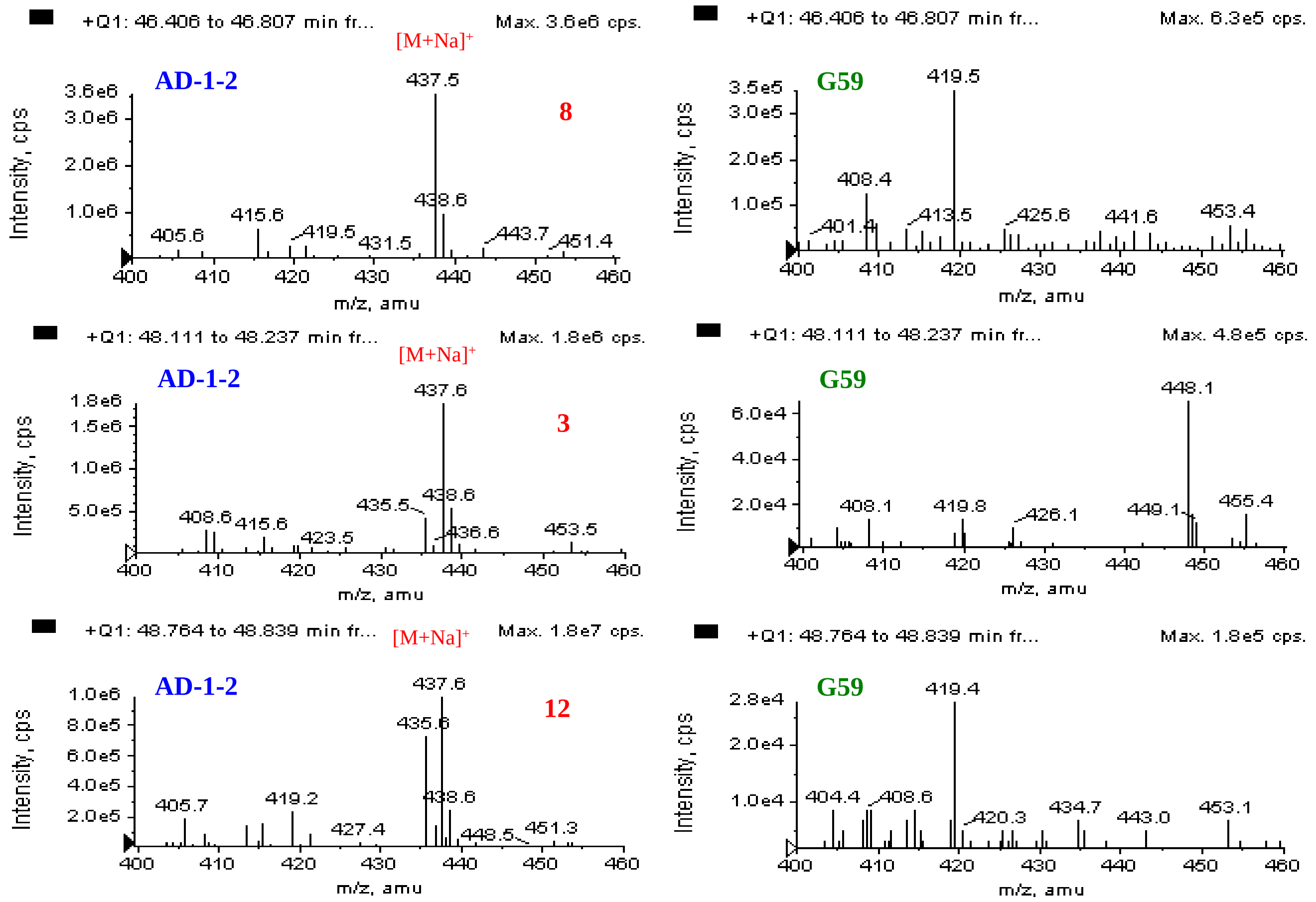

C

Figure S2. *Cont.*

Extracted Negative Ion Chromatograms (*m/z*399.0-400.0)

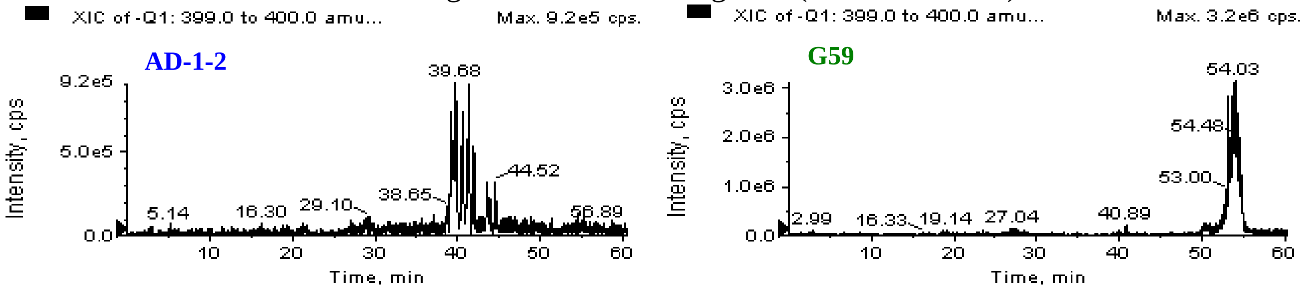

Negative ESIMS spectra at the indicated retention times

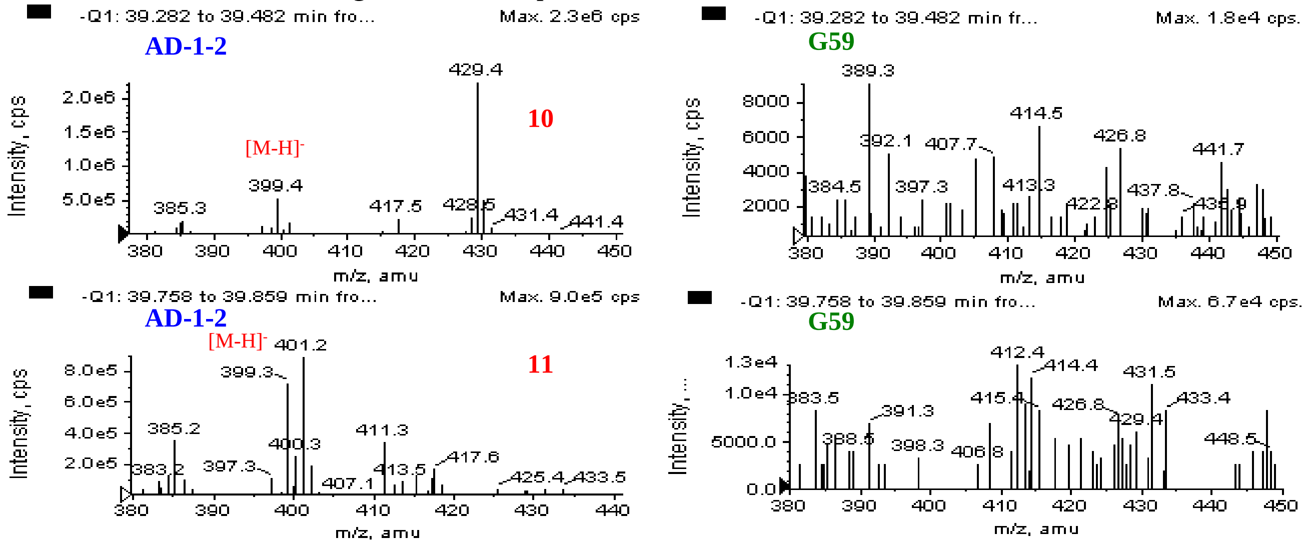

D

Extracted Positive Ion Chromatograms (*m/z* 415.0-416.0)

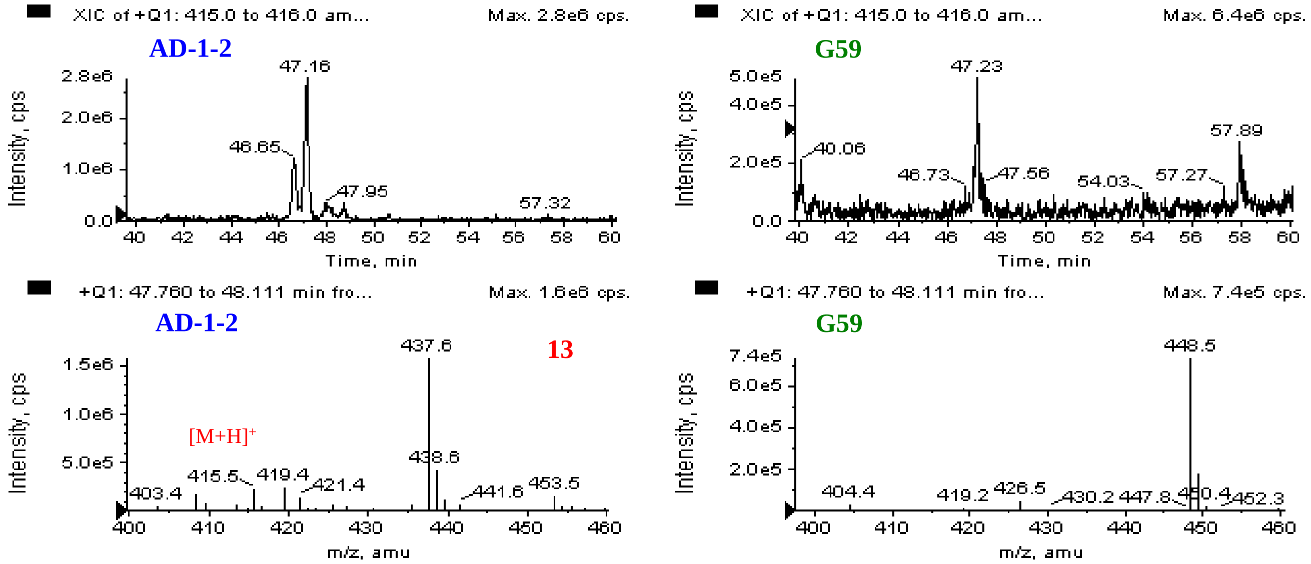

E

S1. Preparative TLC Separation of the Mutant AD-1-2 Extract for Catching Newly Produced C25 Steroids

A preparative TLC separation of the EtAOc extract from the mutant AD-1-2 cultures was performed for catching the newly produced C25 steroid components in the mutant extract. The 10 mg of the mutant EtOAc extract (200 μL MeOH solution at 50 mg/mL) was loaded onto the starting zone of a preparative TLC silica gel plate (9 cm × 10 cm, 0.5 mm thickness), and the plate was developed by CH_2Cl_2–MeOH (9:1) to separate the extract into several bands (Figure S3). Silica gels on the TLC plate were harvested into six portions according to the bands on the plate, shown in Figure S3, and the adsorbed substances were extracted by eluting with each 10 mL of CH_2Cl_2–MeOH 1:1 solution to obtain six TLC fractions B1–B6, respectively.

The TLC fractions B1–B6 were dissolved in each 1.0 mL MeOH to prepare their MeOH solutions at the concentration that was equivalent to the EtOAc extract of the mutant AD-1-2 at 10 mg/mL in the solutions, respectively. The MeOH solutions were then subjected to HPLC-PDAD-UV analysis and MTT assay.

The HPLC-PDAD-UV analysis of the fractions B1–B6 was performed together with the EtOAc extracts of the mutant AD-1-2 and the control G59 strain by comparison of both retention times and UV absorption curves of related peaks. The HPLC-PDAD-UV analysis indicated that the C25 steroids were enriched and existed only in two fractions B3 and B4 (see Figure S4 in the Supplementary Information). In the MTT assay, the fractions B3 and B4 weakly inhibited K562 cells with the IR% values of 18.9% and 23.5% at the 100 μg/mL, respectively. These two fractions were used as reference standards for tracing the aimed C25 steroids in the followed large-scale separation of the mutant EtOAc extract.

Figure S3. Preparative TLC chromatograms of the EtOAc extract of the mutant AD-1-2. Developing solvent: CH_2Cl_2-MeOH 9:1. (**A**) under 254 nm UV light; (**B**) under sunlight; (**C**) by Vaughan's reagent.

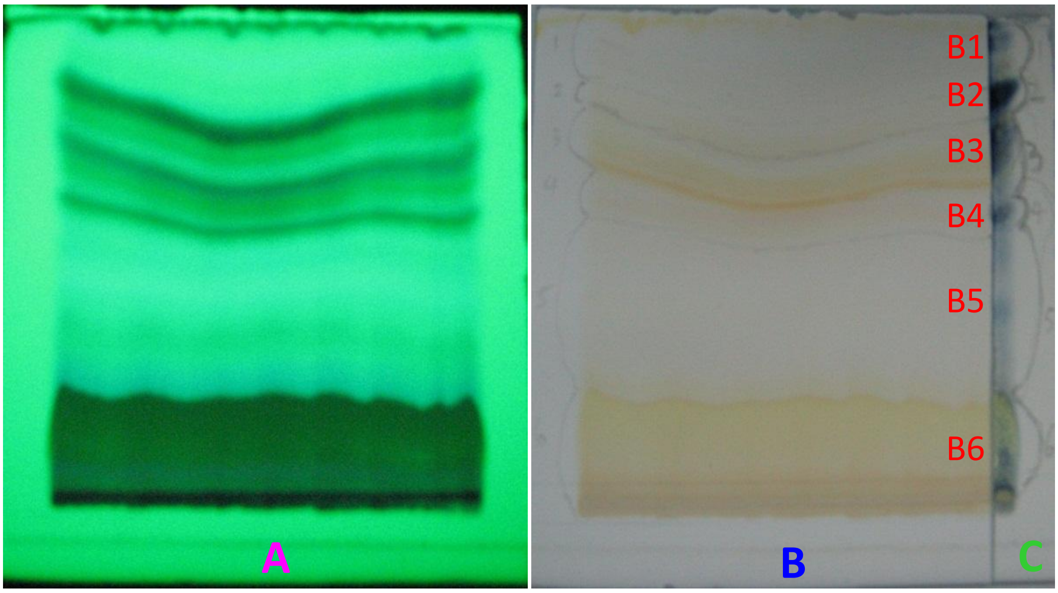

Figure S4. HPLC-PDAD-UV analysis for the TLC fractions B1–B6 of the mutant EtOAc extract. (**A**) HPLC profiles of the TLC fractions B1–B6; (**B**) UV spectra of C25 steroid peaks in B4 and B3 and of G59 extract at same retention times (t_R).

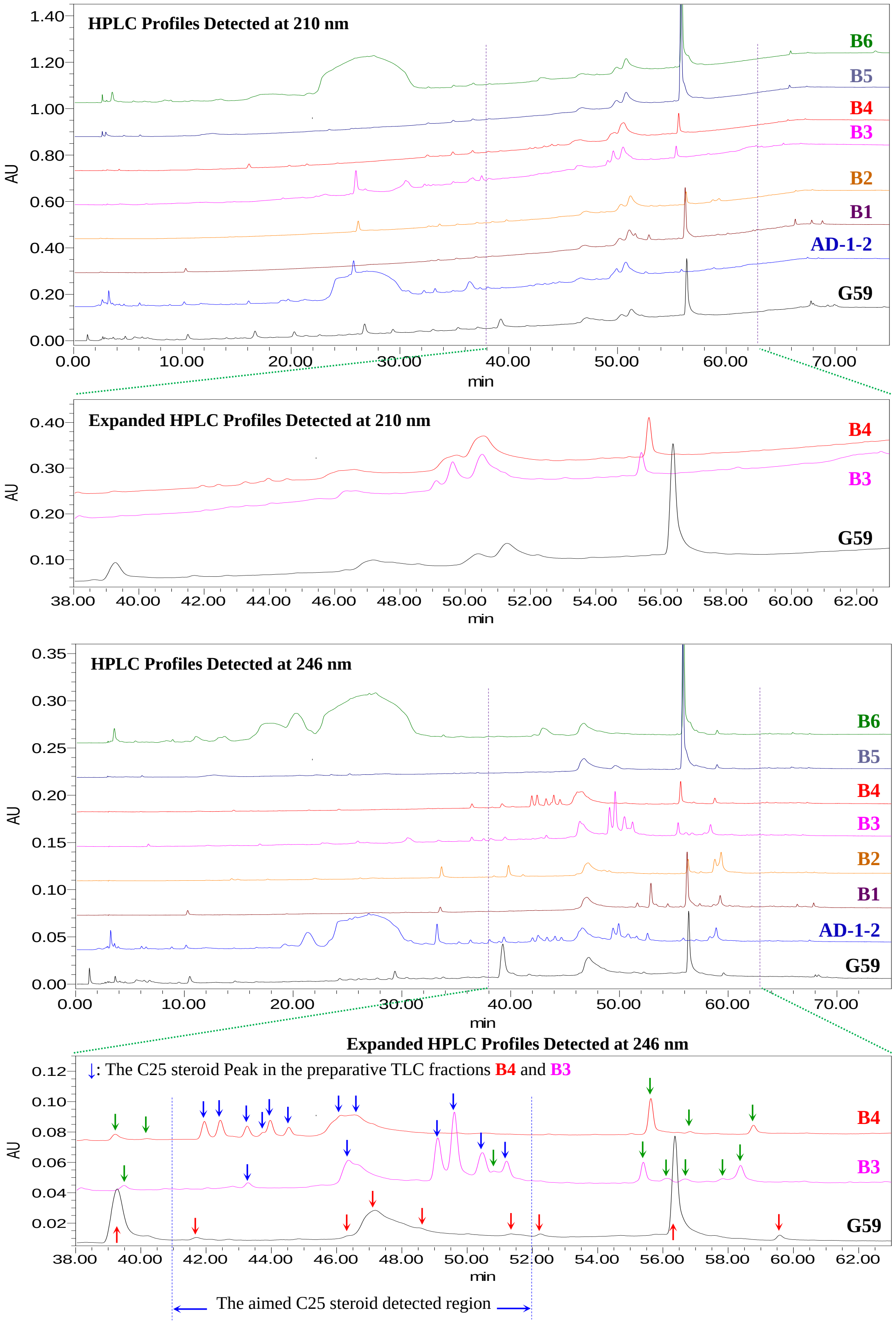

A

Figure S4. *Cont.*

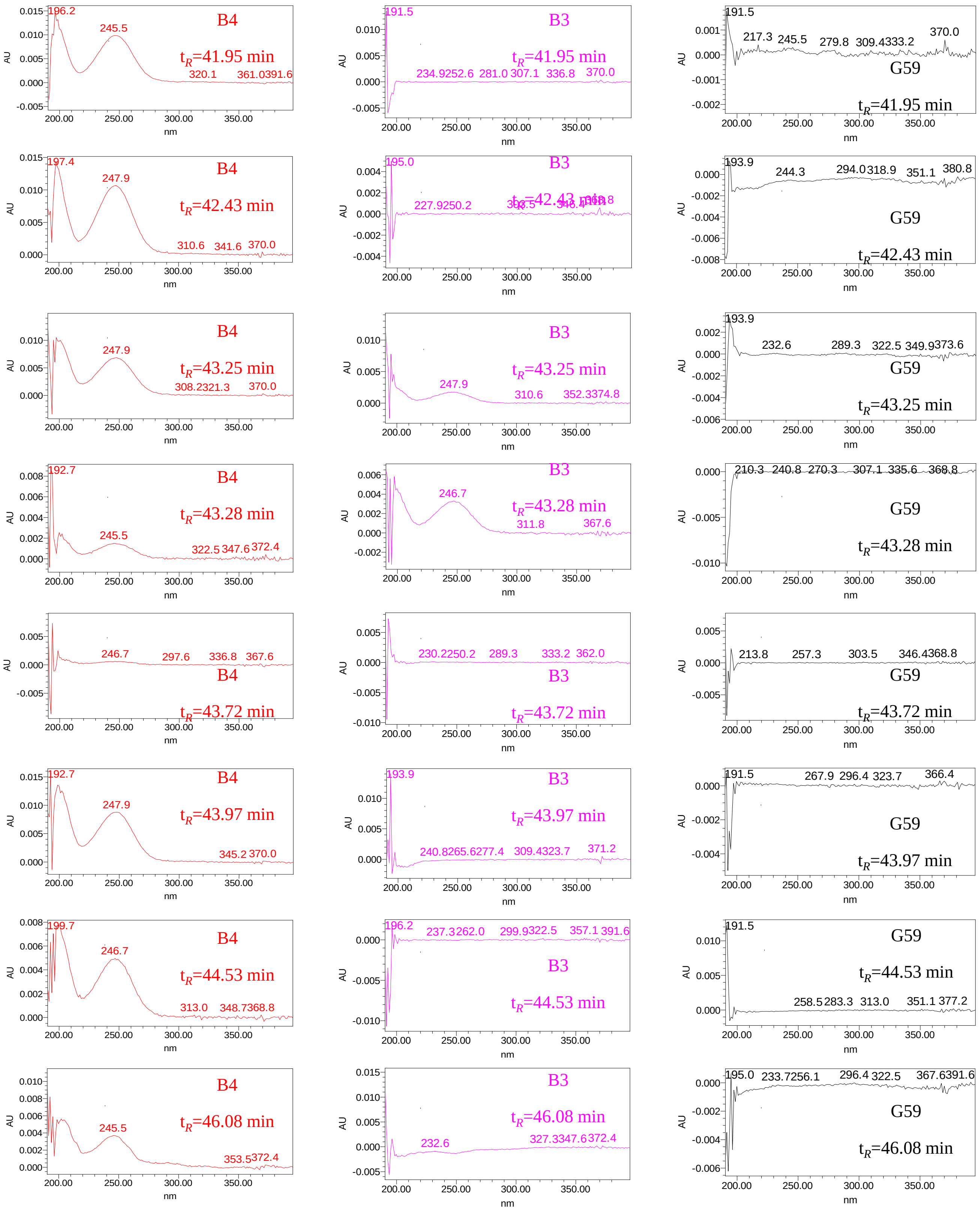

B1

Figure S4. *Cont.*

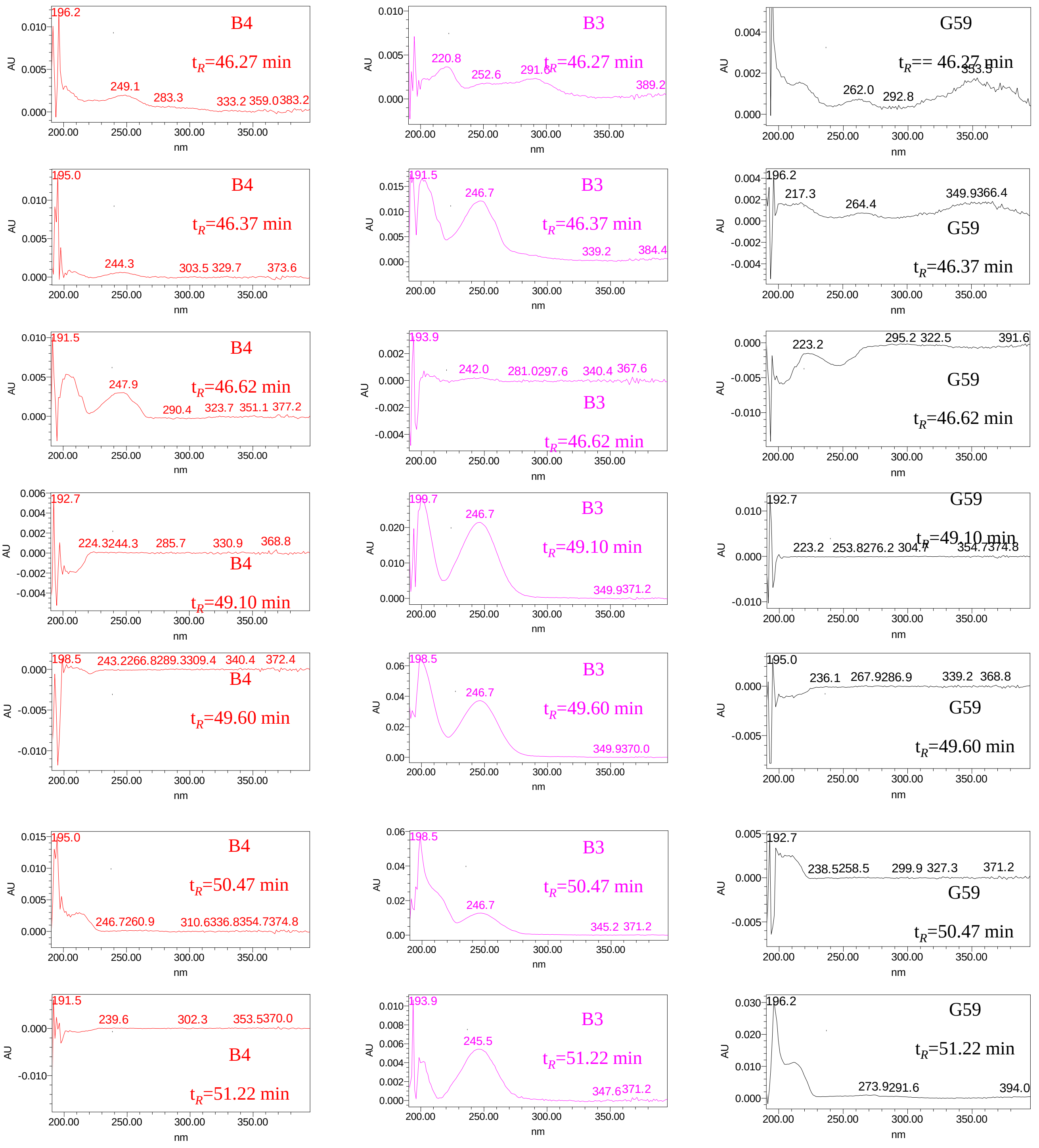

B2

Figure S5. TLC analysis of the 42 eluent portions from silica gel VLC of the mutant EtOAc extract. Developing Solvents: (**A**) CH_2Cl_2–MeOH 20:1; (**B**) CH_2Cl_2–MeOH 10:1; (**C**) CH_2Cl_2–MeOH 3:1. Detection: Plates on up line, UV 254 nm; Plates on down line, Vaughan's reagent.

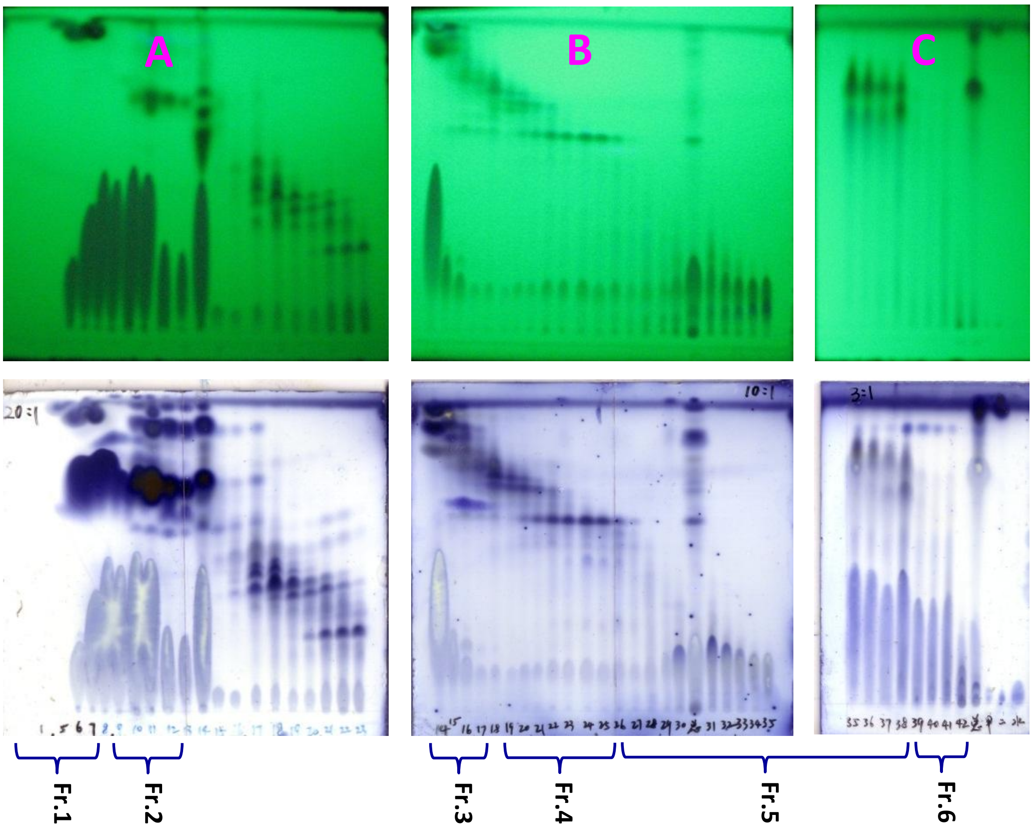

Figure S6. TLC analysis for eluent portions from the separation of **Fr-4**. Developing Solvents: CH_2Cl_2–MeOH 15:1 for **A**–**D**. Detection: Plates on up line, UV 254 nm; Plates on down line, Vaughan's reagent. (**A**) eluent portions from fraction **Fr-4**. (**B**) eluent portions from fraction **Fr-4-2**. (**C**) eluent portions from fraction **Fr-4-2-3**. (**D**) eluent portions from fraction **Fr-4-2-3-3**.

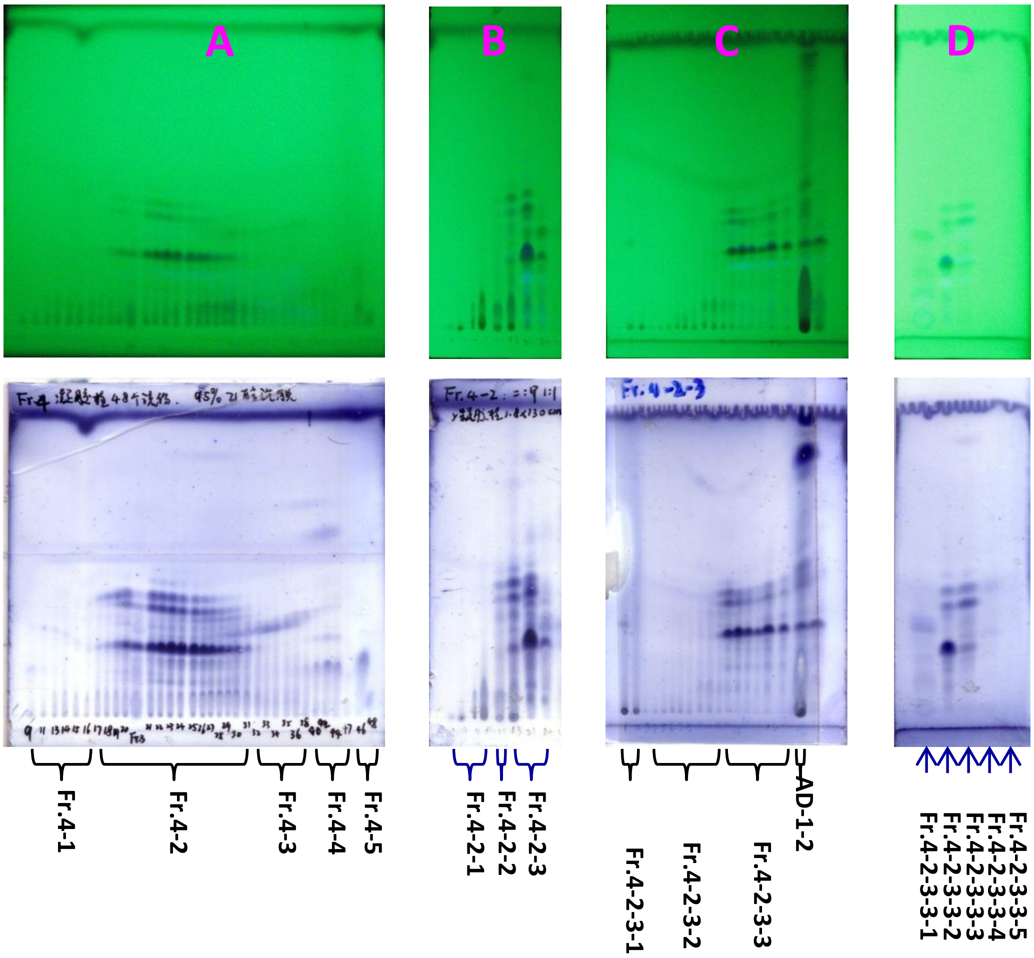

Figure S7. HPLC profiles for the isolation of **1**–**14** by preparative and semi-preparative HPLC. (**A**) Preparative HPLC profiles of **Fr-4-2-3-3-2** and **Fr-4-2-3-3-3**. (**B**) Semi-preparative HPLC profiles for the isolation of the **2** and **14** mixture.

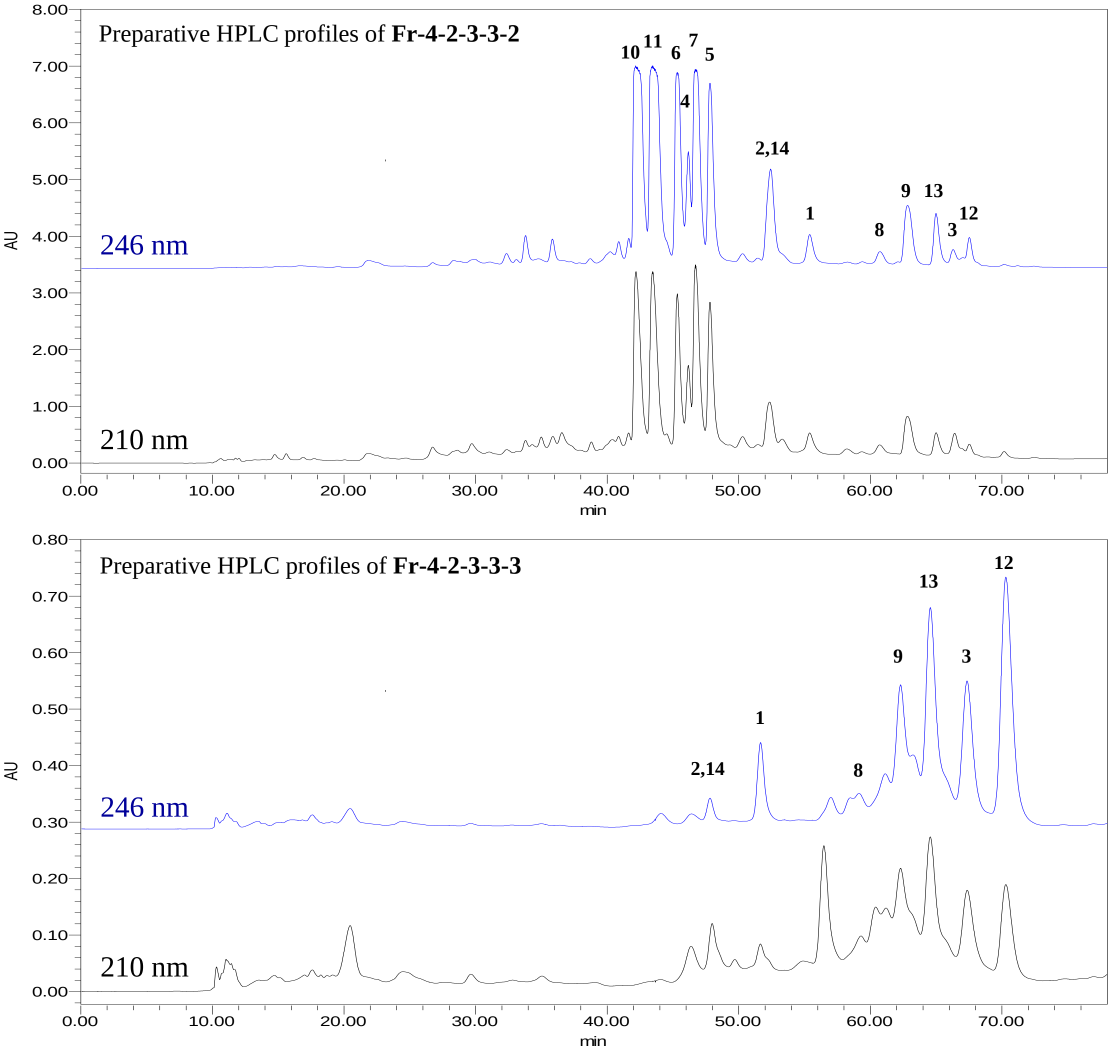

A

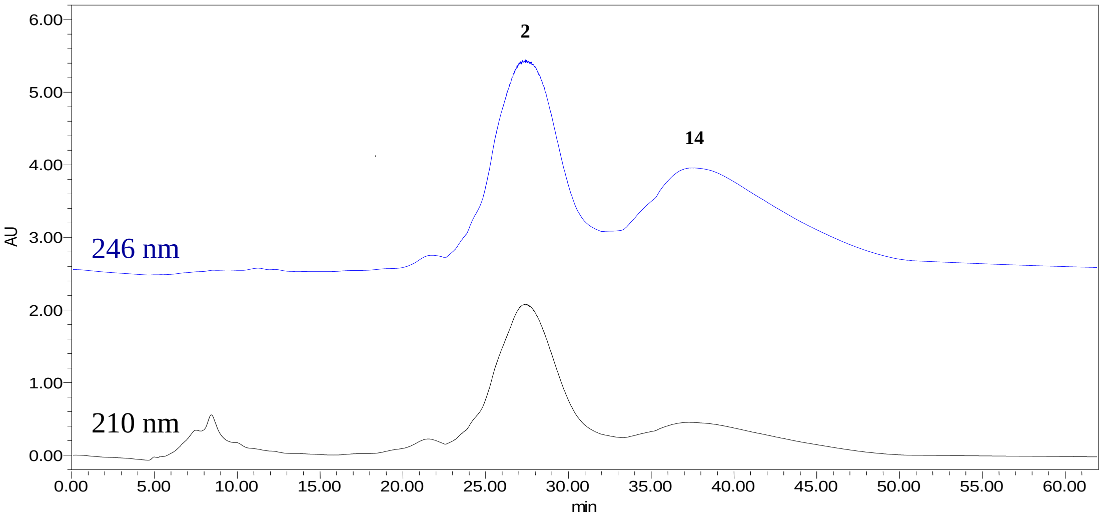

Physicochemical and Spectroscopic Data for **5**–**14**

B

S2. Data of Known Steroids. Physicochemical and Spectroscopic Data for 5–14

Neocyclocitrinol A (**5**): colorless needles (MeOH), mp 198–200 ℃, $[\alpha]_D^{20}$ +137.7 (*c* 0.90, MeOH). Positive ESIMS *m/z*: 365 [M − $2H_2O$ + H]$^+$, 383 [M − H_2O + H]$^+$, 401 [M + H]$^+$, 423 [M + Na]$^+$, 439 [M + K]$^+$, 823 [2M + Na]$^+$; negative ESI-MS *m/z*: 363 [M − $2H_2O$ − H]$^-$, 381 [M − H_2O − H]$^-$, 399 [M − H]$^-$, 445 [M + HCOO]$^-$, 799 [2M − H]$^-$. ^{1}H and ^{13}C NMR data: see Tables 1 and 2 in the main text, respectively.

Neocyclocitrinol D (**6**): colorless needles (MeOH), mp 118–120 ℃, $[\alpha]_D^{20}$ +112.4 (*c* 0.82, MeOH). Positive ESIMS *m/z*: 365 [M − $2H_2O$ + H]$^+$, 401 [M + H]$^+$, 423 [M + Na]$^+$, 439 [M + K]$^+$, 823 [2M + Na]$^+$, 840 [2M + K]$^+$; negative ESI-MS *m/z*: 363 [M − $2H_2O$ − H]$^-$, 381 [M − H_2O − H]$^-$, 399 [M − H]$^-$, 435 [M + Cl]$^-$, 445 [M + HCOO]$^-$, 799 [2M − H]$^-$. ^{1}H and ^{13}C NMR data: see Tables 1 and 2 in the main text, respectively.

Neocyclocitrinol C (**7**): colorless needles (MeOH), mp 200–202 ℃, $[\alpha]_D^{20}$ +113.1 (*c* 0.91, MeOH). Positive ESIMS *m/z*: 365 [M − $2H_2O$ + H]$^+$, 383 [M − H_2O + H]$^+$, 401 [M + H]$^+$, 423 [M + Na]$^+$, 823 [2M + Na]$^+$; negative ESI-MS *m/z*: 381 [M − H_2O − H]$^-$, 399 [M − H]$^-$, 445 [M + HCOO]$^-$, 799 [2M − H]$^-$. ^{1}H and ^{13}C NMR data: see Tables 1 and 2 in the main text, respectively.

***Threo*-23-*O*-methylneocyclocitrinol (8):** white powder (MeOH), $[\alpha]_D^{20}$ +87.2 (*c* 0.40, MeOH). Positive ESIMS *m/z*: 415 [M + H]$^+$, 437 [M + Na]$^+$, 851 [2M + Na]$^+$; negative ESI-MS *m/z*: 413 [M − H]$^-$, 459 [M + HCOO]$^-$. ^{1}H and ^{13}C NMR data: see Tables 2 and 3 in the main text, respectively.

***Erythro*-23-*O*-methylneocyclocitrinol (9):** colorless needles (MeOH), mp 116–118 ℃, $[\alpha]_D^{20}$ +100.2 (*c* 1.00, MeOH). Positive ESIMS *m/z*: 415 [M + H]$^+$, 437 [M + Na]$^+$, 851 [2M + Na]$^+$; negative ESI-MS *m/z*: 413 [M − H]$^-$, 459 [M + HCOO]$^-$. ^{1}H and ^{13}C NMR data: see Tables 2 and 3 in the main text, respectively.

24-*Epi*-cyclocitrinol (10): colorless needles (MeOH), mp 146–148 ℃, $[\alpha]_D^{20}$ +169.0 (*c* 1.00, MeOH). Positive ESIMS *m/z*: 365 [M − $2H_2O$ + H]$^+$, 401 [M + H]$^+$, 423 [M + Na]$^+$, 439 [M + K]$^+$, 823 [2M + Na]$^+$, 839 [2M + K]$^+$; negative ESI-MS *m/z*: 381 [M − H_2O − H]$^-$, 399 [M − H]$^-$, 445 [M + HCOO]$^-$, 799 [2M − H]$^-$. ^{1}H and ^{13}C NMR data: see Tables 1 and 2 in the main text, respectively.

Cyclocitrinol (11): colorless needles (MeOH), mp 118–120 ℃, $[\alpha]_D^{20}$ +178.6 (*c* 1.00, MeOH). Positive ESIMS *m/z*: 365 [M − $2H_2O$ + H]$^+$, 383 [M − H_2O + H]$^+$, 401 [M + H]$^+$, 423 [M + Na]$^+$, 439 [M + K]$^+$, 823 [2M + Na]$^+$, 839 [2M + K]$^+$; negative ESI-MS *m/z*: 381 [M − H_2O − H]$^-$, 399 [M − H]$^-$, 445 [M + HCOO]$^-$, 799 [2M − H]$^-$. ^{1}H and ^{13}C NMR data in DMSO-d_6: see Tables 1 and 2 in the main text, respectively.

20-*O*-Methyl-24-*epi*-cyclocitrinol (12): colorless needles (MeOH), mp 112–114 ℃, $[\alpha]_D^{20}$ +171.7 (*c* 0.92, MeOH). Positive ESIMS *m/z*: 415 [M + H]$^+$, 437 [M + Na]$^+$, 453 [M + K]$^+$, 851 [2M + Na]$^+$; negative ESI-MS *m/z*: 413 [M − H]$^-$. ^{1}H and ^{13}C NMR data: see Tables 2 and 3 in the main text, respectively.

20-*O*-Methylcyclocitrinol (13): colorless needles (MeOH), mp 160–162 ℃, $[\alpha]_D^{20}$ +184.9 (*c* 1.00, MeOH). Positive ESIMS *m/z*: 415 $[M + H]^+$, 437 $[M + Na]^+$, 453 $[M + K]^+$, 852 $[2M + Na]^+$; negative ESI-MS *m/z*: 413 $[M - H]^-$, 827 $[2M - H]^-$. ^1H and ^{13}C NMR data: see Tables 2 and 3 in the main text, respectively.

Isocyclocitrinol B (14): colorless needles (MeOH), mp 199–201 ℃, $[\alpha]_D^{20}$ +189.0 (*c* 0.47, MeOH). Positive ESIMS *m/z*: 401 $[M + H]^+$, 423 $[M + Na]^+$, 801 $[2M + H]^+$, 823 $[2M + Na]^+$; negative ESI-MS *m/z*: 399 $[M - H]^-$, 799 $[2M - H]^-$. ^1H and ^{13}C NMR data: see Tables 2 and 3 in the main text, respectively.

S3. Appendix of Spectra

Figure S8. (**A**) Positive and negative ESIMS spectra of **1**. (**B**) Positive HRESIMS spectrum of **1**. (**C**) UV spectrum of **1** in MeOH. (**D**) IR spectrum of **1**. (**E**) 400 MHz ^{1}H NMR Spectrum of **1** in DMSO-d_6. (**F**) 100 MHz ^{13}C NMR Spectrum of **1** in DMSO-d_6. (**G**) DEPT Spectra of **1** in DMSO-d_6. (**H**) ^{1}H-^{1}H COSY Spectrum of **1** in DMSO-d_6. (**I**) HMQC Spectrum of **1** in DMSO-d_6. (**J**) HMBC Spectrum of **1** in DMSO-d_6. (**K**) NOESY Spectrum of **1** in DMSO-d_6. (**L**) 1D Difference NOE Spectrum of **1** in DMSO-d_6.

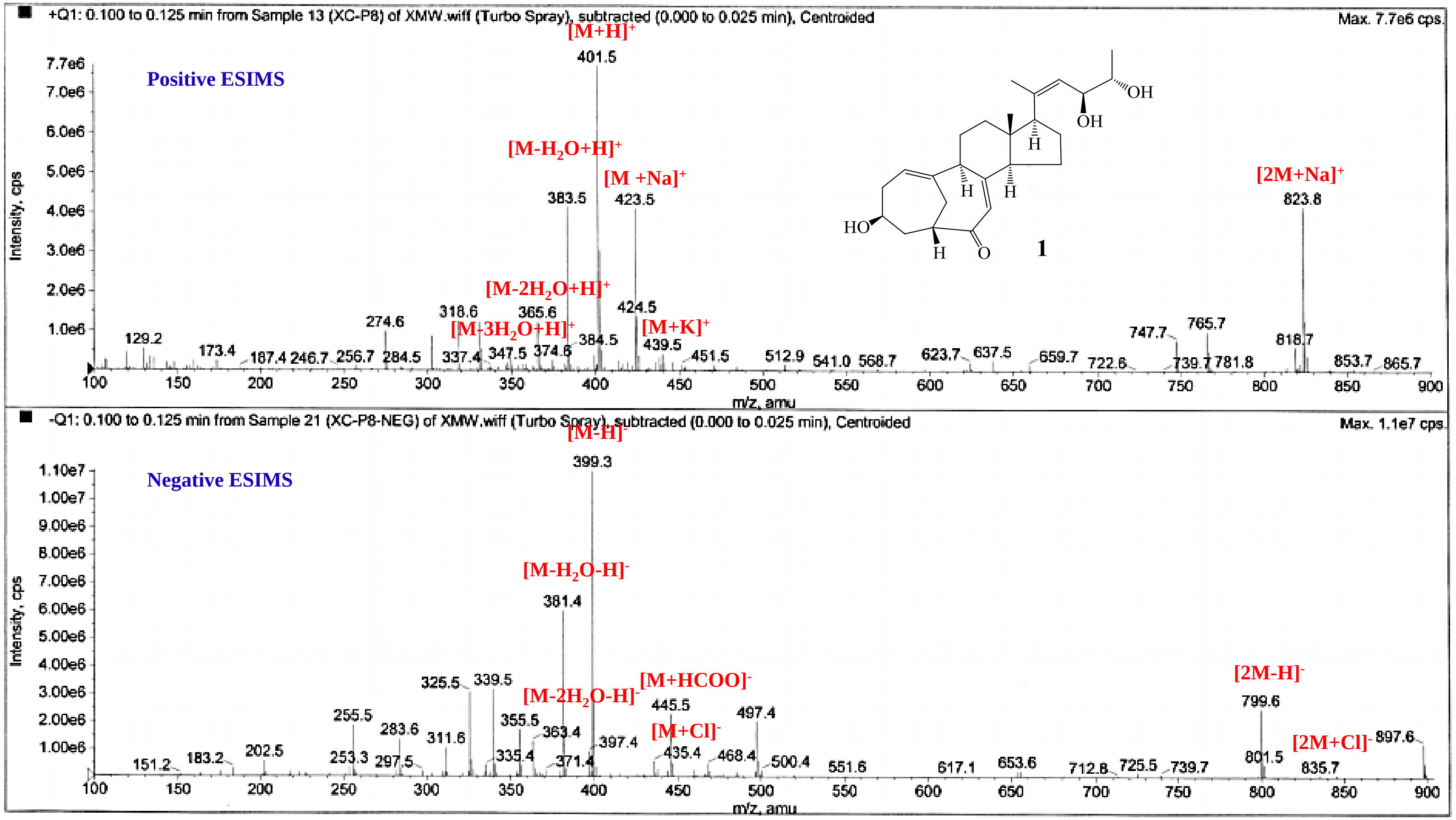

A

Figure S8. *Cont.*

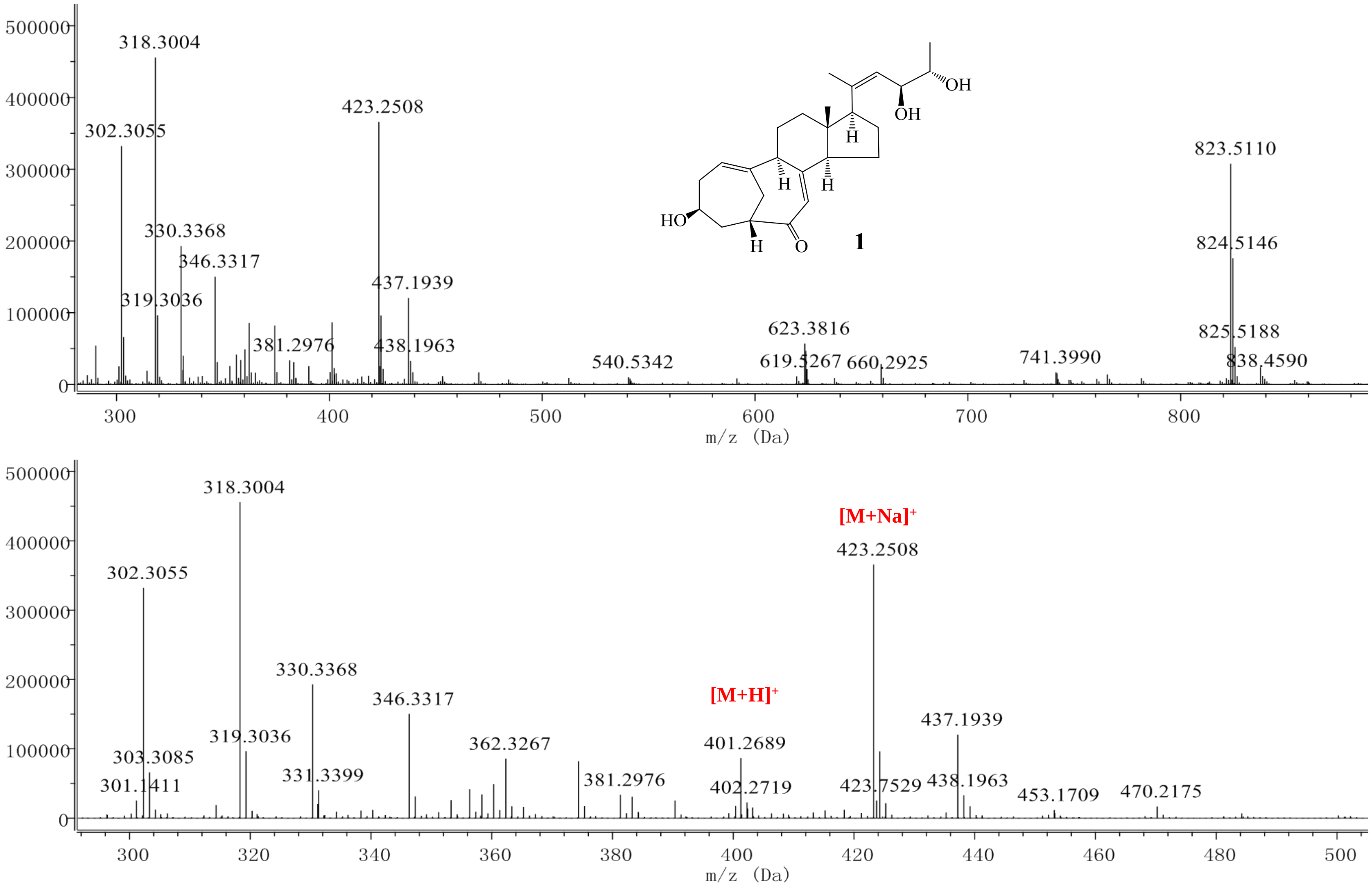

B

Figure S8. *Cont.*

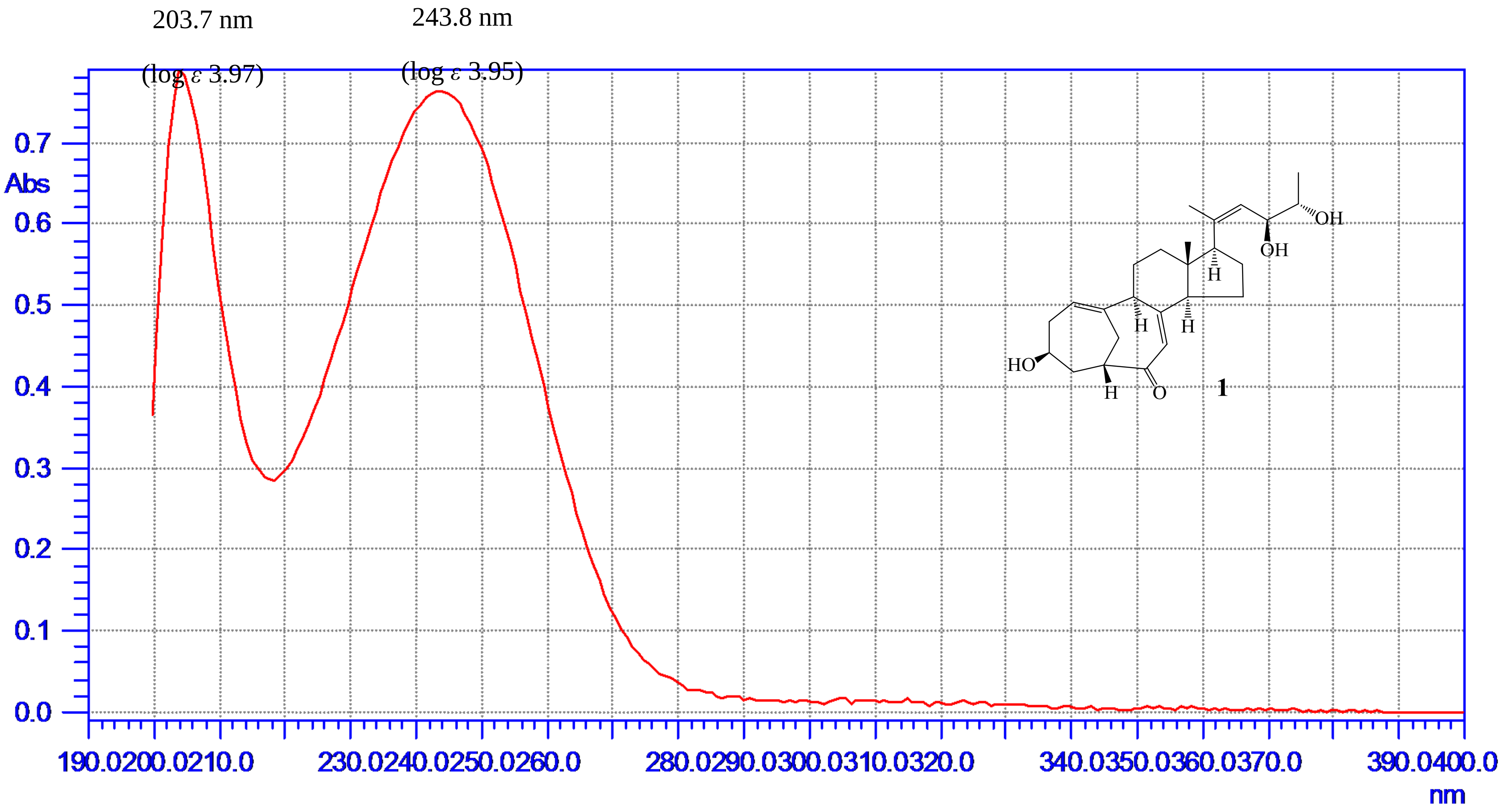

C

Figure S8. *Cont.*

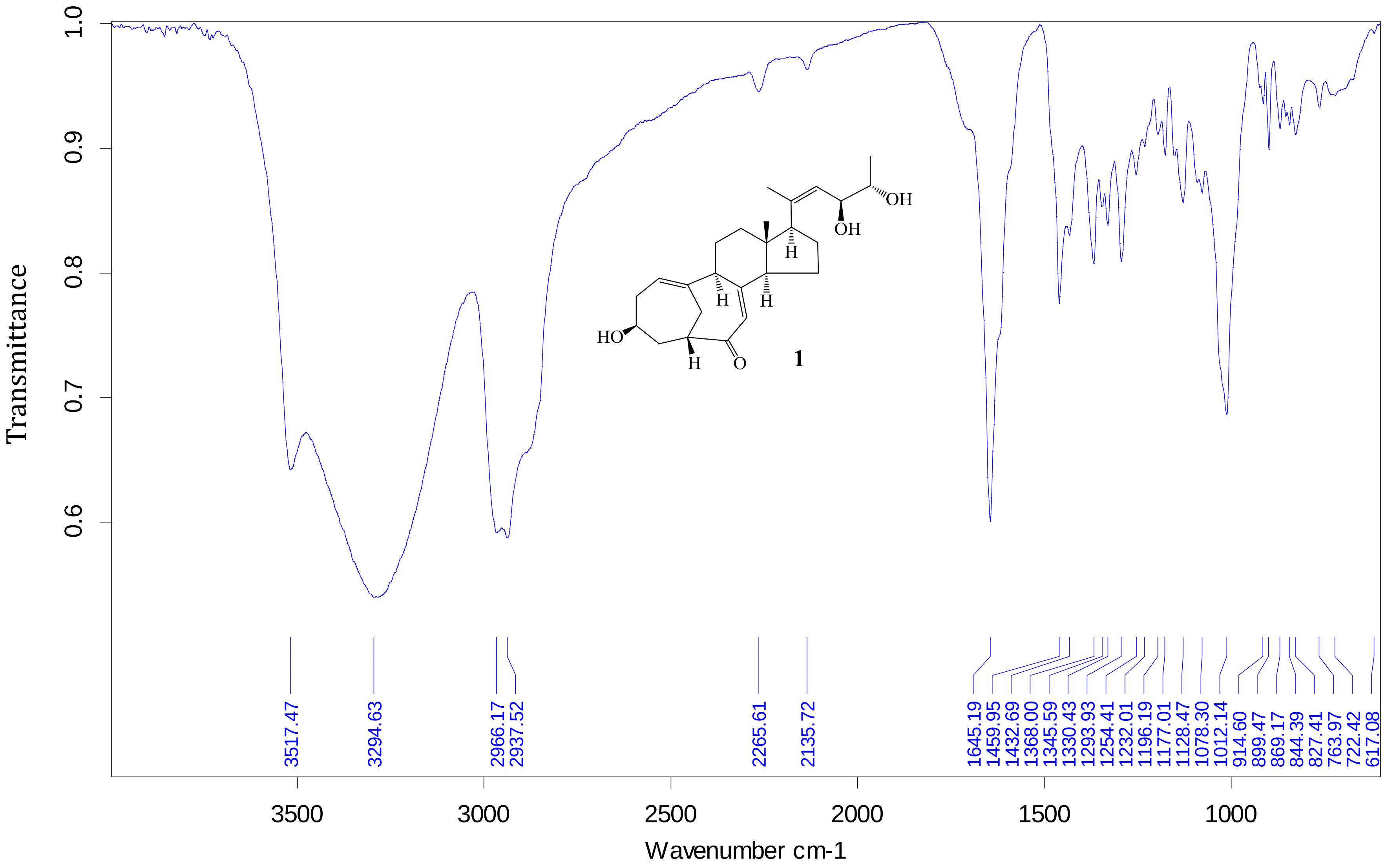

D

Figure S8. *Cont.*

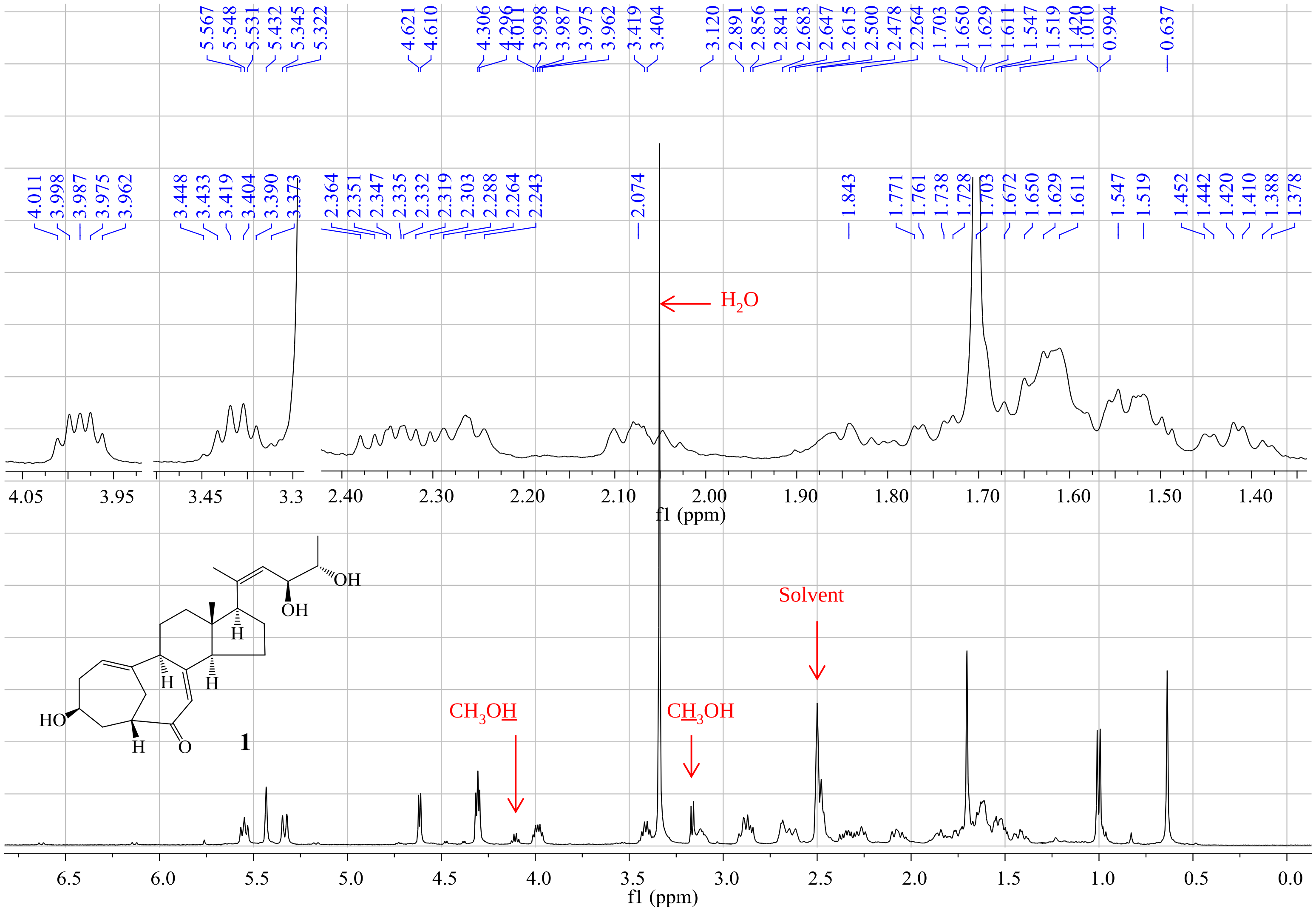

E

Figure S8. *Cont.*

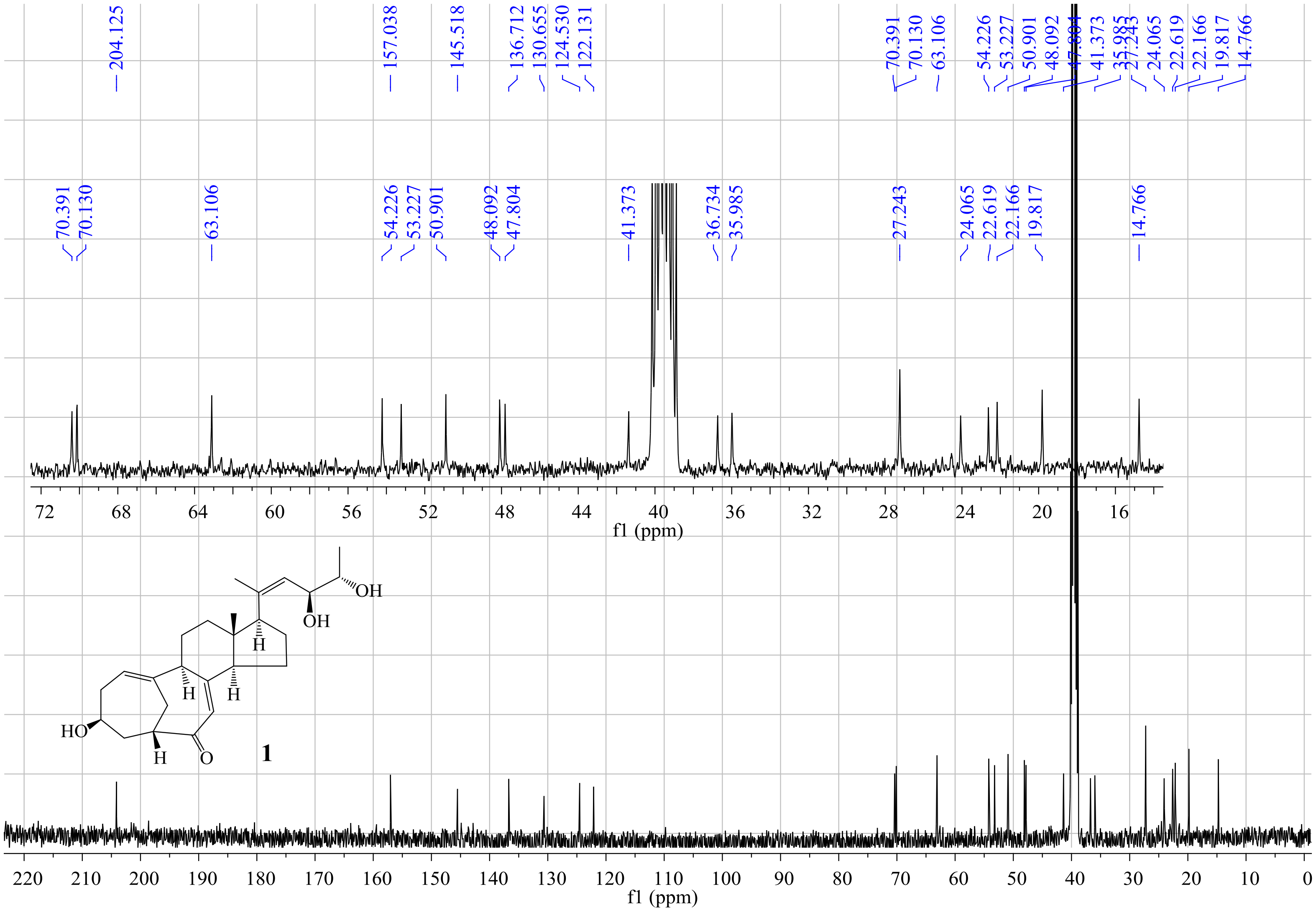

F

Figure S8. *Cont.*

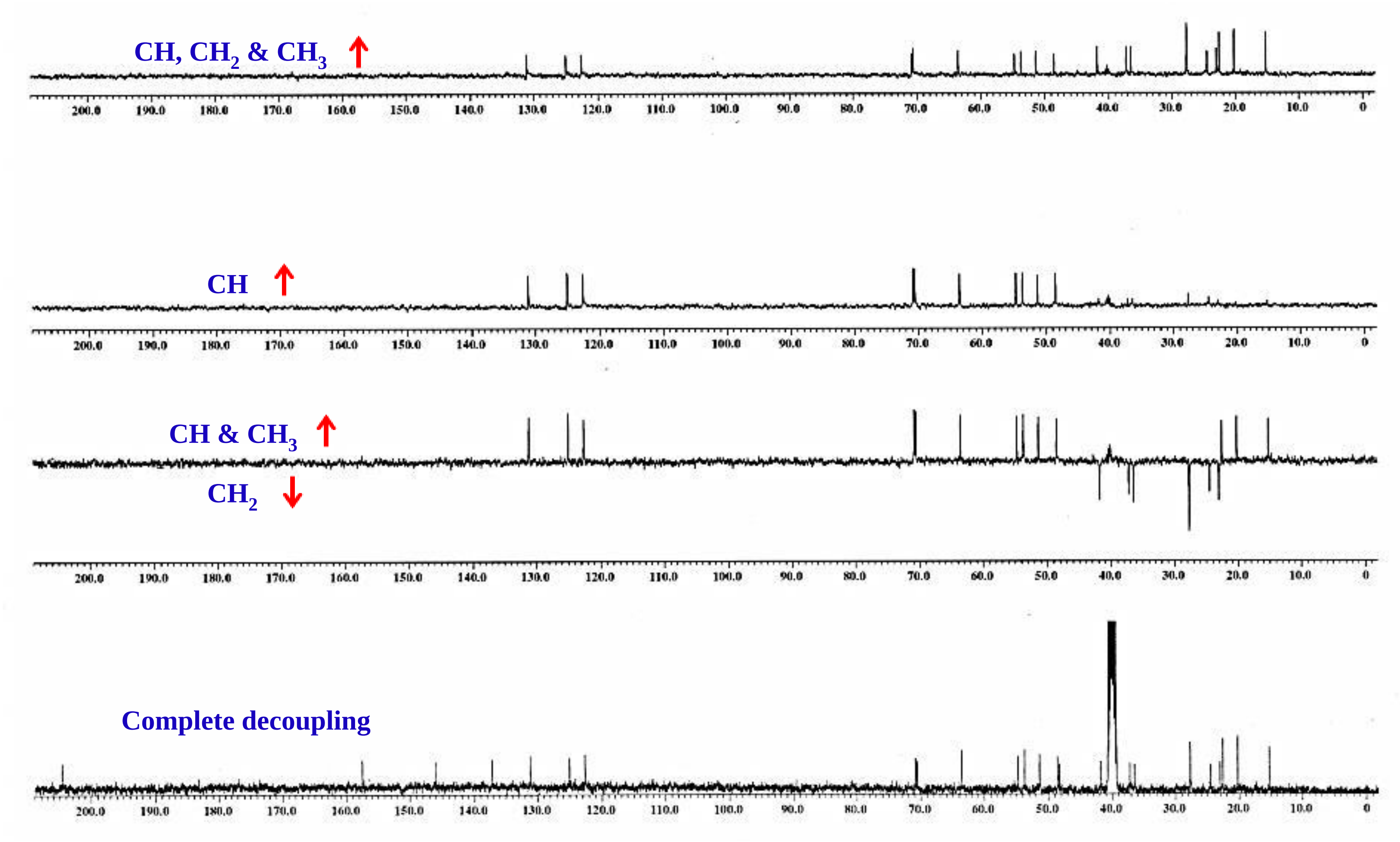

G

Figure S8. *Cont*.

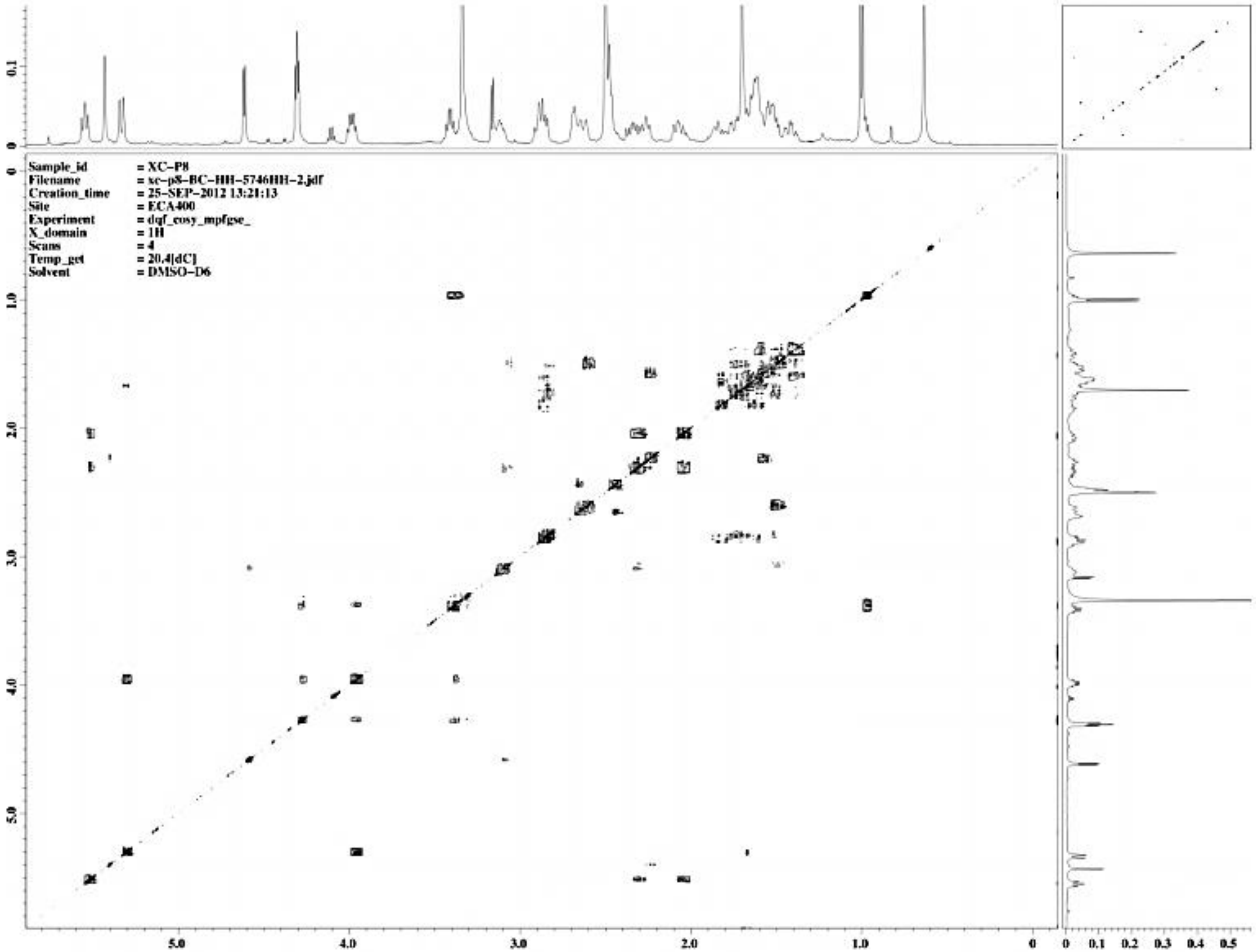

H

Figure S8. *Cont.*

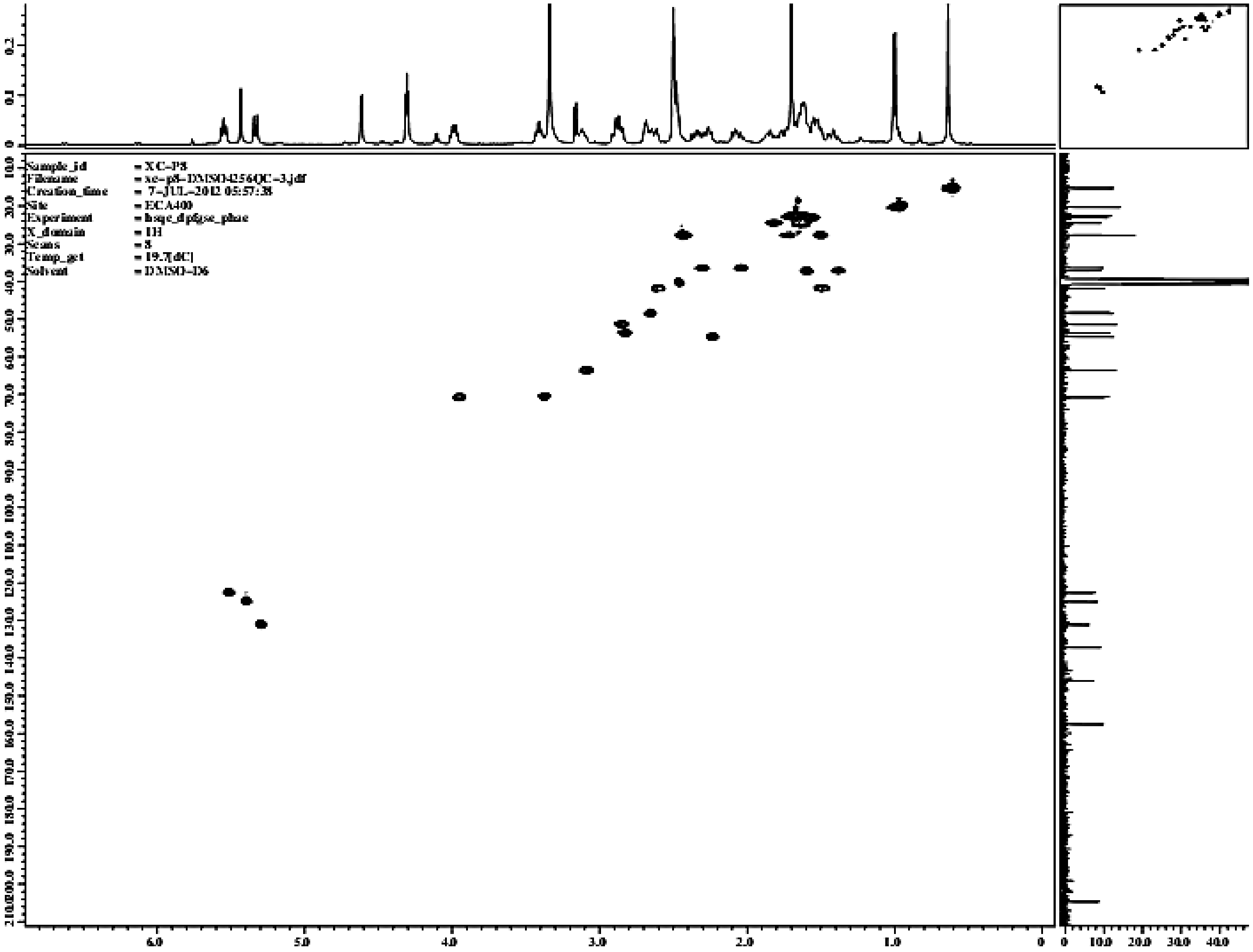

I

Figure S8. *Cont.*

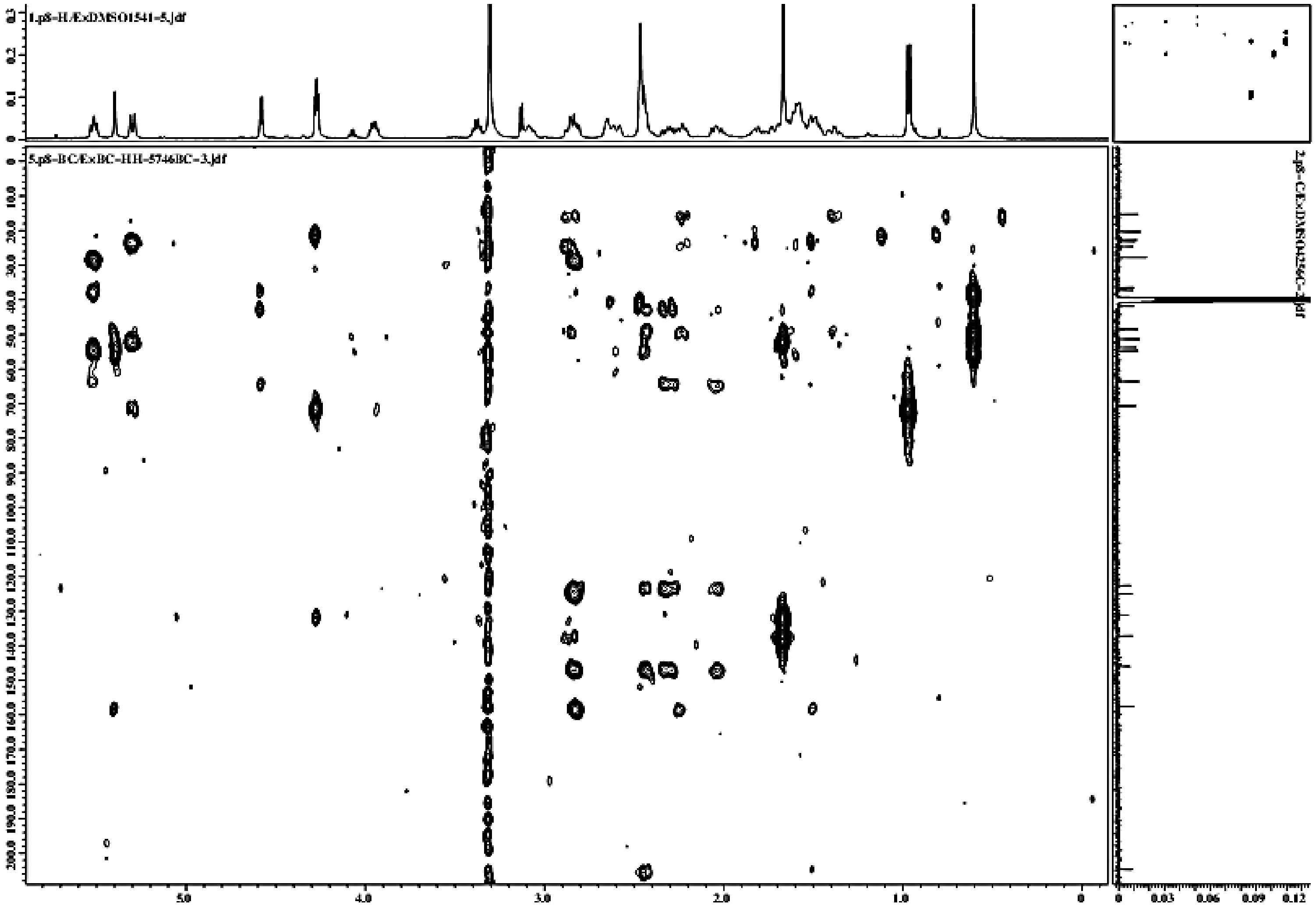

J

Figure S8. *Cont.*

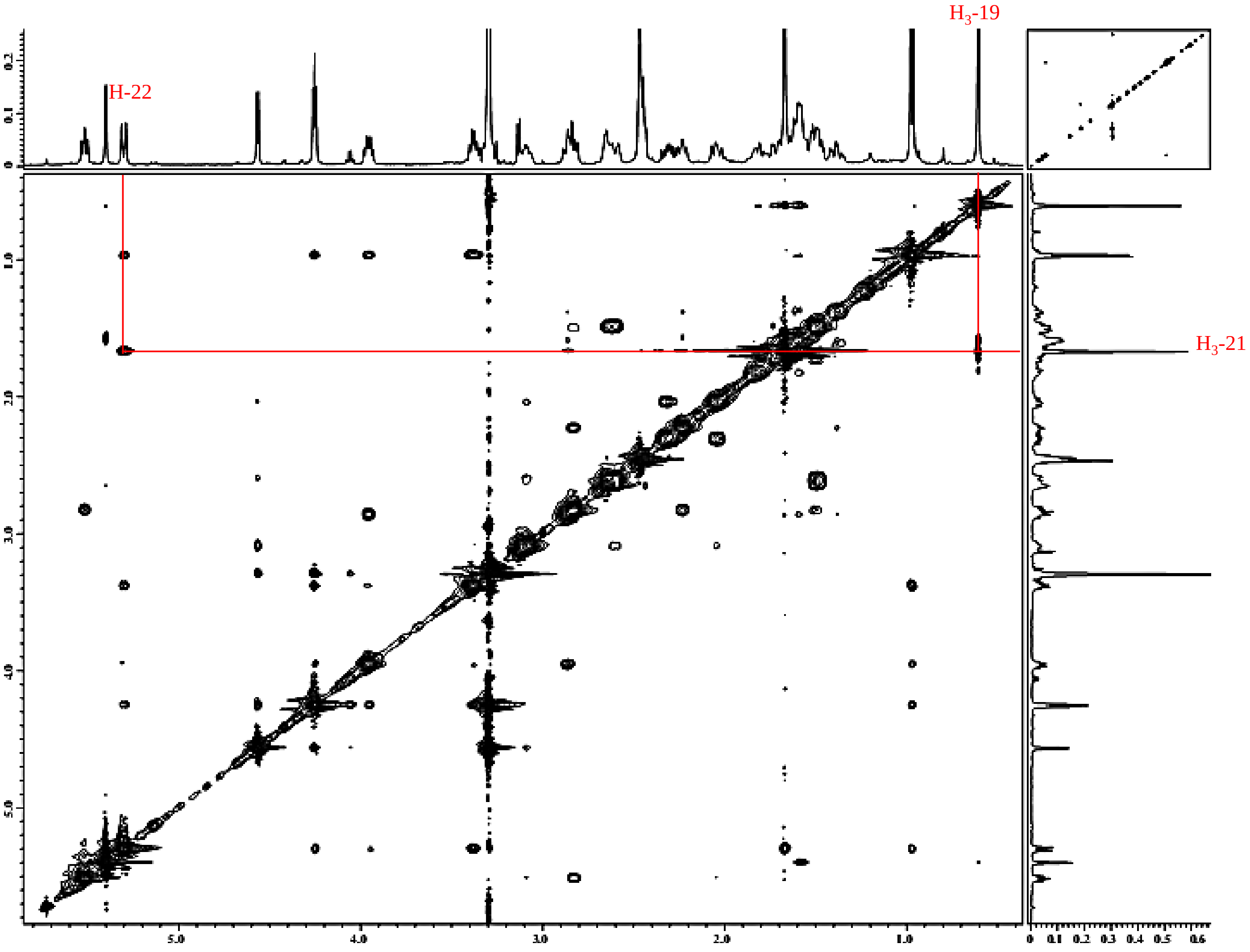

K

Figure S8. *Cont.*

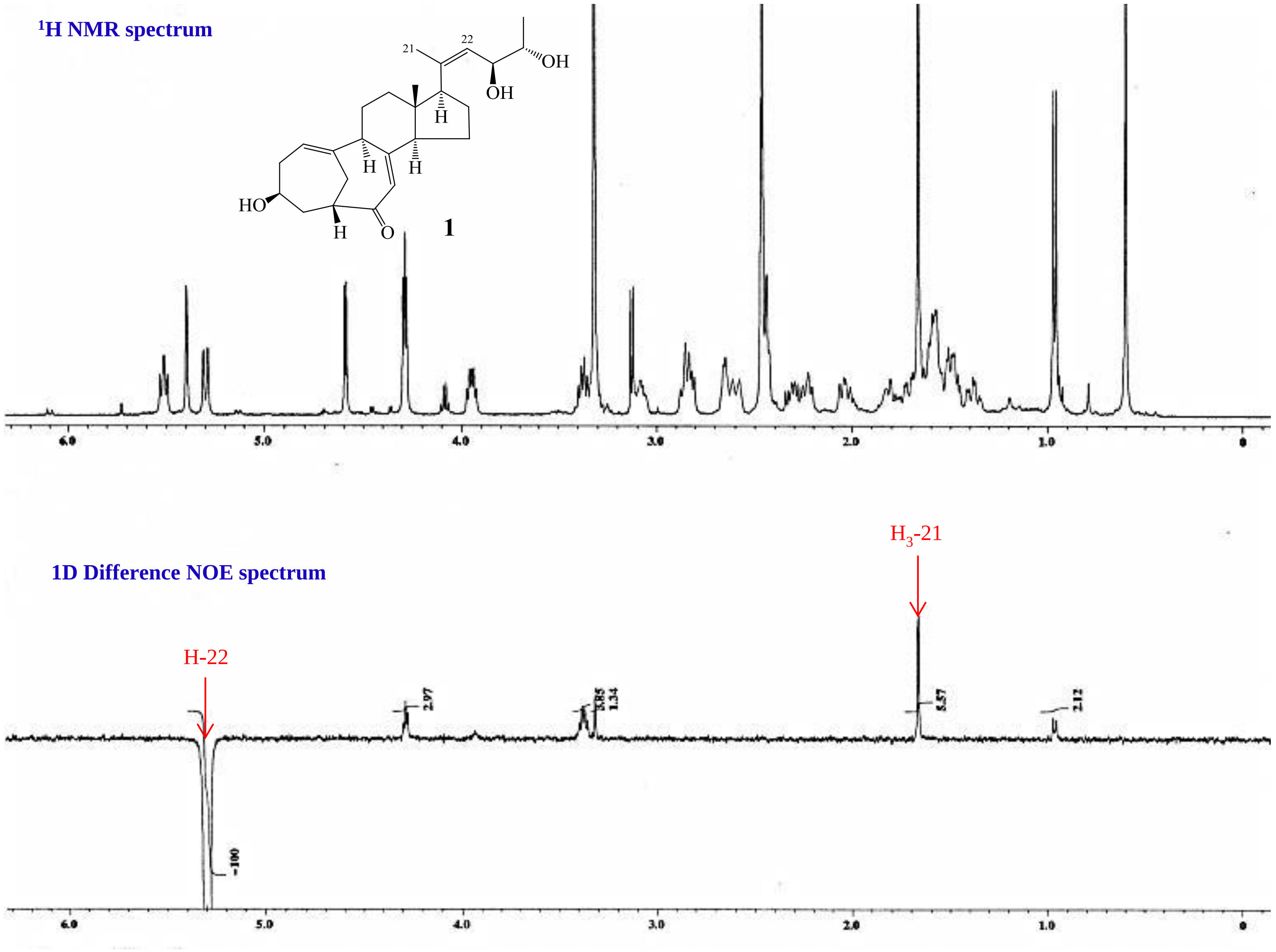

L

Figure S9. (**A**) Positive and negative ESIMS spectra of **2**. (**B**) Positive HRESIMS Spectra of **2**. (**C**) UV Spectrum of **2** in MeOH. (**D**) IR Spectrum of **2**.(**E**) 400 MHz ^{1}H NMR Spectrum of **2** in DMSO-d_6. (**F**) 100 MHz ^{13}C NMR Spectrum of **2** in DMSO-d_6. (**G**) DEPT Spectra of **2** in DMSO-d_6. (**H**) ^{1}H-^{1}H COSY Spectrum of **2** in DMSO-d_6. (**I**) HMQC Spectrum of **2** in DMSO-d_6. (**J**) HMBC Spectrum of **2** in DMSO-d_6. (**K**) NOESY Spectrum of **2** in DMSO-d_6. (**L**) 1D Difference NOE Spectrum of **2** in DMSO-d_6.

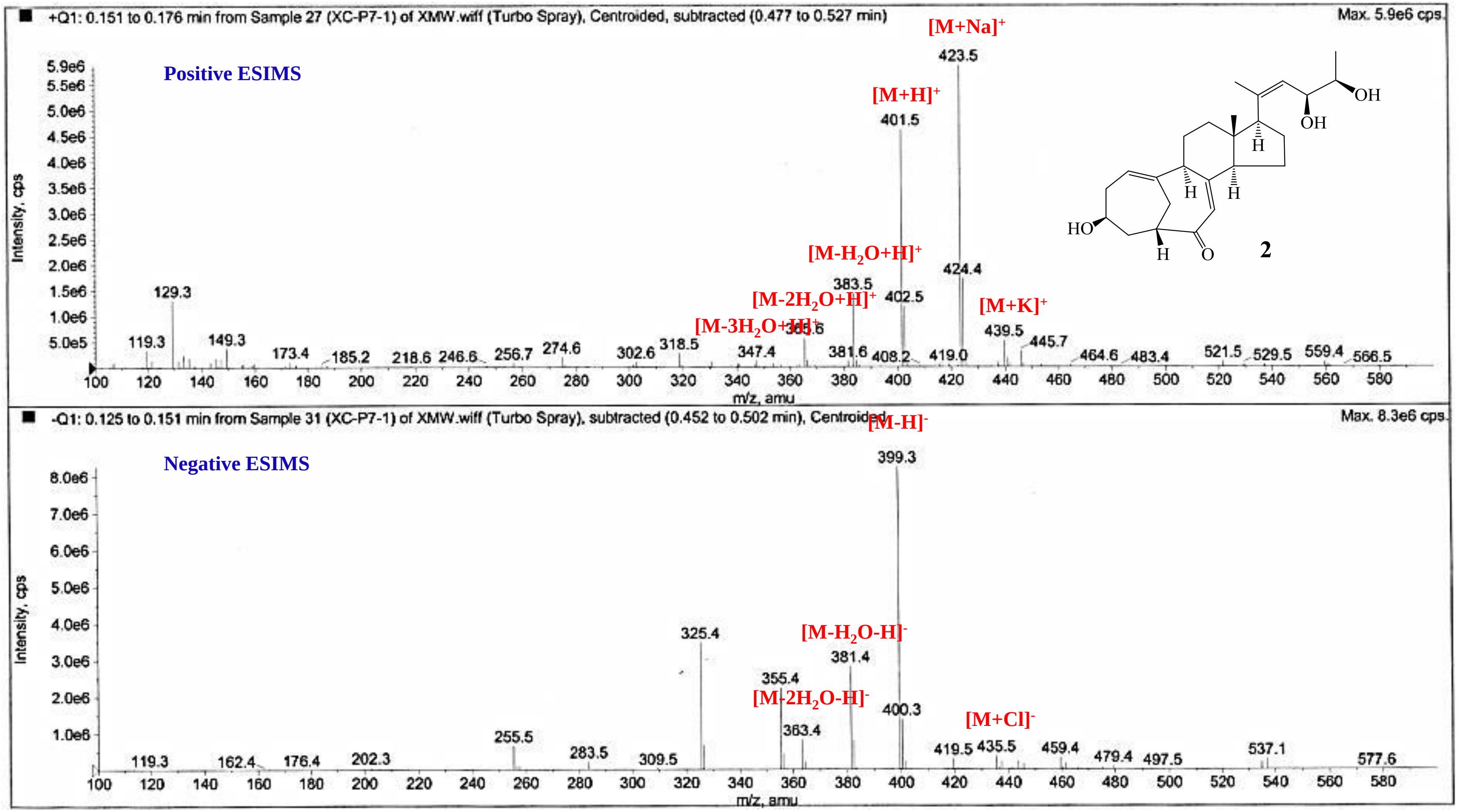

A

Figure S9. *Cont.*

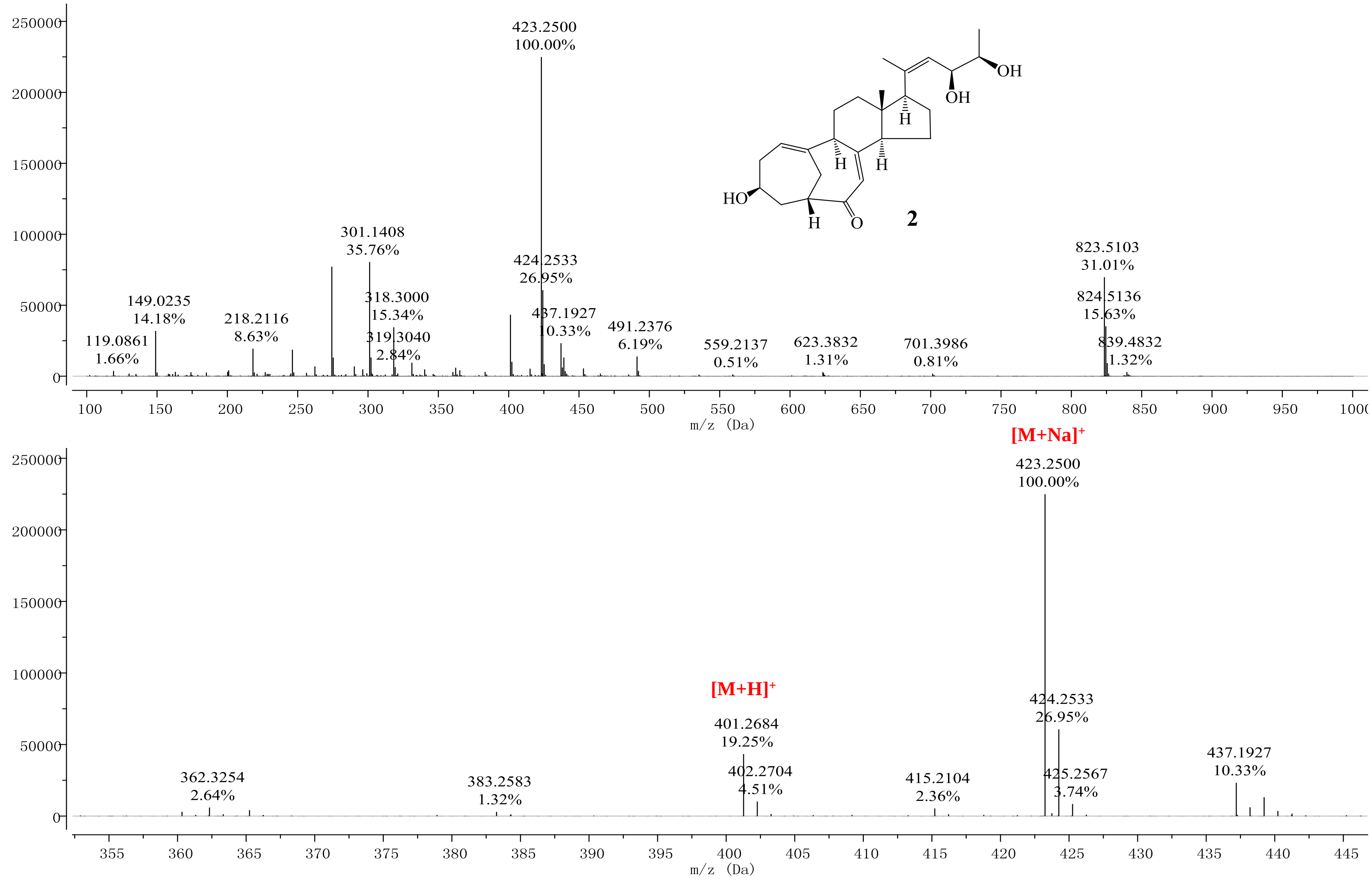

B

Figure S9. *Cont.*

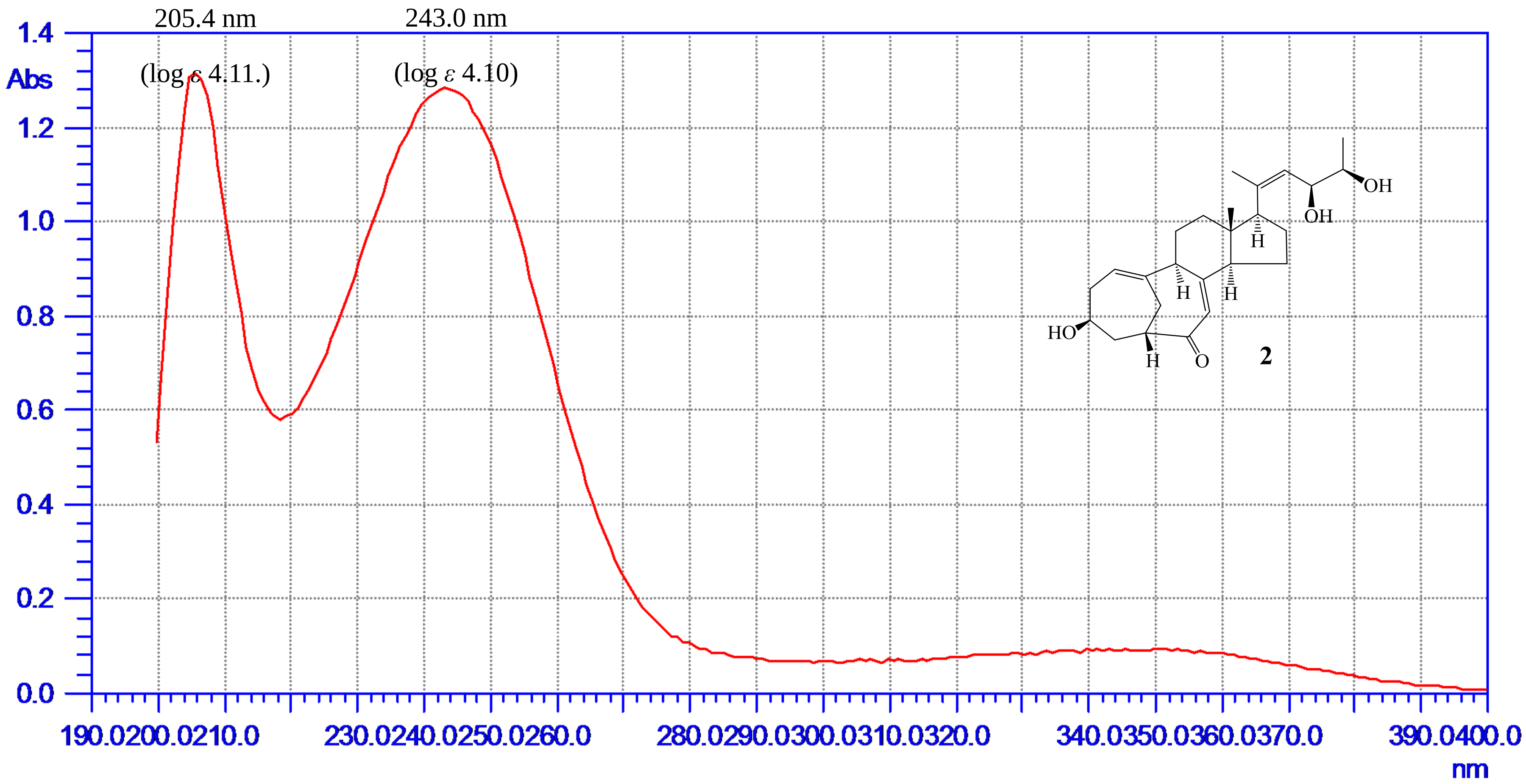

C

Figure S9. *Cont.*

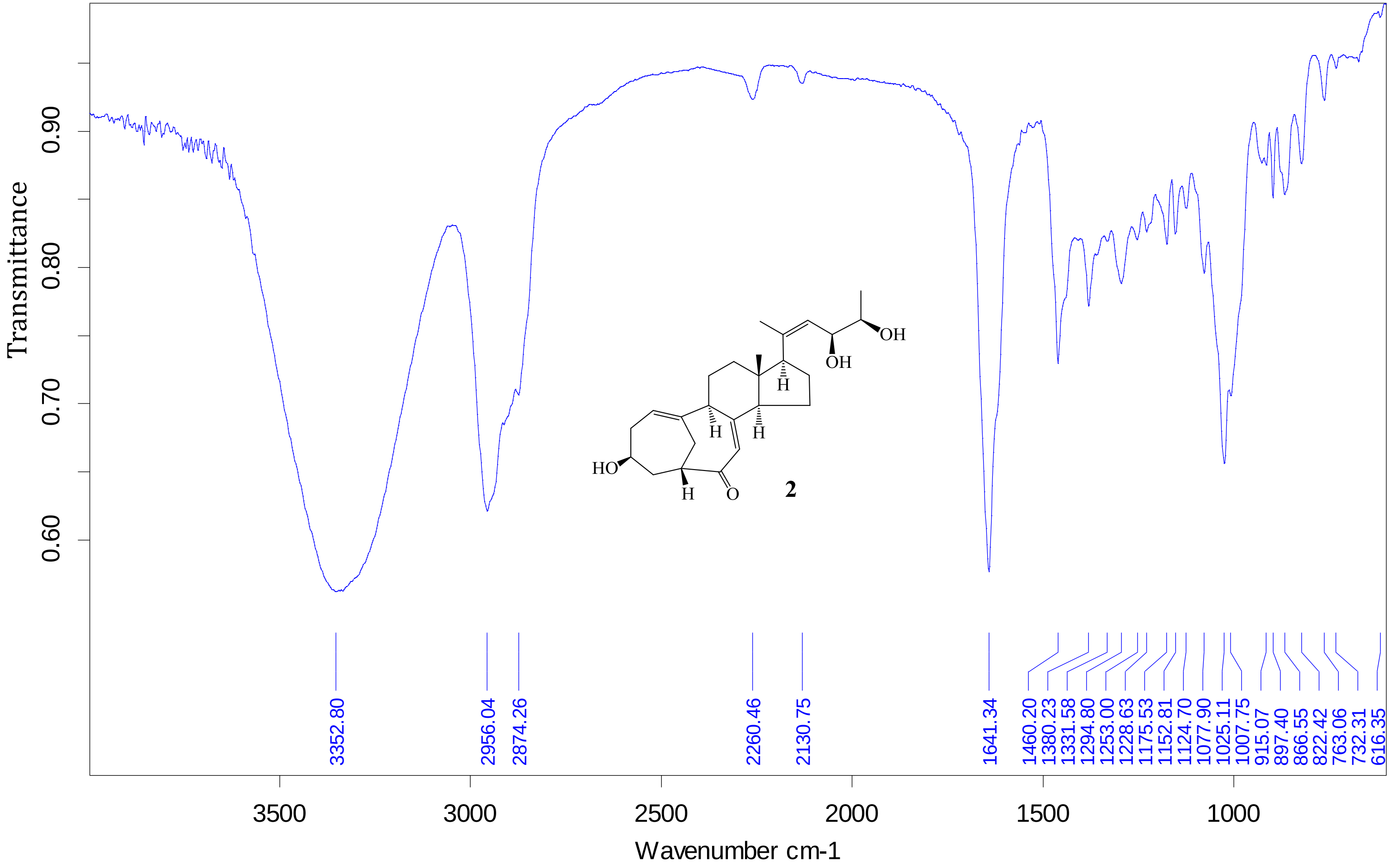

D

Figure S9. *Cont.*

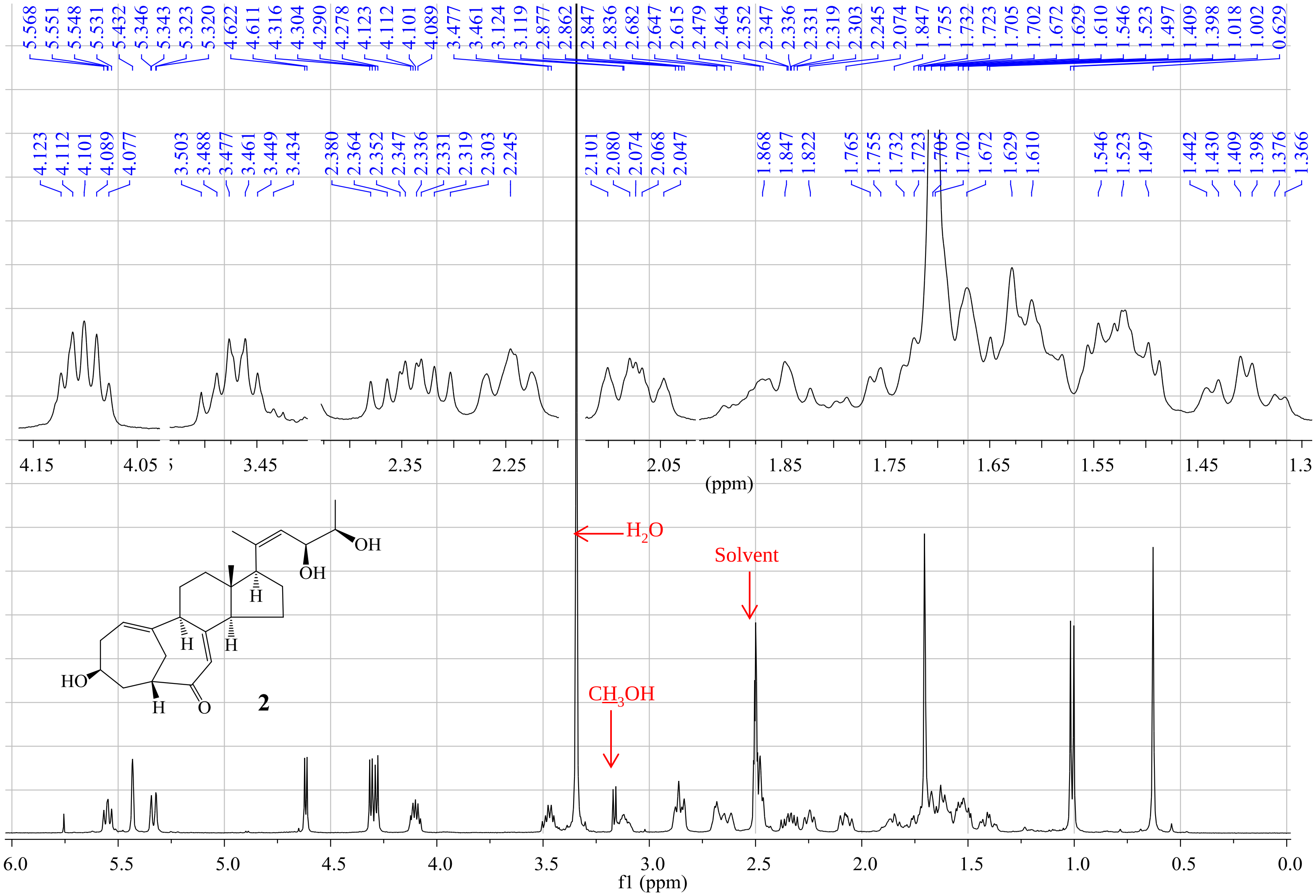

E

Figure S9. *Cont.*

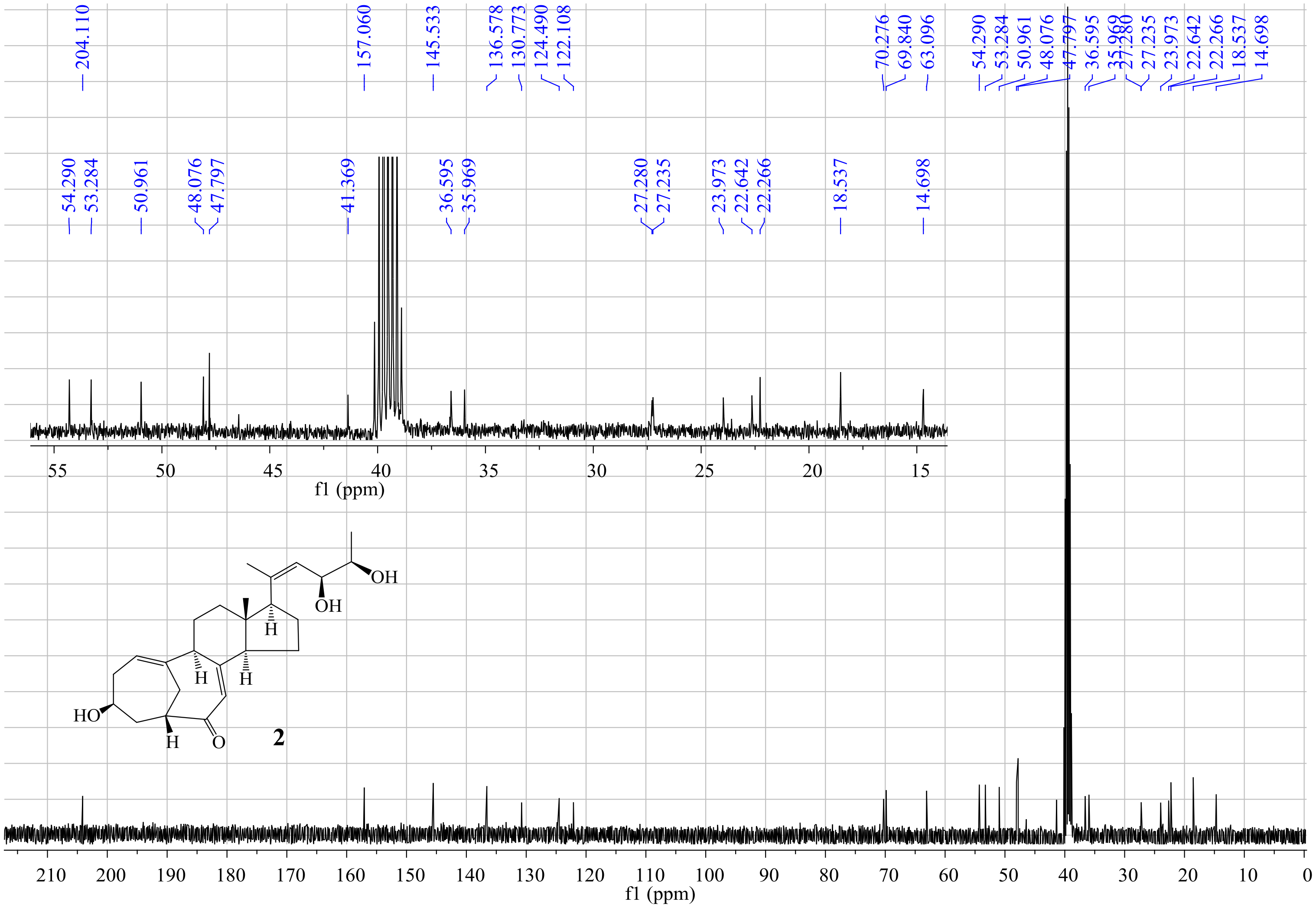

F

Figure S9. *Cont.*

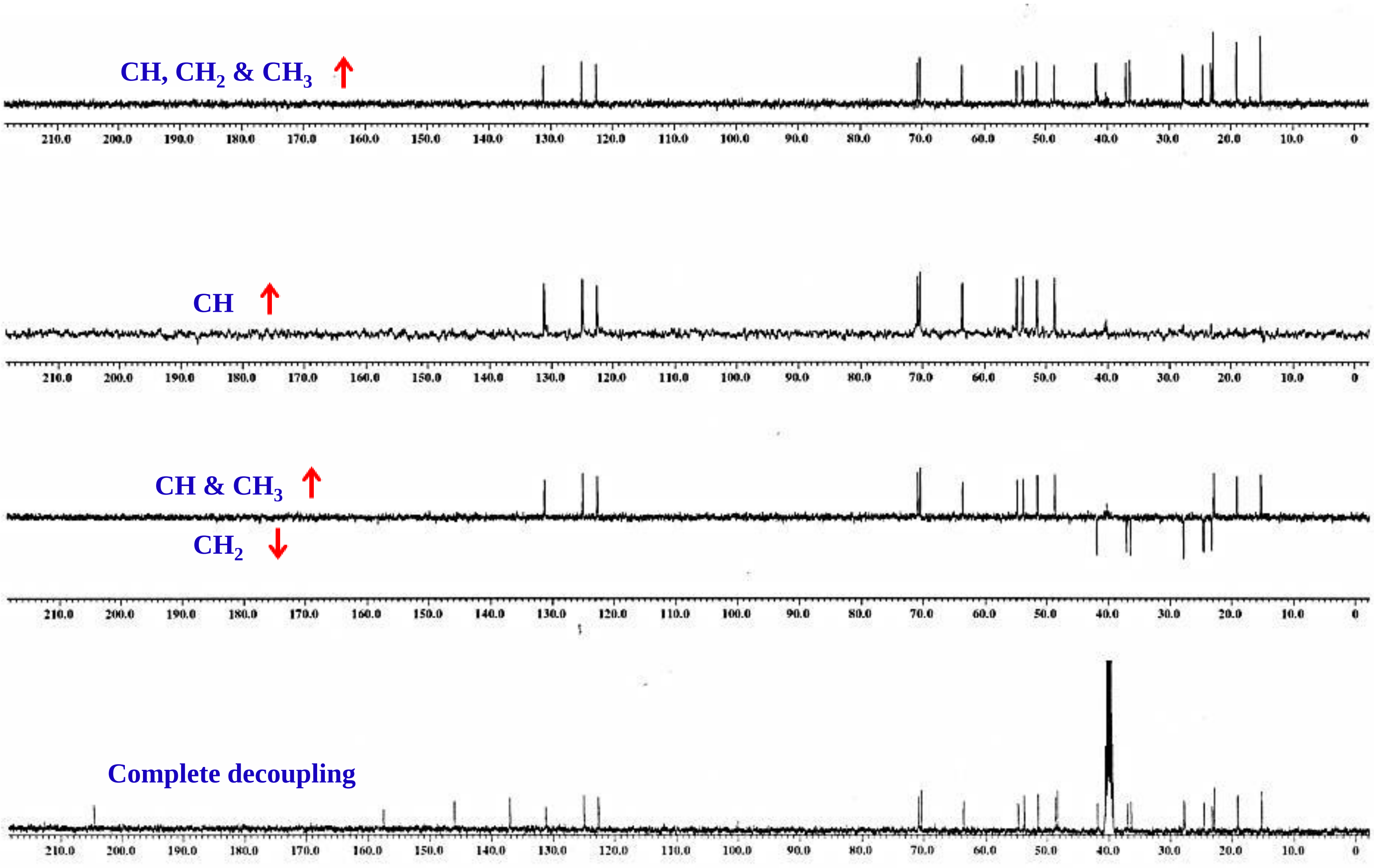

G

Figure S9. *Cont.*

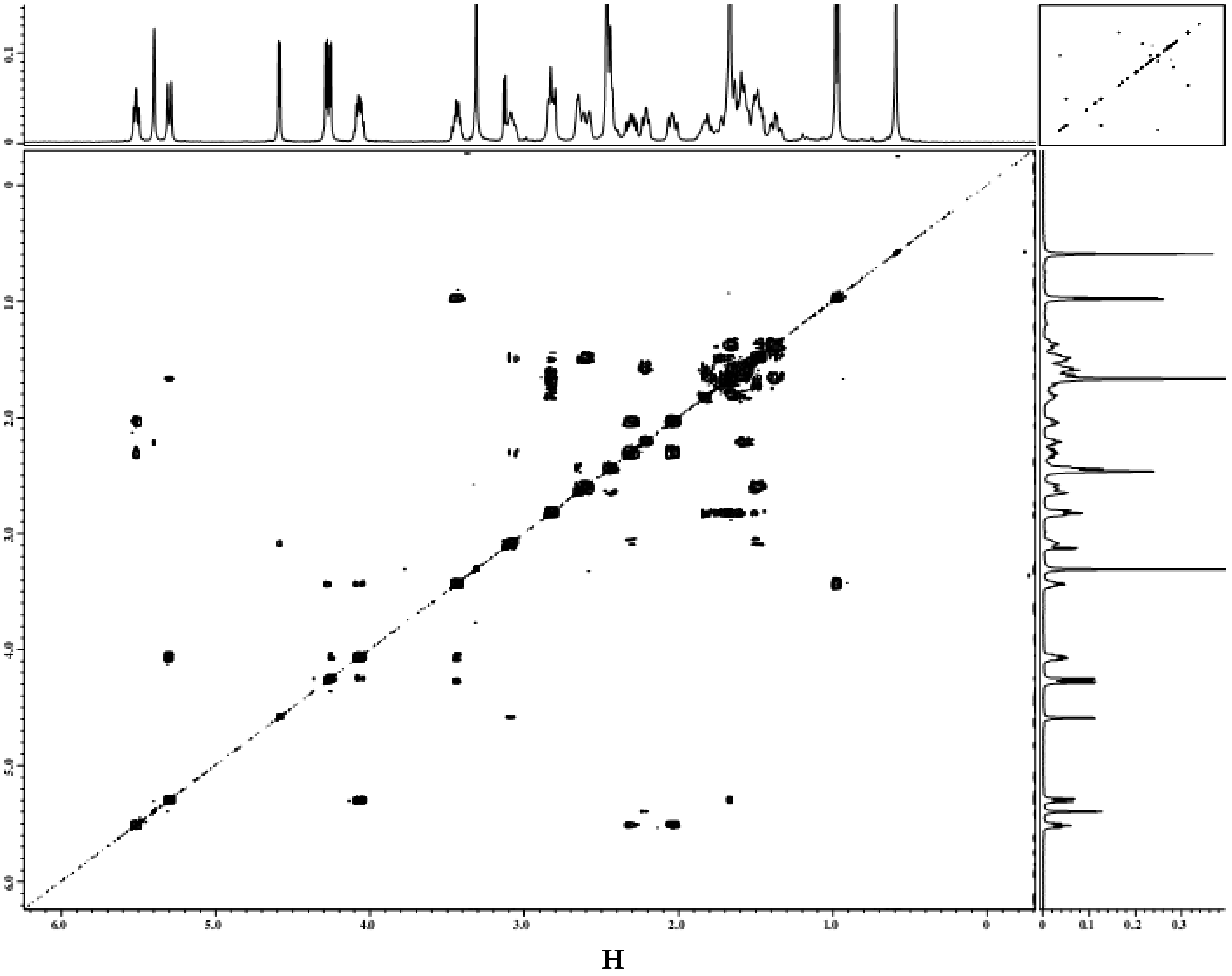

Figure S9. *Cont.*

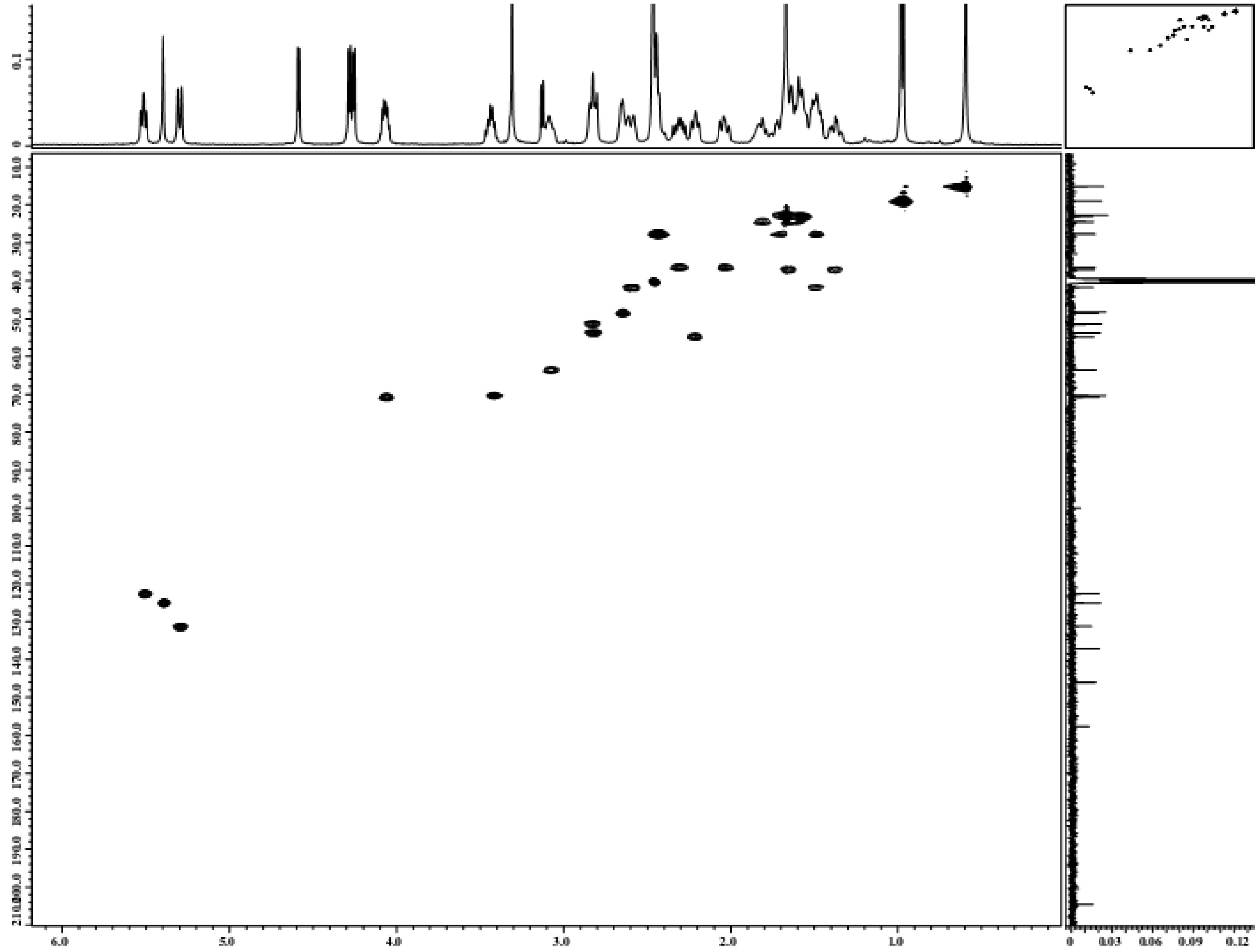

I

Figure S9. *Cont.*

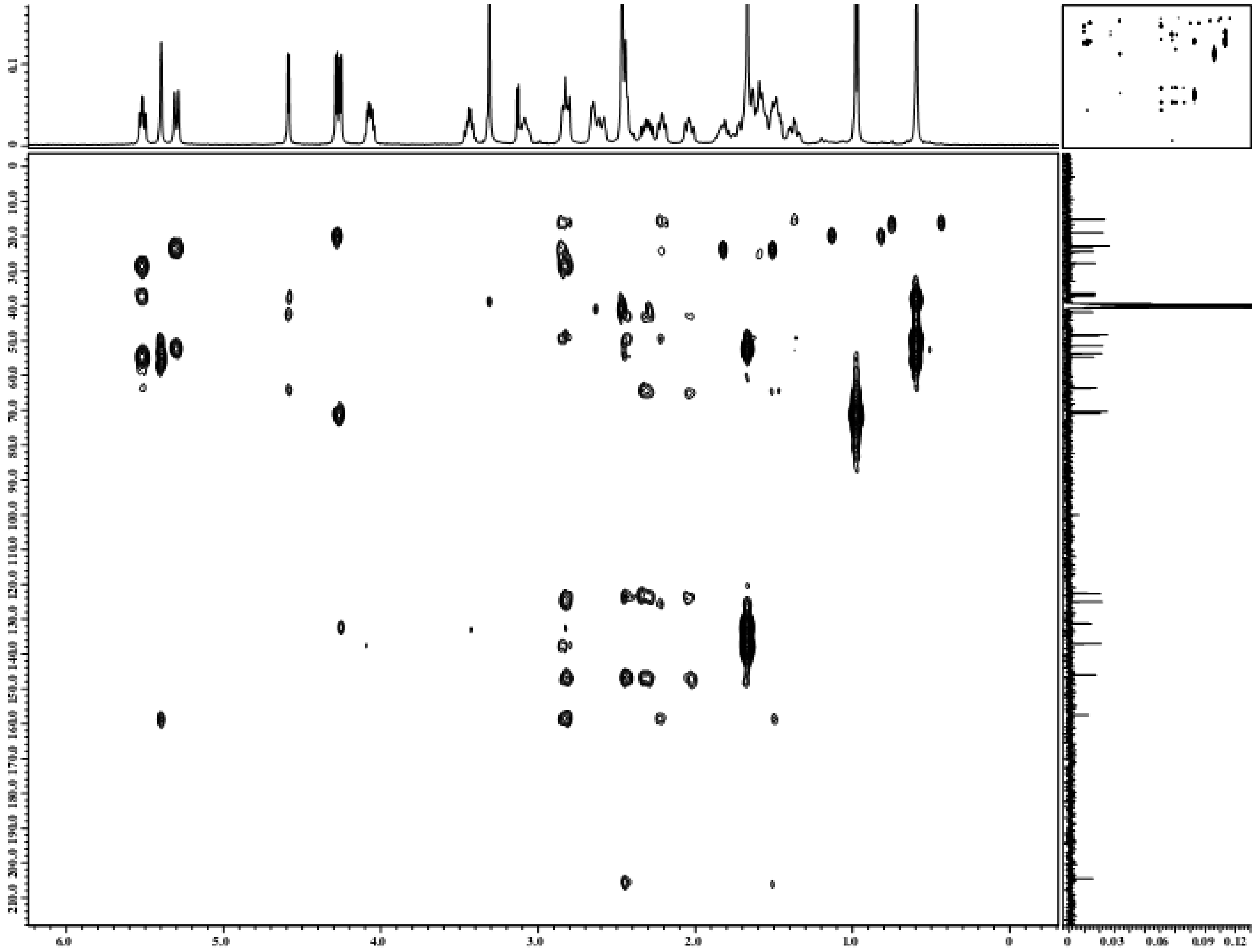

J

Figure S9. *Cont.*

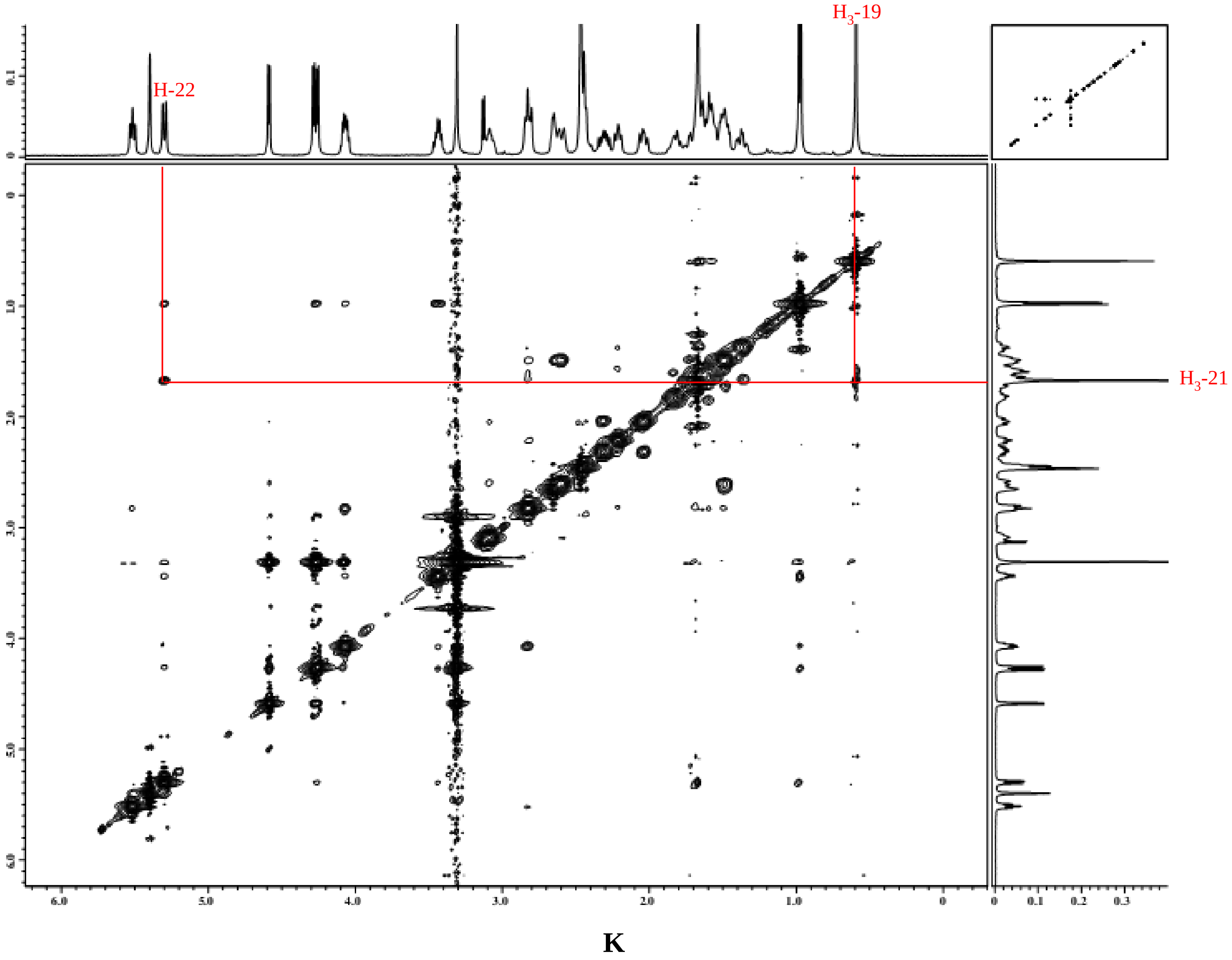

Figure S9. *Cont.*

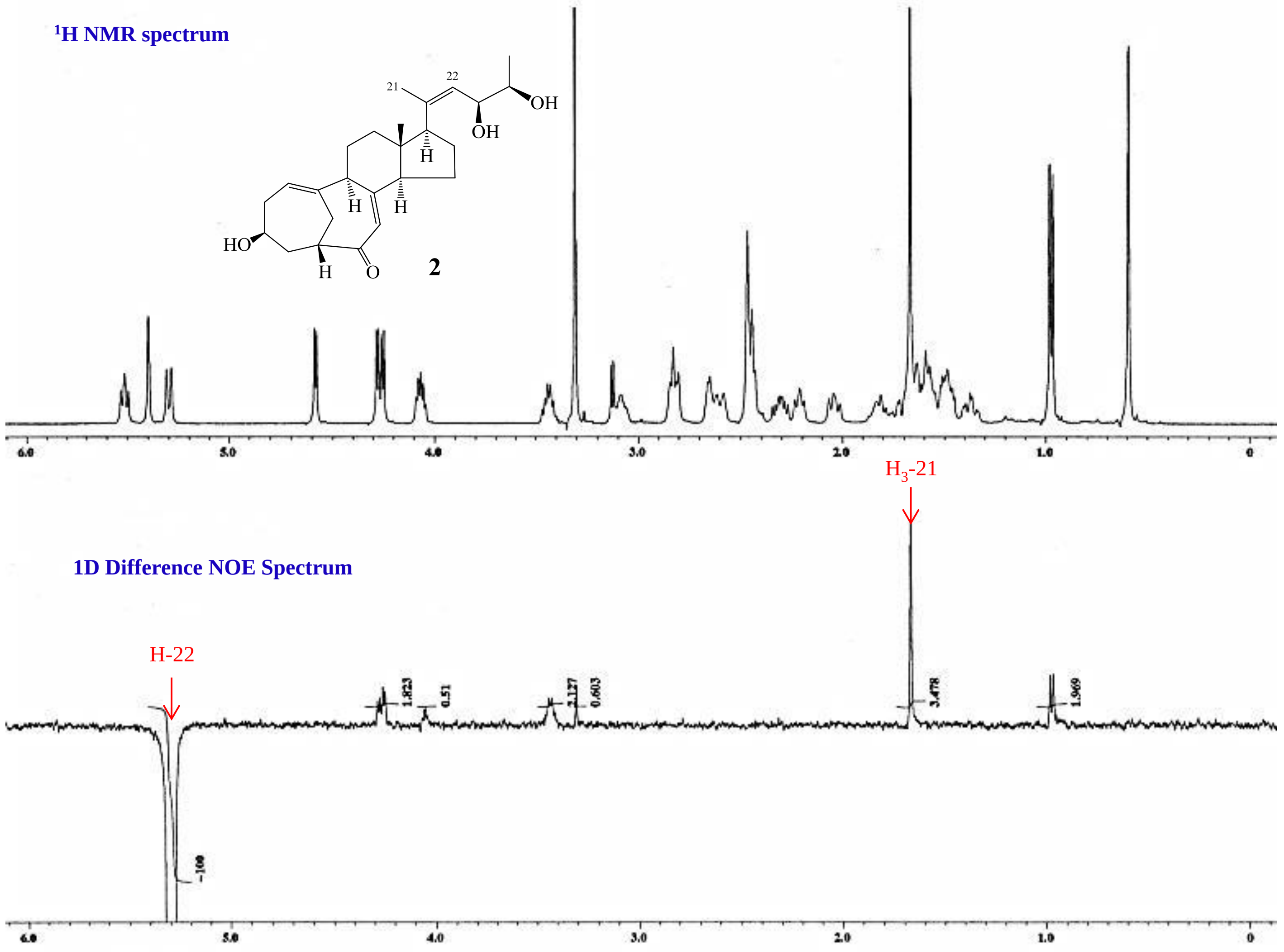

L

Figure S10. (**A**) Positive and negative ESIMS spectra of **3**. (**B**) HRESIMS Spectra of **3**. (**C**) UV Spectrum of **3** in MeOH. (**D**) IR Spectrum of **3**. (**E**) 400 MHz ^{1}H NMR Spectrum of **3** in DMSO-d_6. (**F**) 100 MHz ^{13}C NMR Spectrum of **3** in DMSO-d_6. (**G**) DEPT Spectra of **3** in DMSO-d_6. (**H**) ^{1}H-^{1}H COSY Spectrum of **2** in DMSO-d_6. (**I**) HMQC Spectrum of **3** in DMSO-d_6. (**J**) HMBC Spectrum of **3** in DMSO-d_6. (**K**) NOESY Spectrum of **3** in DMSO-d_6. (**L**) 1D Difference NOE Spectrum of **3** in DMSO-d_6.

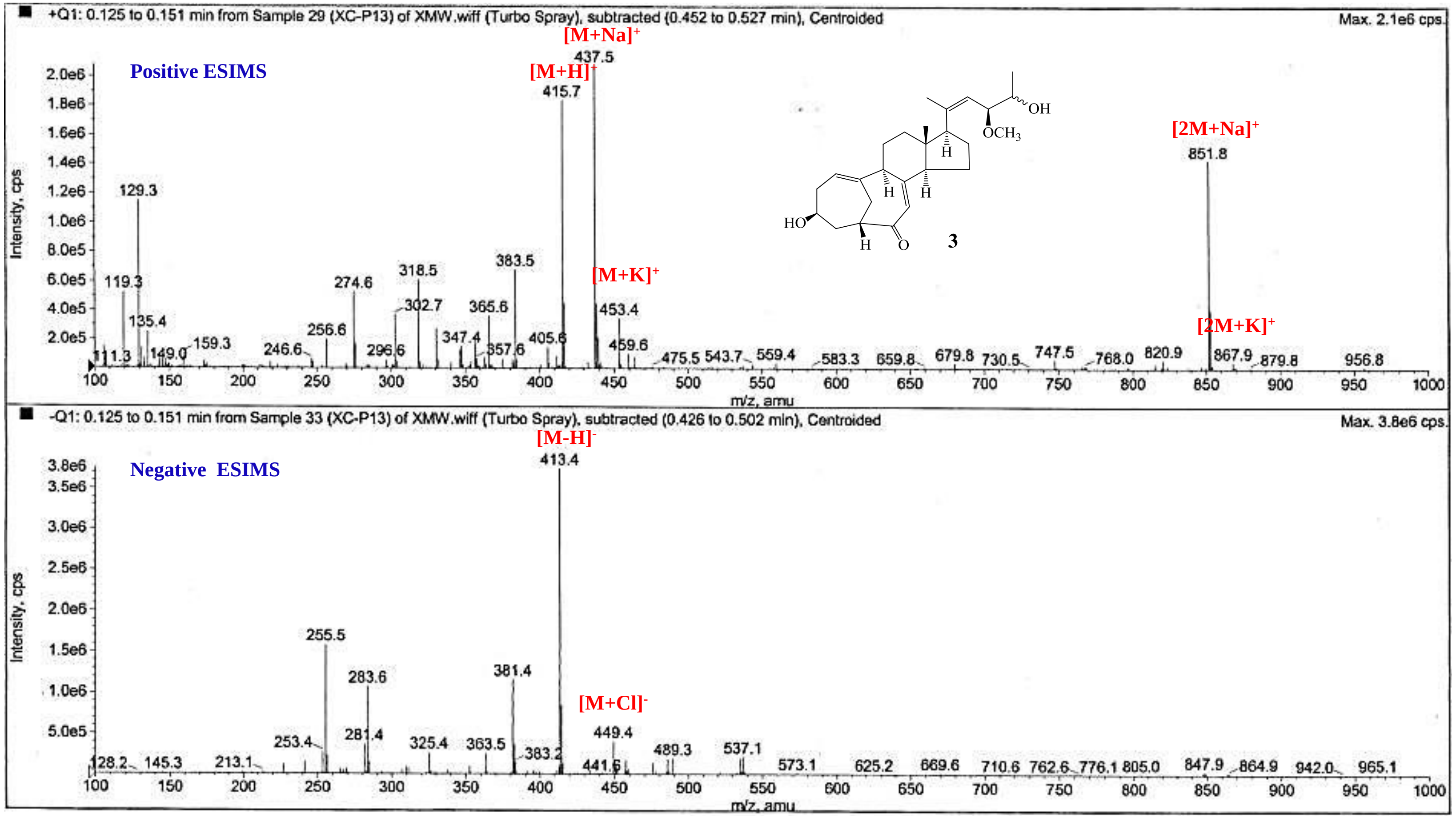

A

Figure S10. *Cont.*

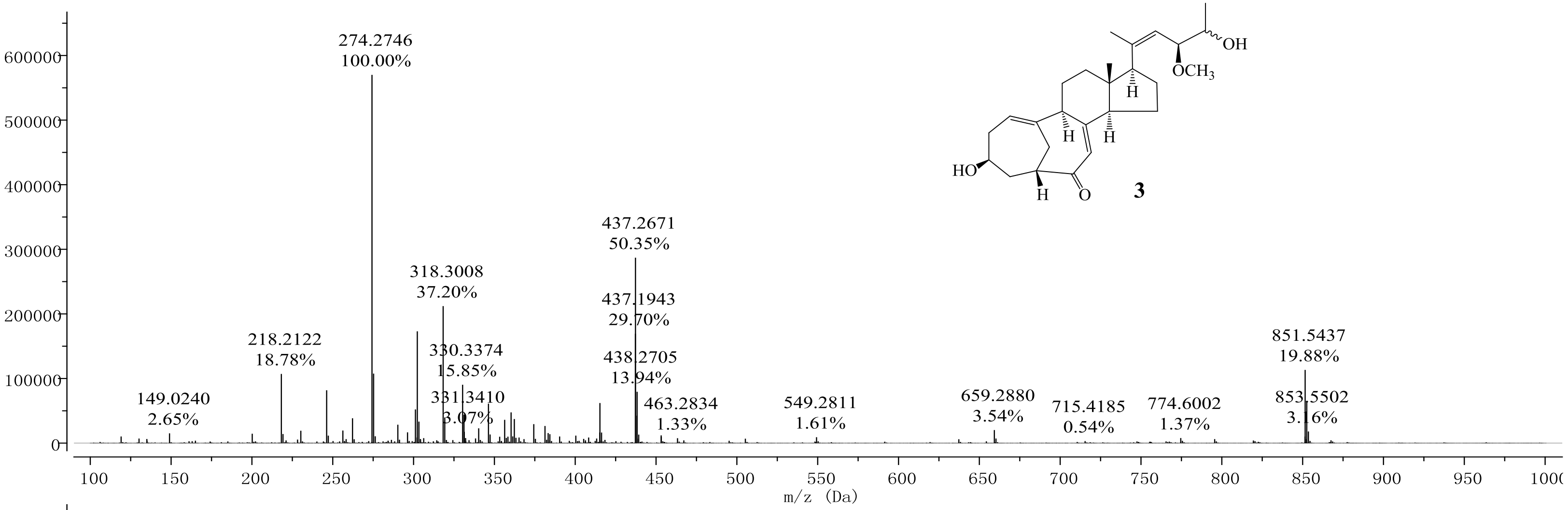

3

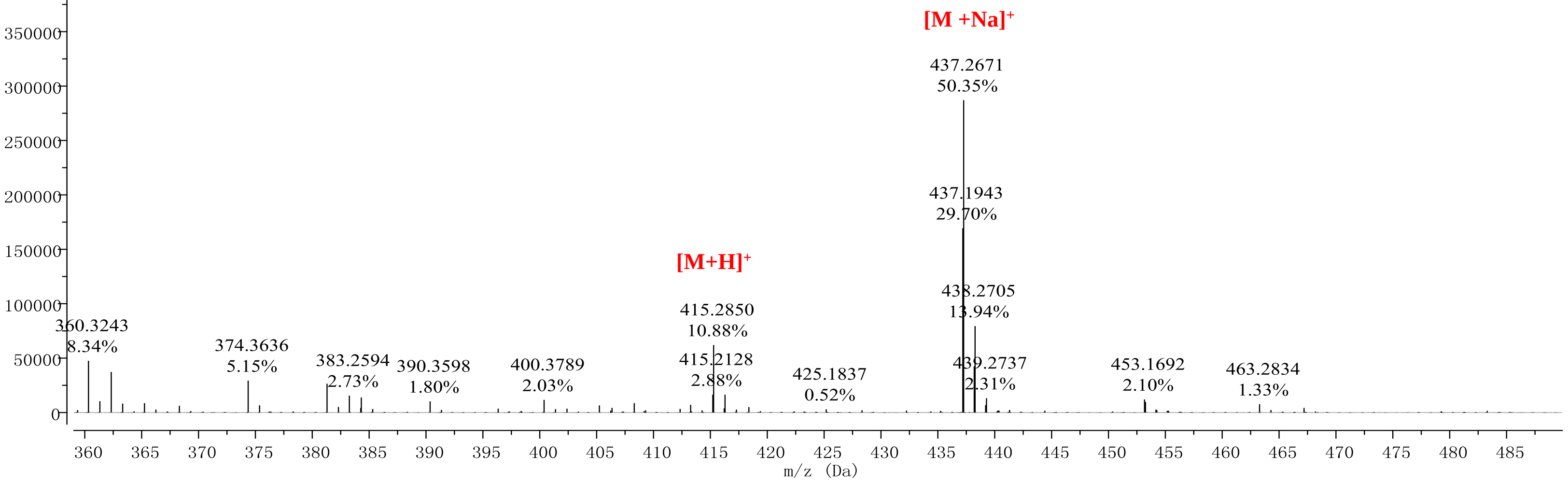

B

Figure S10. *Cont.*

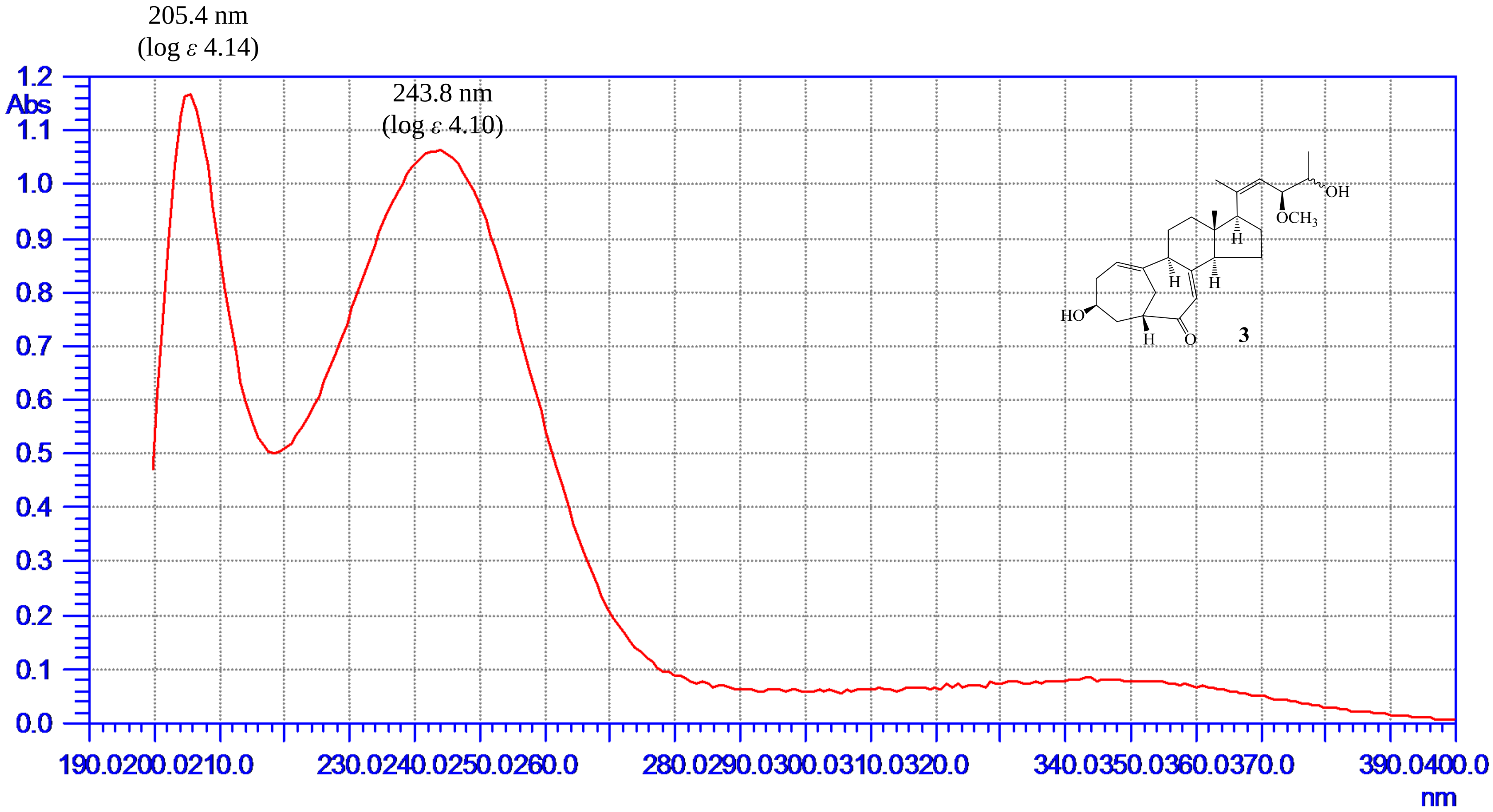

C

Figure S10. *Cont.*

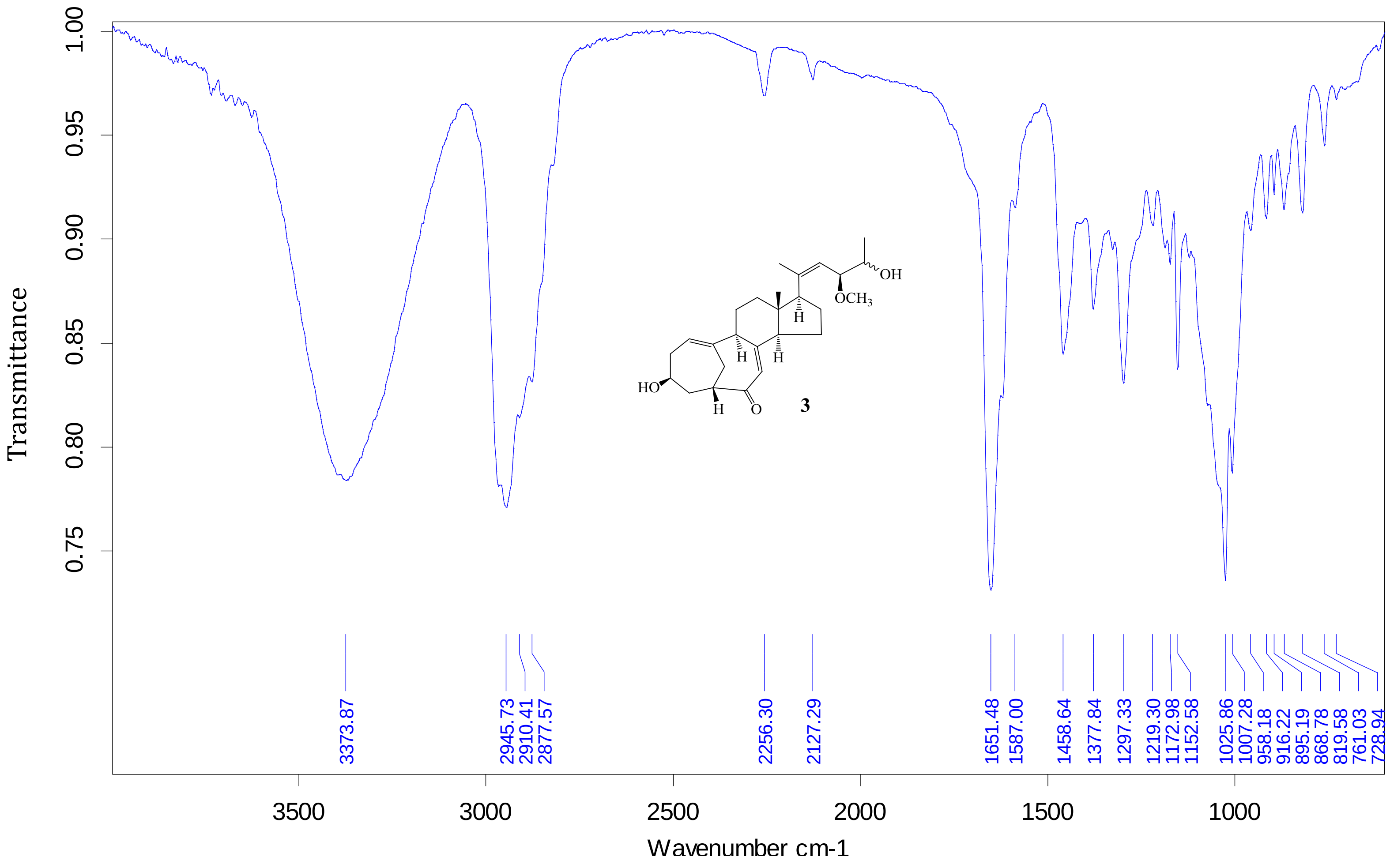

D

Figure S10. *Cont.*

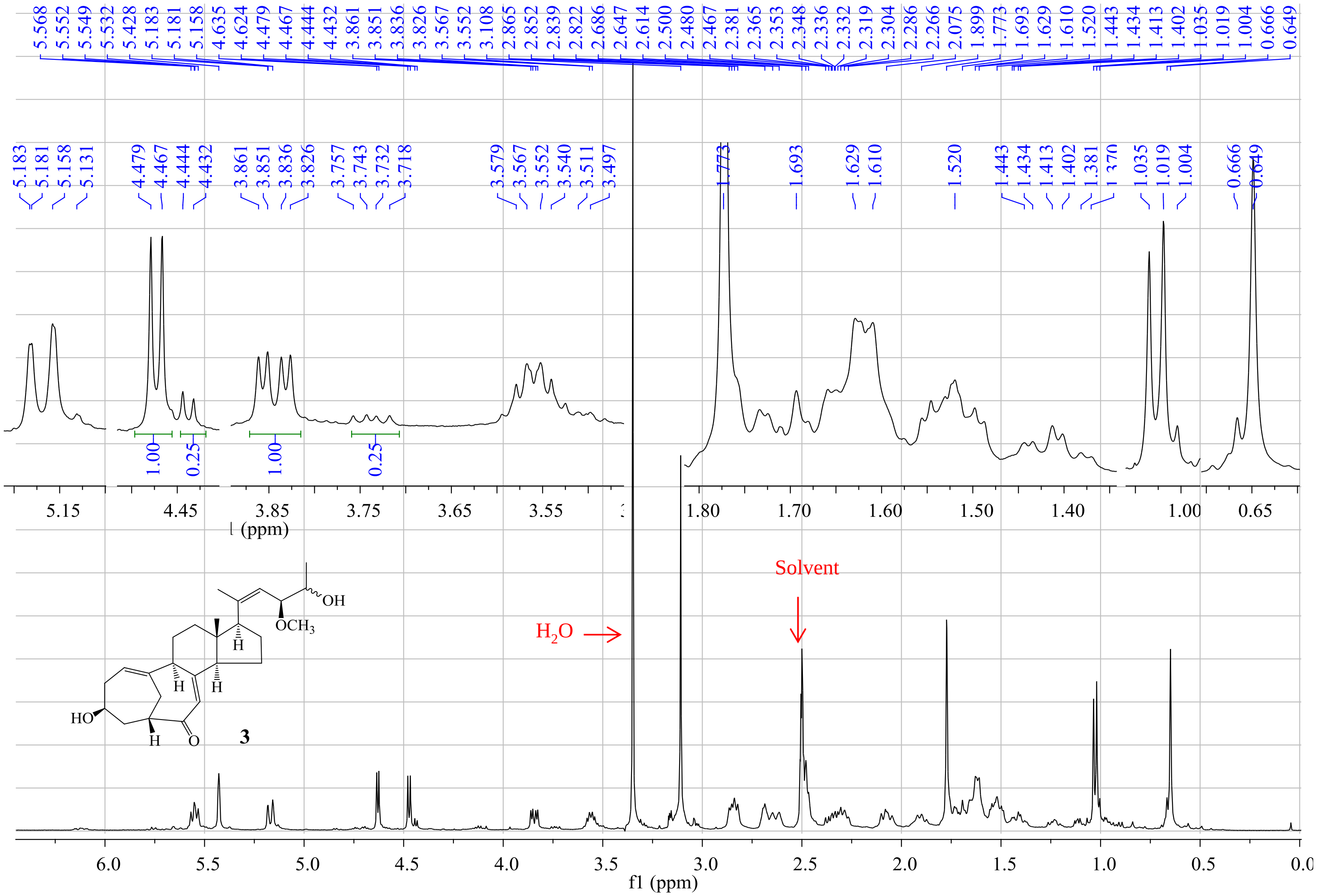

E

Figure S10. *Cont.*

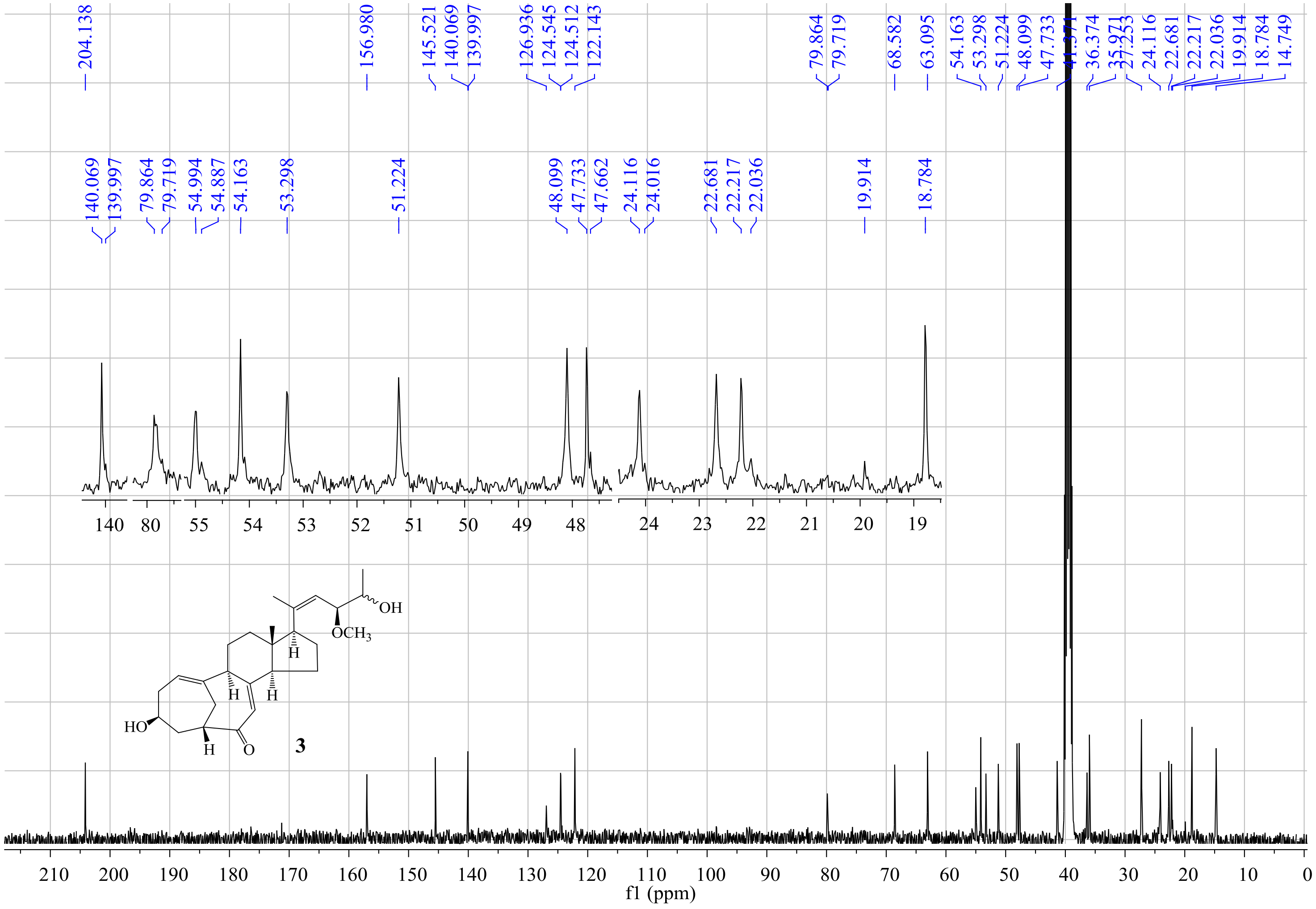

F

Figure S10. *Cont.*

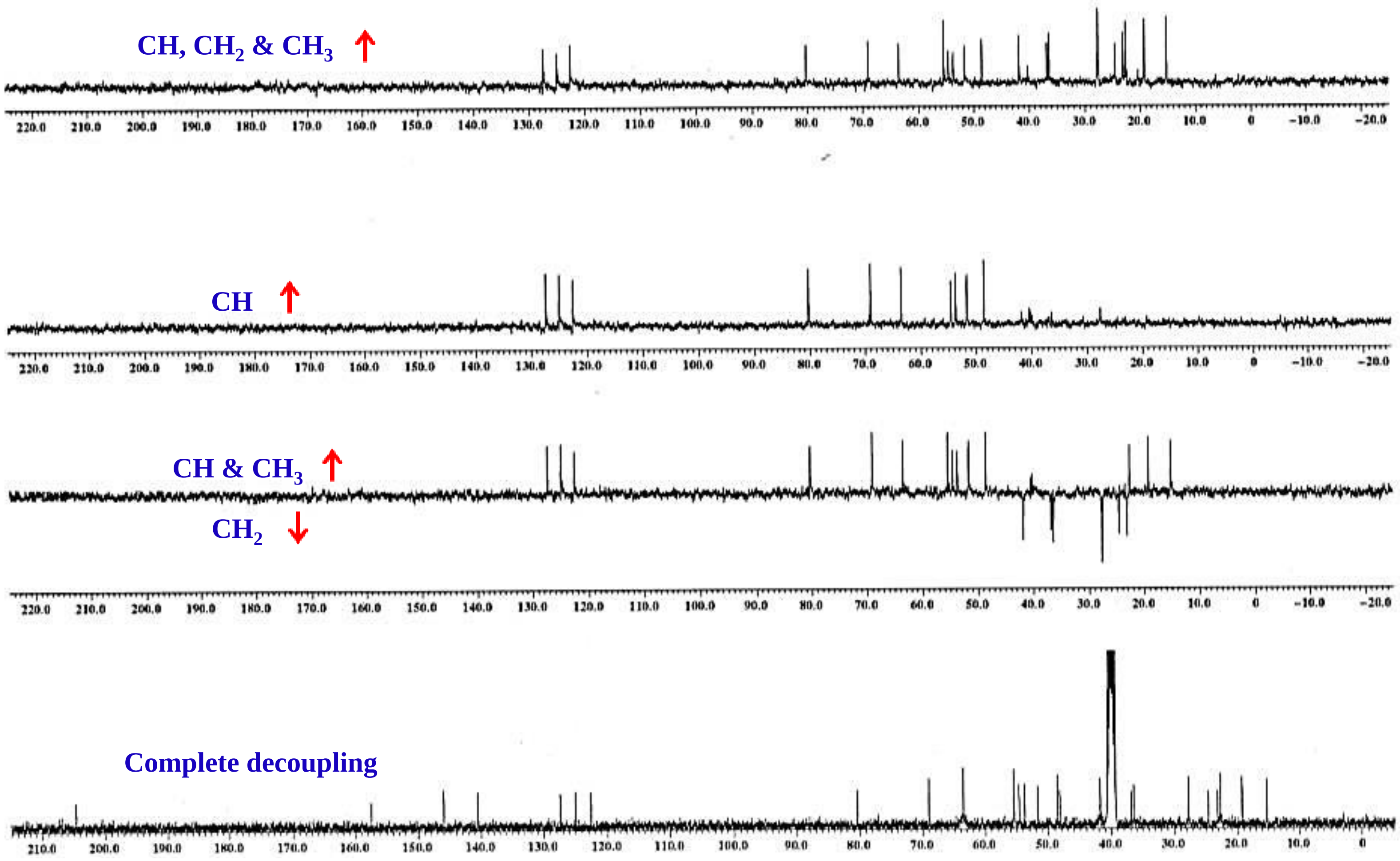

G

Figure S10. *Cont.*

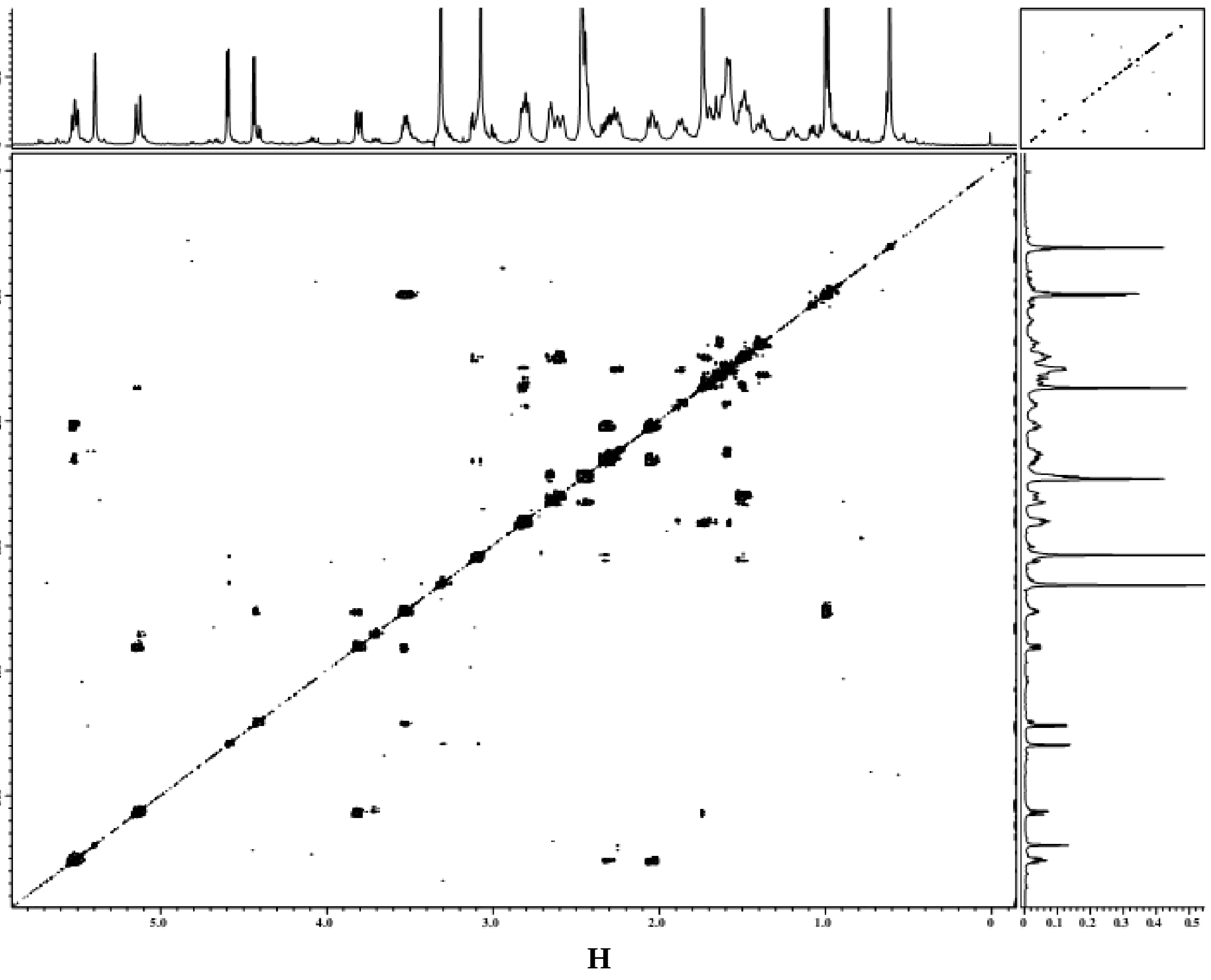

Figure S10. *Cont.*

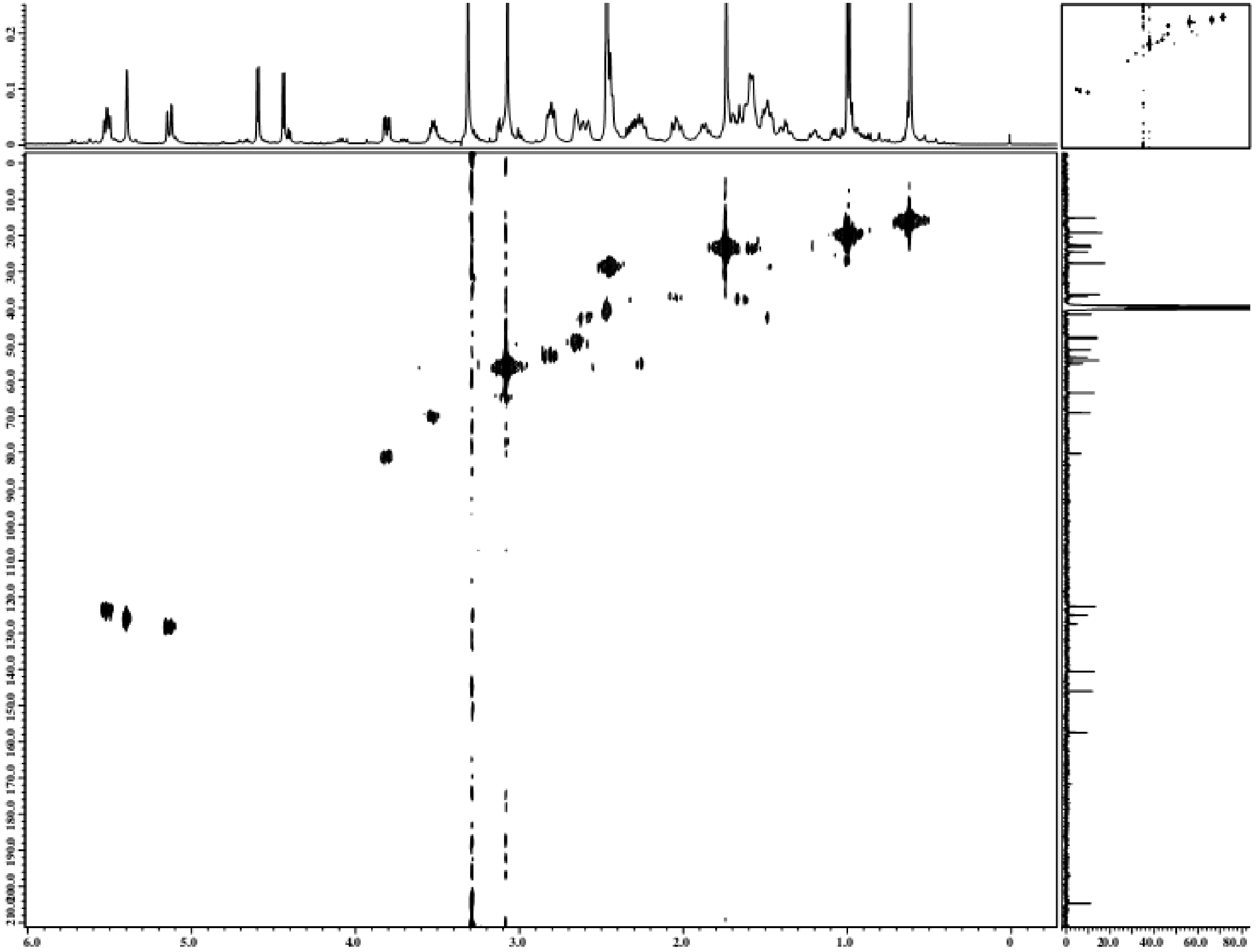

I

Figure S10. *Cont.*

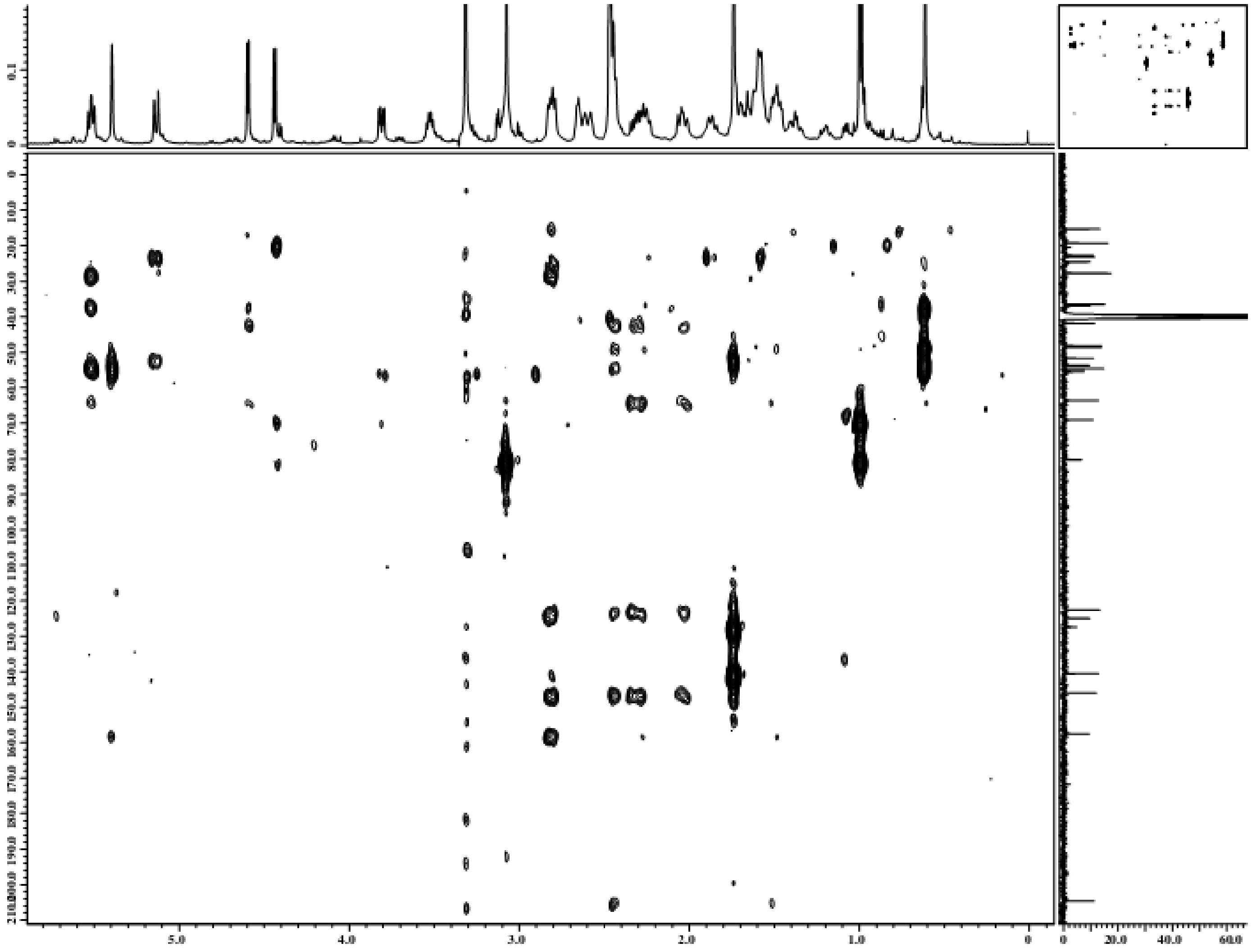

J

Figure S10. *Cont.*

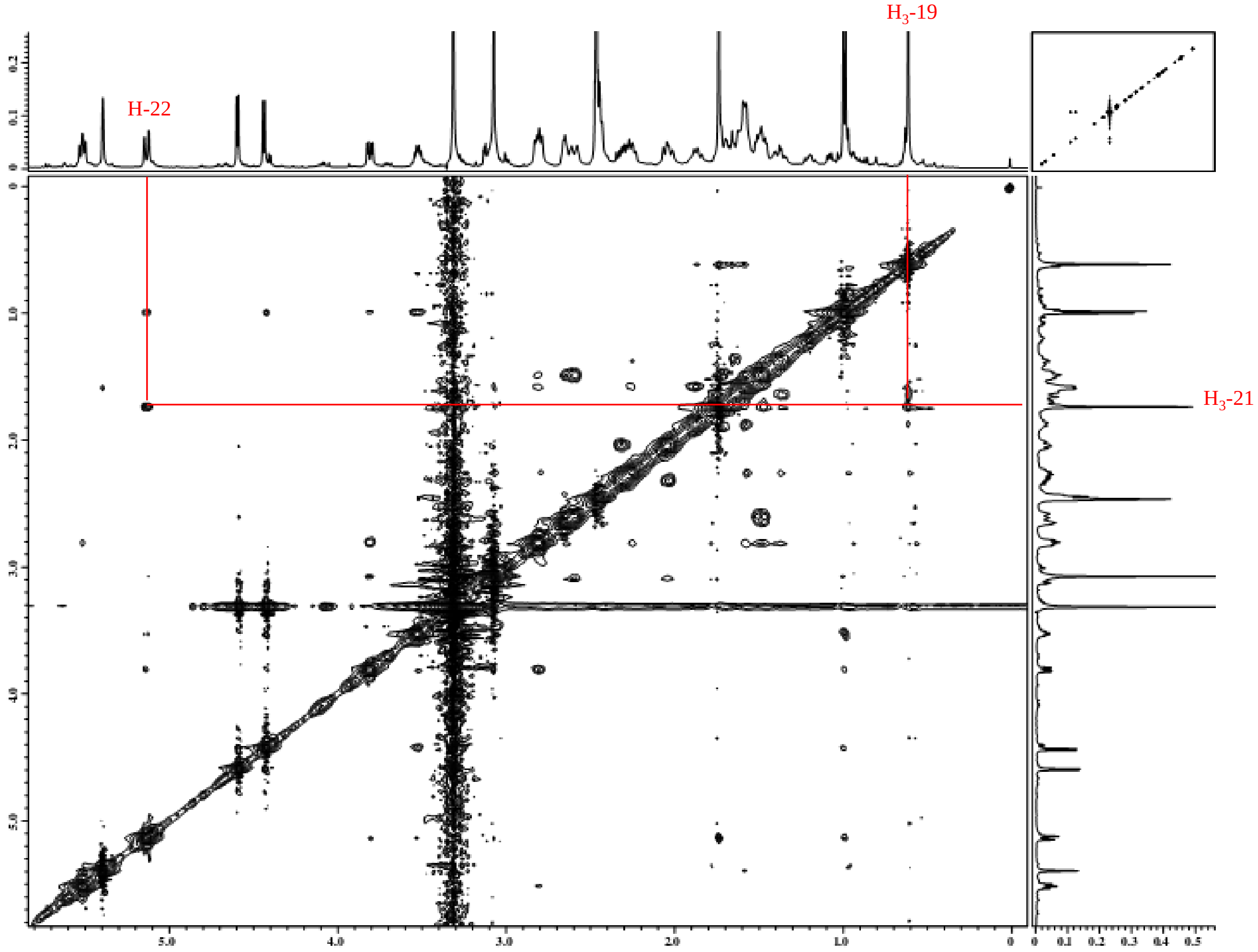

K

Figure S10. *Cont.*

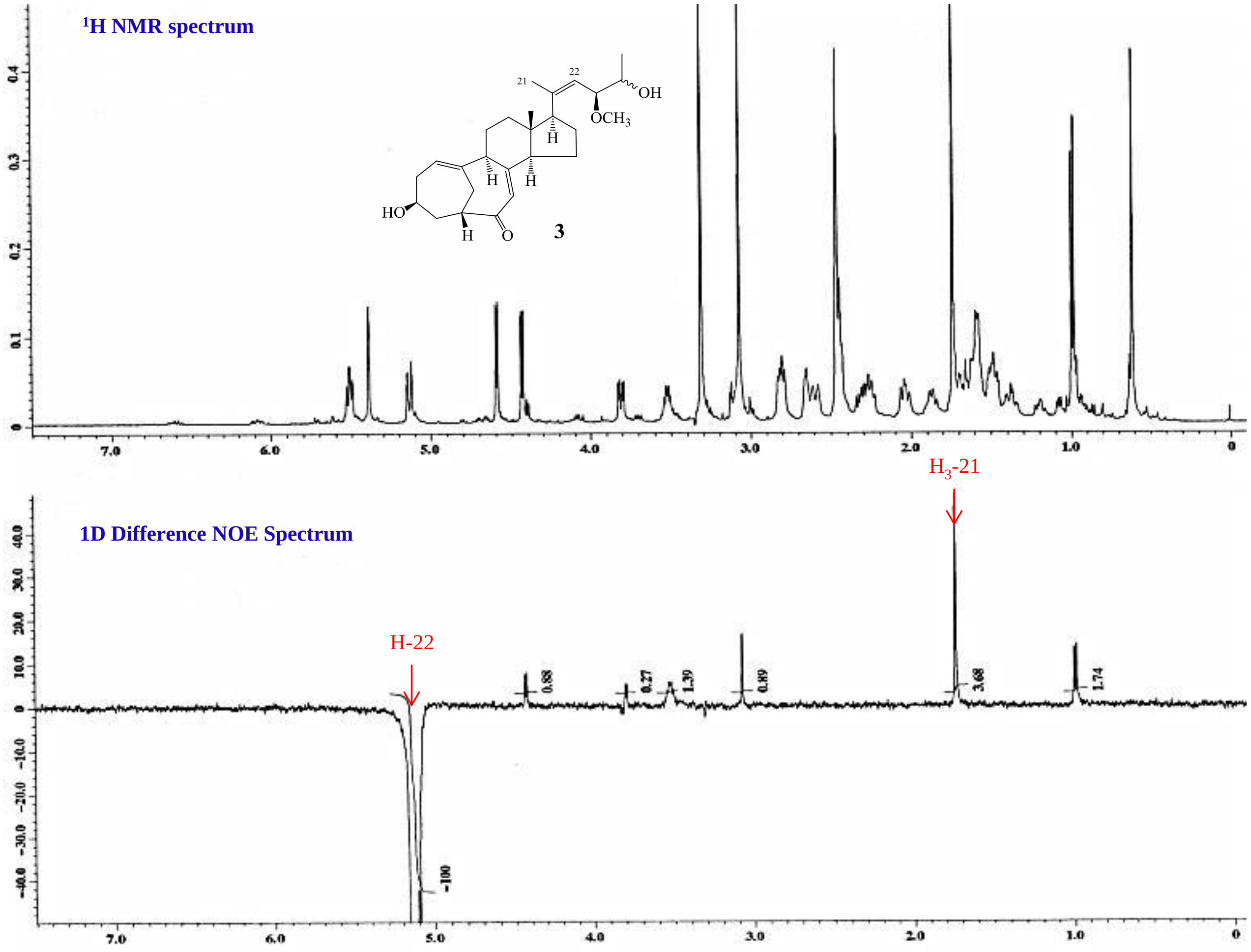

L

© 2014 by the authors; licensee MDPI, Basel, Switzerland. This article is an open access article distributed under the terms and conditions of the Creative Commons Attribution license (http://creativecommons.org/licenses/by/3.0/).
